# Supplementary material for: Regioselective functionalization of aryl azoles as powerful tool for the synthesis of pharmaceutically relevant targets
Source: Nat Commun. 2020 Sep 7;11:4443. doi: 10.1038/s41467-020-18188-z (PMC7477575; doi:10.1038/s41467-020-18188-z)
Supplement: Supplementary file 1 — Supplementary Information [file 41467_2020_18188_MOESM1_ESM.pdf]

Supplementary Information

Mild and Regioselective Magnesiation and Functionalization of Aryl Azoles as a Powerful Tool for the Synthesis of Pharmaceutically Relevant Targets

Lutter et al.

Supplementary Methods

General Information

Unless otherwise indicated, all reactions were carried out with magnetic stirring and in flame-dried glassware under argon. Syringes used to transfer reagents and solvents were purged with argon prior to use. Commercially available starting materials were used without further purification. TMPH was distilled from CaH_2 under argon prior to use. THF was used from Acros, 99.5%, extra dry over molecular sieves. Pd(dppf)Cl_2 was used from SigmaAldrich. Bu_2Mg was used from Abermale and titrated against iodide (50-100 mg) in LiCl THF solution (2 mL, 0.5 M).

All reactions were monitored by gas chromatography (GC and GC-MS) or thin layer chromatography (TLC). TLCs were performed using aluminum plates covered with SiO_2 (Merck 60, F-254) and visualized by UV detection. Purification *via* column chromatography was performed using Merck silica gel 60 (40–63 mm 230–400 mesh ASTM from Merck Melting points were measured using a Büchi B-540 apparatus and are uncorrected. NMR spectra were recorded in $CDCl_3$. Chemical shifts (δ) are reported in parts per million (ppm). Mass spectra and high-resolution mass spectra (HR-MS) were recorded using electroionization (EI) except where otherwise noted. GCs were recorded on machines of the type Hewlett-Packard 6890 (Hewlett Packard, 5% phenylmethylpolysiloxane; length: 15 m, diameter: 0.25 mm; film thickness: 0.25 µm). Infrared spectra were recorded on a *Perkin* 281 IR spectrometer and samples were measured neat (ATR, Smiths Detection DuraSample IR II Diamond ATR). The absorption bands are reported in wave numbers (cm^{-1}).

Preparation of Starting Materials

Preparation of Triazoles – General procedure TP1

A glass flask of suitable size equipped with a magnetic stirring bar, nitrogen inlet tube and a septum cap was charged with a commercially available (substituted) azidobenzene solution (approximately 0.5 M in MTBE or MeTHF). The flask was immersed in a water bath kept at room temperature. A brisk stream of dry nitrogen was bubbled through the solution to evaporate the solvent until the volume of the solution approximately halved. Then, acetonitrile was added to restore the original volume of the solution and nitrogen was again bubbled until the volume approximately halved. Dilution with MeCN and evaporation was repeated twice and finally the volume was reduced so as to obtain a solution of the (substituted) azidobenzene having a concentration of approximately 20% (w/V) in acetonitrile. Then, while keeping the inert atmosphere, the alkyne (3.0 equiv) and *N,N*-diisopropylethylamine (DIPEA, 0.5 equiv) were added by syringe, followed by the addition of CuI (0.10 equiv). The resulting mixture was stirred at room temperature until LC analysis showed complete conversion (12-72 h).

Finely ground 1,3,5-triazine-2,4,5-trithiol trisodium salt hydrate (Na_3TMT, approximately 55% assay, 100 mg per mmol of azidobenzene) was added and the mixture was stirred at room temperature for at least 1 h to precipitate copper in the form of insoluble complexes. The mixture was filtered through a short pad of diatomaceous earth, the filter pad washed with MTBE and the combined filtrates were evaporated to dryness. The residue was purified as detailed below for each compound to give the required triazole.

1-(4-Chlorophenyl)-4-(trimethylsilyl)-1*H*-1,2,3-triazole (2a)

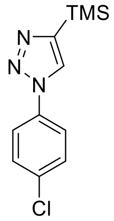

The title compound was prepared according to **TP1** from commercially available 1-azido-4-chlorobenzene solution (0.54 M in MTBE, 10 mL, 5.37 mmol, 1.0 euqiv) and ethynyltrimethylsilane (1.58 g, 16.1 mmol, 3.0 equiv) and was obtained in the form of straw-colored plates (1.08 g, 4.29 mmol, 80% yield). Spectral data were in agreement with the literature.[1]

Purification: recrystallization from *i*PrOH/water (50:50, 20 mL, from reflux temperature to 20 °C).

^{1}H-NMR (300 MHz, $CDCl_3$, ppm): δ = 7.93 (s, 1H), 7.67–7.73 (m, 2H), 7.46 – 7.53 (m, 2H), 0.38 (m, 9H)

^{13}C-NMR (75 MHz, $CDCl_3$, ppm): δ = 147.6, 135.5, 134.0, 129.7, 126.9, 121.8, −1.3.

MS (ESI+): *m/z* (%) = 252.1 $[M+H]^+$.

1-(4-Fluorophenyl)-4-(trimethylsilyl)-1*H*-1,2,3-triazole (2b)

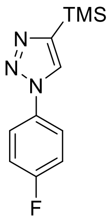

The title compound was prepared according to **TP1** from commercially available 1-azido-4-fluorobenzene solution (0.5 M in MTBE, 21.5 mL, 10.8 mmol,1.0 euqiv) and ethynyltrimethylsilane (3.18 g, 32.4 mmol, 3.0 equiv) and was obtained in the form of beige plates (2.34 g, 9.94 mmol, 92% yield). Spectral data were in agreement with the literature.[2]

Purification: recrystallization from *i*PrOH/water (50:50, 25 mL, from reflux temperature to –20 °C).

^{1}H-NMR (300 MHz, $CDCl_3$, ppm): δ = 7.90 (s, 1H), 7.66 – 7.78 (m, 2H), 7.26 – 7.16 (m, 2H), 0.38 (s, 9H)

^{19}F{^{1}H}-NMR (282 MHz, $CDCl_3$, ppm): δ = −112.7.

^{13}C-NMR (75 MHz, $CDCl_3$, ppm): δ = 162.3 (d, $^{1}J_{CF}$ = 248.4 Hz), 147.5, 133.4 (d, $^{4}J_{CF}$ = 2.3) Hz, 127.3, 122.7 (d, $^{3}J_{CF}$ = 8.30 Hz), 116.6 (d, $^{2}J_{CF}$ = 23.4 Hz), 77.2, −1.2.

MS (ESI+): *m/z* (%) = 236.1 $[M+H]^+$.

1-Phenyl-4-(trimethylsilyl)-1*H*-1,2,3-triazole (2c)

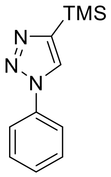

The title compound was prepared according to **TP1** from commercially available azidobenzene solution (0.5 M in MeTHF, 10 mL, 5.0 mmol, 1.0 euqiv) and ethynyltrimethylsilane (1.47 g, 15 mmol, 3.0 equiv) and was obtained in the form of light brown plates (460 mg, 2.12 mmol, 42% yield). Spectral data were in agreement with the literature.[3]

Purification: recrystallization from *i*PrOH/water (50:50, 10 mL, from reflux temperature to –20 °C).

^{1}H-NMR (300 MHz, $CDCl_3$, ppm): δ = 7.95 (s, 1H), 7.75 (d, J = 7.7 Hz, 2H), 7.52 (t, J = 7.7 Hz, 2H), 7.43 (t, J = 7.7 Hz, 1H), 0.39 (s, 9H).

^{13}C-NMR (75 MHz, $CDCl_3$, ppm): δ = 147.3, 137.1, 129.7, 128.5, 127.1, 120.8, −1.2.

MS (ESI+): *m/z* (%) = 218.1 $[M+H]^+$.

1-(*p*-Tolyl)-4-(trimethylsilyl)-1*H*-1,2,3-triazole (2d)

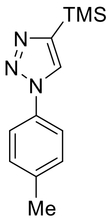

The title compound was prepared according to **TP1** from commercially available 4-azidotoluene solution (0.5 M in MTBE, 10 mL, 5.0 mmol, 1.0 euqiv) and ethynyltrimethylsilane (1.47 g, 15 mmol, 3.0 equiv) and was obtained in the form of light brown needles (710 mg, 3.07 mmol, 61% yield). Spectral data were in agreement with the literature.[1]

Purification: recrystallization from *i*PrOH/water (50:50, 10 mL, from reflux temperature to −20 °C).

^{1}H-NMR (300 MHz, $CDCl_3$, ppm): *δ* = 7.91 (br s, 1H), 7.62 (d, *J* = 8.4 Hz, 2H), 7.32 (d, *J* = 8.4 Hz, 2H), 2.43 (s, 3H), 0.39 (s, 9H).

^{13}C-NMR (75 MHz, $CDCl_3$, ppm): *δ* = 138.6, 134.8, 130.2, 127.2, 120.7, 77.2, 21.1, −1.1.

MS (ESI+): *m/z* (%) = 232.1 $[M+H]^+$.

1-(4-Methoxyphenyl)-4-(trimethylsilyl)-1*H*-1,2,3-triazole (2e)

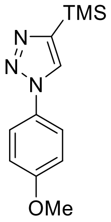

The title compound was prepared according to **TP1** from commercially available 4-azidoanisole solution (0.5 M in MTBE, 10 mL, 5.0 mmol, 1.0 euqiv) and ethynyltrimethylsilane (1.47 g, 15 mmol, 3.0 equiv) and was obtained as a solid (900 mg, 3.64 mmol, 73% yield). Spectral data were in agreement with the literature.[3]

Purification: recrystallization from boiling heptane (5 mL, from reflux to room temperature) provided the title compound in the form of light brown plates (680 mg, 2.75 mmol, 55%). The mother liquor was evaporated to dryness and the residue was purified by flash chromatography on silica gel (eluent: hexanes/EtOAc 80:20) to provide a further portion of the target compound in the form of an orange solid (220 mg, 0.889 mmol, 18%).

^{1}H-NMR (300 MHz, $CDCl_3$, ppm): *δ* = 7.86 (s, 1H), 7.63 (d, *J* = 9.0 Hz, 2H), 7.03 (d, *J* = 9.0 Hz, 2H), 3.88 (s, 3H), 0.39 (s, 9H).

^{13}C-NMR (75 MHz, $CDCl_3$, ppm): *δ* = 159.6, 147.0, 130.5, 127.3, 122.4, 114.7, 55.6, −1.1.

MS (ESI+): *m/z* (%) = 248.1 $[M+H]^+$.

1-(3-Methoxyphenyl)-4-(trimethylsilyl)-1*H*-1,2,3-triazole (2f)

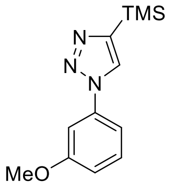

The title compound was prepared according to **TP1** from commercially available 3-azidoanisole solution (0.5 M in MTBE, 30 mL, 15.0 mmol, 1.0 euqiv) and ethynyltrimethylsilane (4.41 g, 45 mmol, 3.0 equiv) was obtained as a brown oil (3.64 g, 14.7 mmol, 98% yield).

Purification: flash chromatography on silica gel (eluent: hexanes/EtOAc 90:10).

m.p.: 38.4 – 40.0 °C.

^{1}H-NMR (300 MHz, $CDCl_3$, ppm): δ = 8.01 (s, 1H), 7.30 – 7.37 (m, 2H), 7.28 – 7.22 (m, 1H), 6.86–6.94 (m, 1H), 3.81 (s, 3H), 0.36 (s, 9H).

^{13}C-NMR (75 MHz, $CDCl_3$, ppm): δ = 160.2, 146.9, 137.8, 130.1, 127.1, 113.9, 112.3, 106.3, 55.2, −1.4.

FT-IR (ATR, cm^{-1}): $\tilde{\nu}$ = 295, 1609, 1594, 1500, 1487, 1466, 1440, 1410, 1384, 1314, 1279, 1246, 1235, 1201, 1179, 1136, 1093, 1049, 1041, 997, 991, 997, 869, 838, 818, 769, 756, 707, 695, 680.

MS (ESI+): *m/z* (%) = 248.2 $[M+H]^+$.

MS (EI, 70 eV): *m/z* (%) =219 (119, 209 (16), 208 (100), 177 (9).

HR-MS (EI, 70 eV): [$C_{12}H_{17}N_3OSi$], calcd.: 247.1141; found: 247.1225.

1-(2-Fluorophenyl)-4-(trimethylsilyl)-1*H*-1,2,3-triazole (2g)

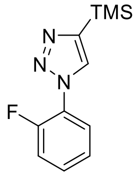

The title compound was prepared according to **TP1** from commercially available 1-azido-2-fluorobenzene solution (0.5 M in MTBE, 30 mL, 15.0 mmol, 1.0 euqiv) and ethynyltrimethylsilane (4.41 g, 45 mmol, 3.0 equiv) and was obtained as a pale yellow oil (3.18 g, 13.5 mmol, 90% yield). Spectral data were in agreement with the literature.[2]

Purification: flash chromatography on silica gel (eluent: hexanes/EtOAc 9:1).

^{1}H-NMR (300 MHz, $CDCl_3$, ppm): δ = 7.86 (d, J = 2.8 Hz, 1H), 7.79 – 7.70 (m, 1H), 7.16 – 7.28 (m, 1H), 7.03 – 7.16 (m, 2H), 0.20 (s, 9H).

^{19}F{^{1}H}-NMR (282 MHz, $CDCl_3$, ppm): δ = −123.6.

^{13}C-NMR (75 MHz, $CDCl_3$, ppm): δ = 153.3 (d, $^{1}J_{CF}$ = 250.7 Hz), 146.8, 129.9 (d, $^{3}J_{CF}$ = 7.6 Hz), 129.8 (d, $^{3}J_{CF}$ = 7.6 Hz), 125.2 (d, $^{2}J_{CF}$ = 10.6 Hz), 125.04 (d, $^{4}J_{CF}$ = 3.0 Hz), 125.01, 116.8 (d, $^{2}J_{CF}$ = 19.6 Hz), −1.3.

MS (ESI+): *m/z* (%) = 236.1 $[M+H]^+$.

Preparation of Organometallic Reagents and Optimization of the Metalation

Preparation of TMPMgBu

In a dry and and argon-flushed *Schlenk*-flask, TMPH (1.18 ml, 7.00 mmol, 1.00 equiv) was placed and Bu_2Mg (9.86 ml, 7.00 mmol, 1.00 equiv, 0.71 M in hexane) was added at 0 °C. The mixture was stirred for 48 h at room temperature resulting in a pale yellow solution. The base was titrated against benzoic acid (80-100 mg) using 4-(phenylazo)diphenylamine (2 mg) as indicator in THF (2 ml). The yield was determined to be 94-98% yield (0.74-0.81 M).

Preparation of $ZnCl_2$ solution in THF (1 M)

In a dry and and argon flushed *Schlenk*-flask $ZnCl_2$ (40.9 g, 300 mmol) was dried under high vacuum at 150 °C for 4 h. After cooling to 25 °C, anhydrous THF was added until a total volume of 30 0mL was reached. The suspension was left stirring overnight at 25 °C and after 12 h the salts had completely dissolved. The stirring was stopped and the solution was left for some hours to become completely clear. The solution was stored over 4 Å MS under argon upon use.

CuCN·2LiCl solution in THF (1 M)[4]

LiCl (8.40 g, 200 mmol) and CuCN (8.96 g, 100 mmol) were dried in a *Schlenk*-flask under high vacuum at 150 °C for 4 h. After cooling to 25 °C, dry THF was added until a total volume of 100 mL was reached. The suspension was left stirring overnight at rt until all salts had completely dissolved. The solution was stored under argon upon use.

Directed *ortho*-metalation (TP2)

The corresponding substrate was placed in a dry and argon flushed 10 mL *Schlenk*-tube equipped with a magnetic stirring bar and a septum and was suspended in toluene (1.00 M). TMPMgBu (1.0 equiv) was added and the mixture was stirred for the appropriate time. To monitor the process of metalation aliquots quenched with D_2O were analyzed using ^{1}H-NMR.

(5-Chloro-2-(4-(trimethylsilyl)-1*H*-1,2,3-triazol-1-yl)phenyl)magnesium reagent (3a)

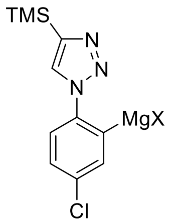

The title compound was prepared according to **TP2** from 1-(4-chlorophenyl)-4-(trimethylsilyl)-1*H*-1,2,3-triazole (**2a**, 126 mg, 0.5 mmol) affording the corresponding magnesium reagent in 81% yield. The yield was determined *via* deuterolysis.

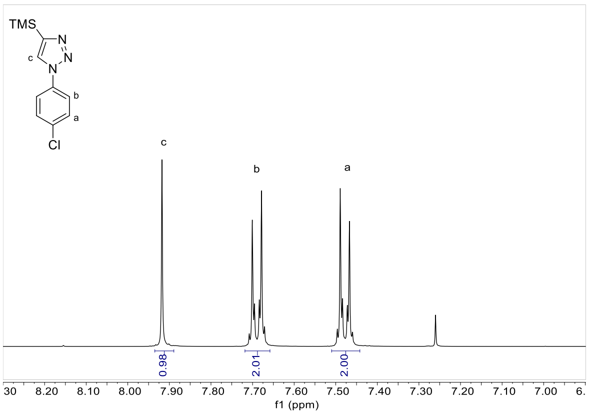

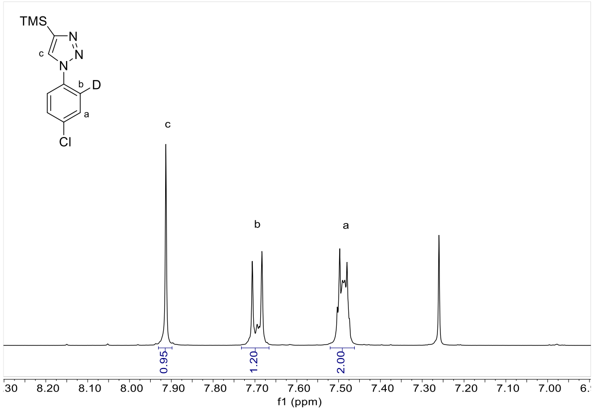

Supplementary Figure 1: ^{1}H-NMR spectra of **2a** (left) and after deutorolysis of **3a** (right).

Supplementary Table 1: Optimization of the metalation of **2a**.

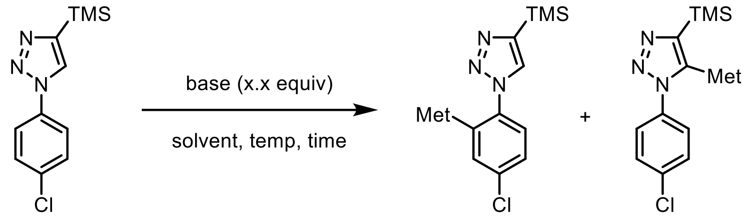

2a **A** **B**

[M] = Li or MgX

entry	base	equivalents	solvent	temperature	time	**A**	**B**
1	TMPLi	1.2	THF	-78 °C	0.5 h	0%	99%
2	TMPMgCl·LiCl	1.2	THF	rt	1 h	19%	39%
3	$TMP_2Zn·2MgCl_2·2LiCl$	1.2	THF	0 °C	1 h	0%	0%
4	TMP_2Mg	1.2	THF	rt	1 h	32%	67%
5	TMPMgBu	1.0	THF	-40 °C	0.5 h	35%	3%
6	TMPMgBu	1.0	THF	-20 °C	0.5 h	57%	7%
7	TMPMgBu	1.0	THF	0 °C	0.5 h	63%	8%
8	TMPMgBu	1.0	THF	rt	0.5 h	68%	11%
9	TMPMgBu	1.0	THF	rt	1 h	75%	15%
10[a]	TMPMgBu	1.0	THF	rt	4 h	78%	32%
11[a]	TMPMgBu	1.0	THF	rt	6 h	76%	38%
12[a]	TMPMgBu	1.2	THF	rt	0.5 h	78%	28%
13[a]	TMPMgBu	1.2	THF	rt	1 h	78%	40%
14	TMPMgBu	1.0	toluene	rt	0.5 h	70%	2%
15	TMPMgBu	1.0	toluene	rt	1 h	81%	3%
16	TMPMgBu	1.0	toluene	rt	3 h	81%	3%
17	TMPMgBu	1.0	toluene	40°C	1 h	84%	6%
18	TMPMgBu	1.2	toluene	rt	1 h	85%	3%
19	TMPMgBu	0.8	toluene	rt	1 h	67%	2%
20	TMPMgBu	1.0	hexane	rt	1 h	75%	2%
21	TMPMgBu	1.0	fluorobenzene	rt	1 h	79%	2%
22	TMPMgBu	1.0	benzotrifluoride	rt	1 h	80%	2%
23	Cy_2NMgBu	1.0	toluene	rt	1 h	65%	2%
24	$i\text{Pr}_2NMgBu$	1.0	toluene	rt	1 h	67%	3%
25[a]	Bu_2Mg	1.0	toluene	rt	1 h	93%	10%
26	TMP_2Mg	1.2	toluene	rt	1 h	decomposition	

[a] for values A+B > 100% bis-metalated species was observed.

(5-Fluoro-2-(4-(trimethylsilyl)-1*H*-1,2,3-triazol-1-yl)phenyl)magnesium reagent (3b)

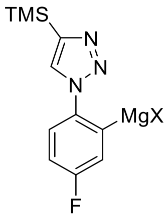

The title compound was prepared according to **TP2** from 1-(4-fluorophenyl)-4-(trimethylsilyl)-1*H*-1,2,3-triazole (**2b**, 118 mg, 0.5 mmol) affording the corresponding magnesium reagent in 86% yield. The yield was determined *via* deuterolysis.

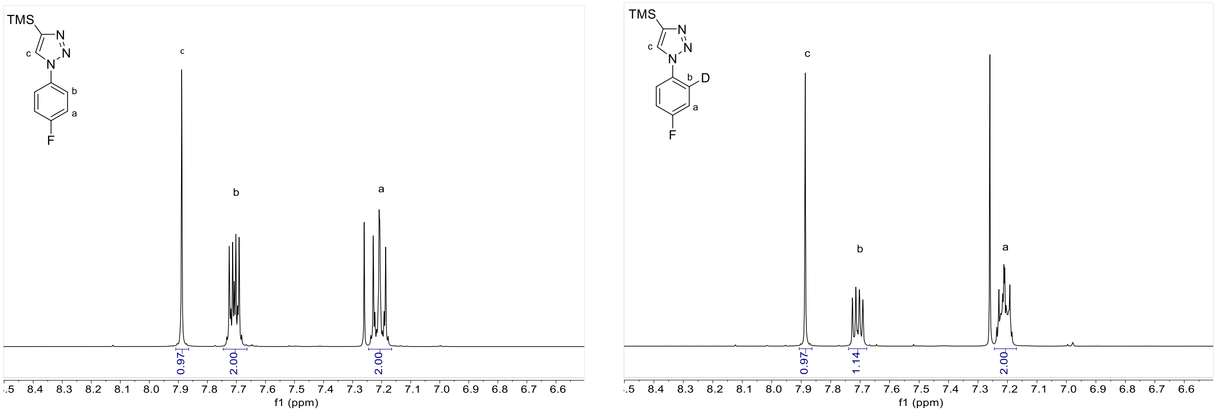

Supplementary Figure 2: ^{1}H-NMR spectra of **2b** (left) and after deutorolysis of **3b** (right).

(2-(4-(Trimethylsilyl)-1*H*-1,2,3-triazol-1-yl)phenyl)magnesium reagent (3c)

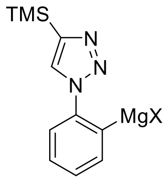

The title compound was prepared according to **TP2** from 1-phenyl-4-(trimethylsilyl)-1*H*-1,2,3-triazole (**2c**, 109 mg, 0.5 mmol) affording the corresponding magnesium reagent in 72% yield. The yield was determined *via* deuterolysis.

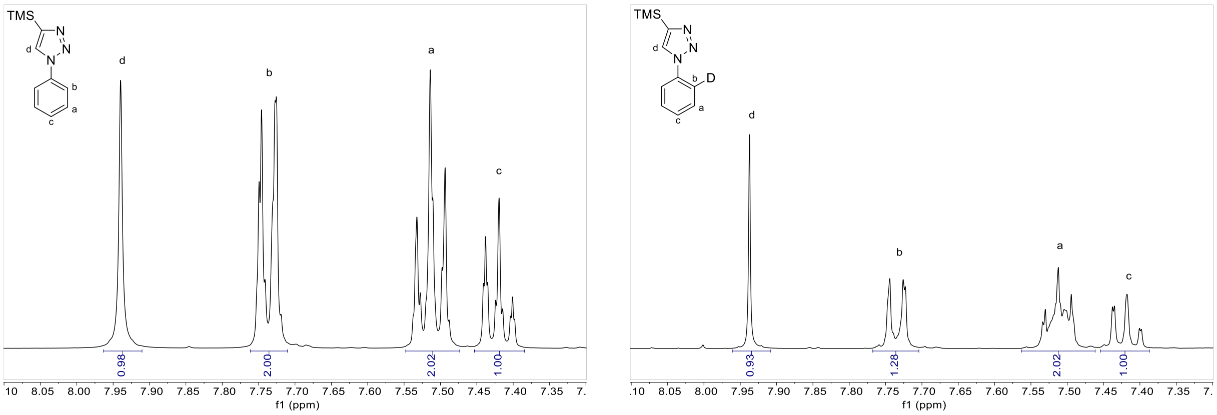

Supplementary Figure 3: ^{1}H-NMR spectra of **2c** (left) and after deutorolysis of **3c** (right).

(5-Methyl-2-(4-(trimethylsilyl)-1*H*-1,2,3-triazol-1-yl)phenyl)magnesium reagent (3d)

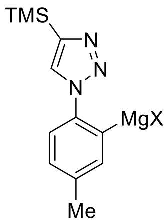

The title compound was prepared according to **TP2** from 1-(*p*ara-tolyl)-4-(trimethylsilyl)-1*H*-1,2,3-triazole (**2d**, 116 mg, 0.5 mmol) affording the corresponding magnesium reagent in 68% yield. The yield was determined *via* deuterolysis.

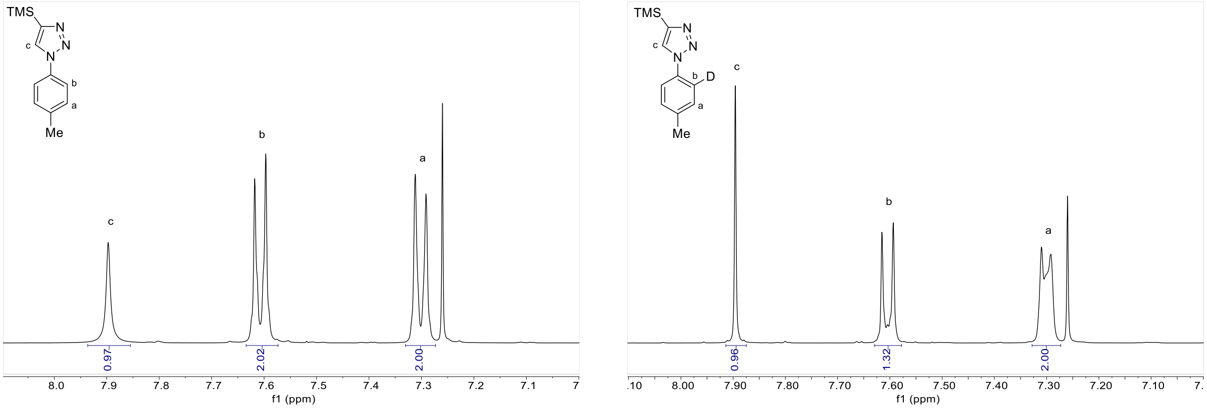

Supplementary Figure 4: ^{1}H-NMR spectra of **2d** (left) and after deutorolysis of **3d** (right).

(5-Methoxy-2-(4-(trimethylsilyl)-1*H*-1,2,3-triazol-1-yl)phenyl)magnesium reagent (3e)

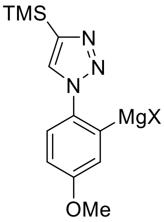

The title compound was prepared according to **TP2** from 1-(4-methoxyphenyl)-4-(trimethylsilyl)-1*H*-1,2,3-triazole (**2e**, 124 mg, 0.5 mmol) affording the corresponding magnesium reagent in 77% yield. The yield was determined *via* deuterolysis.

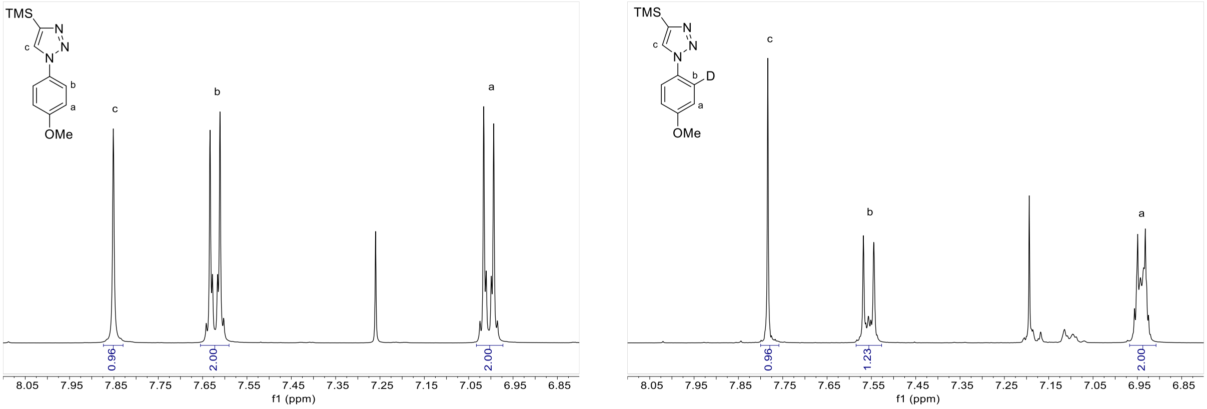

Supplementary Figure 5: ^{1}H-NMR spectra of **2e** (left) and after deutorolysis of **3e** (right).

(2-Methoxy-6-(4-(trimethylsilyl)-1*H*-1,2,3-triazol-1-yl)phenyl)magnesium reagent (3f)

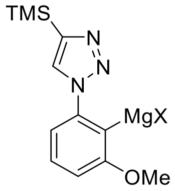

The title compound was prepared according to **TP2** from 1-(3-methoxyphenyl)-4-(trimethylsilyl)-1*H*-1,2,3-triazole (**2f**, 124 mg, 0.5 mmol) affording the corresponding magnesium reagent in 70% yield. The yield was determined *via* deuterolysis.

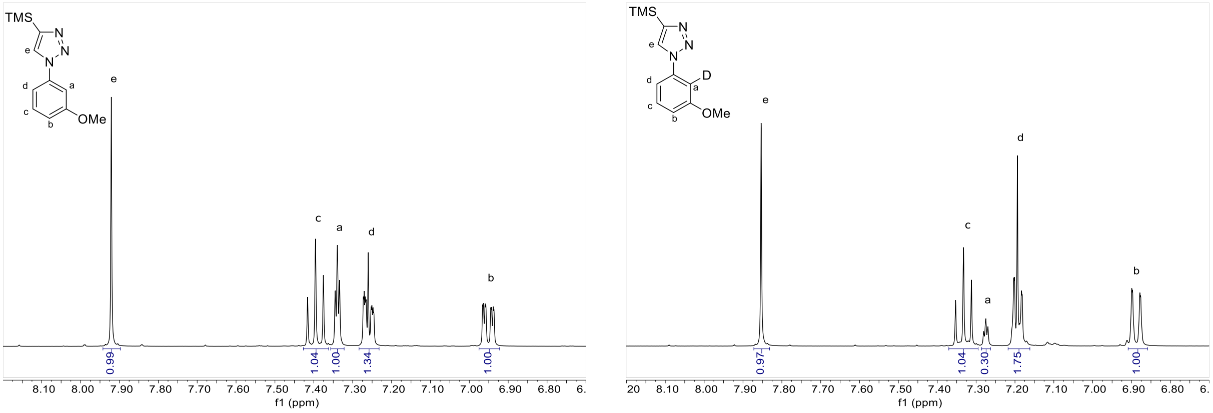

Supplementary Figure 6: ^{1}H-NMR spectra of **2f** (left) and after deutorolysis of **3f** (right).

(3-Fluoro-2-(4-(trimethylsilyl)-1*H*-1,2,3-triazol-1-yl)phenyl)magnesium reagent (3g)

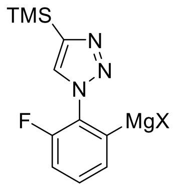

The title compound was prepared according to **TP2** from 1-(2-fluorophenyl)-4-(trimethylsilyl)-1*H*-1,2,3-triazole (**2g**, 118 mg, 0.5 mmol) affording the corresponding magnesium reagent in 77% yield. The yield was determined *via* deuterolysis.

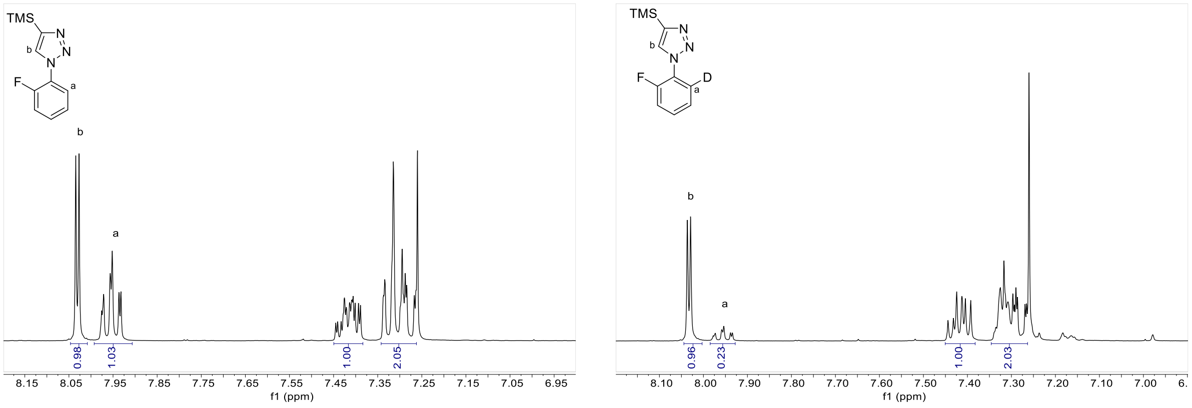

Supplementary Figure 7: ^{1}H-NMR spectra of **2g** (left) and after deutorolysis of **3g** (right).

(5-Chloro-2-(3,5-dimethyl-1*H*-pyrazol-1-yl)phenyl)magnesium reagent (5a)

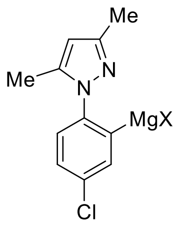

The title compound was prepared according to **TP2** from 1-(4-chlorophenyl)-3,5-dimethyl-1*H*-pyrazole (**4a**, 104 mg, 0.5 mmol) affording the corresponding magnesium reagent in 82% yield. The yield was determined *via* deuterolysis.

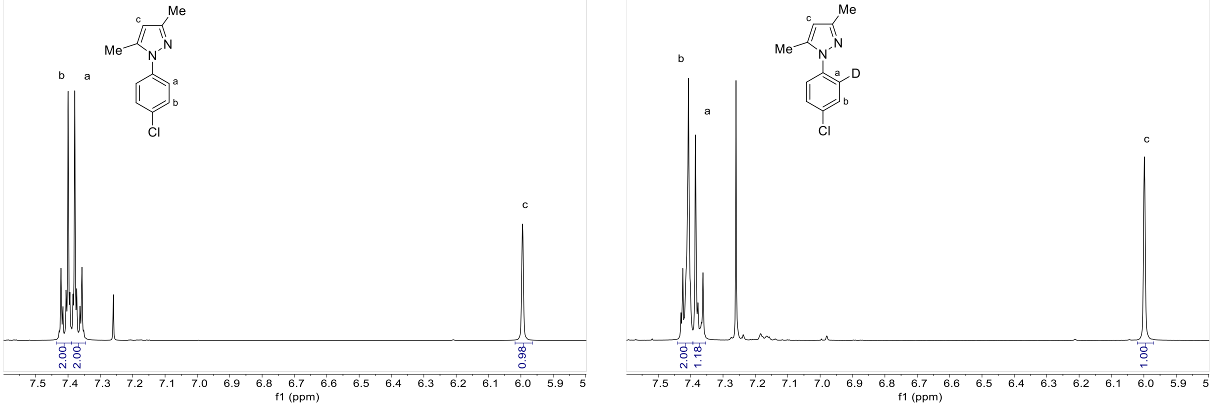

Supplementary Figure 8: ^{1}H-NMR spectra of **4a** (left) and after deutorolysis of 5**a** (right).

(5-Chloro-2-(1*H*-pyrazol-1-yl)phenyl)magnesium reagent (5b)

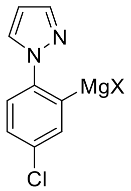

The title compound was prepared according to **TP2** from 1-(4-chlorophenyl)-1H-pyrazole (**4b**, 89 mg, 0.5 mmol) affording the corresponding magnesium reagent in 78% yield. The yield was determined *via* deuterolysis.

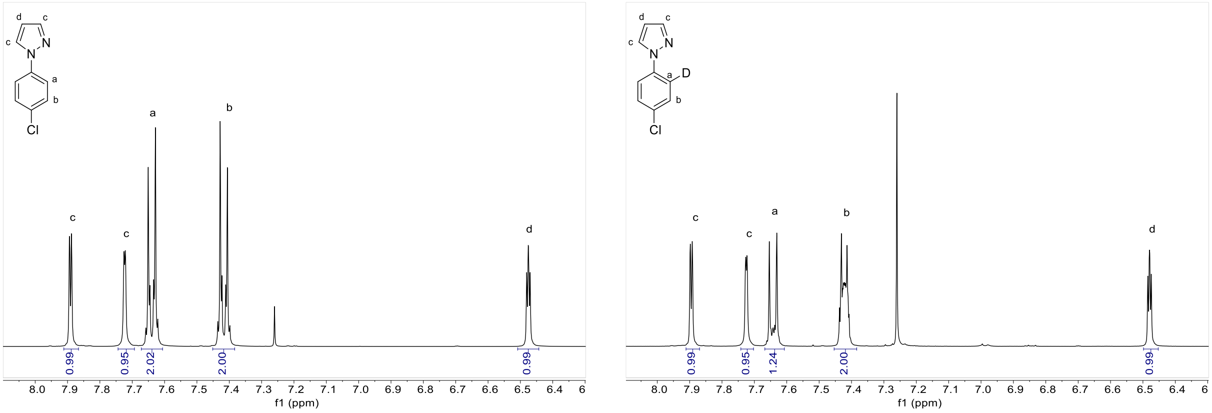

Supplementary Figure 9: ^{1}H-NMR spectra of **4b** (left) and after deutorolysis of 5b (right).

(2-(5-Phenyl-1,3,4-oxadiazol-2-yl)phenyl)magnesium reagent (5c)

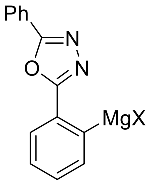

The title compound was prepared according to **TP2** from 2,5-diphenyl-1,3,4-oxadiazole (**4a**, 111 mg, 0.5 mmol) affording the corresponding magnesium reagent in 76% yield. The yield was determined *via* deuterolysis.

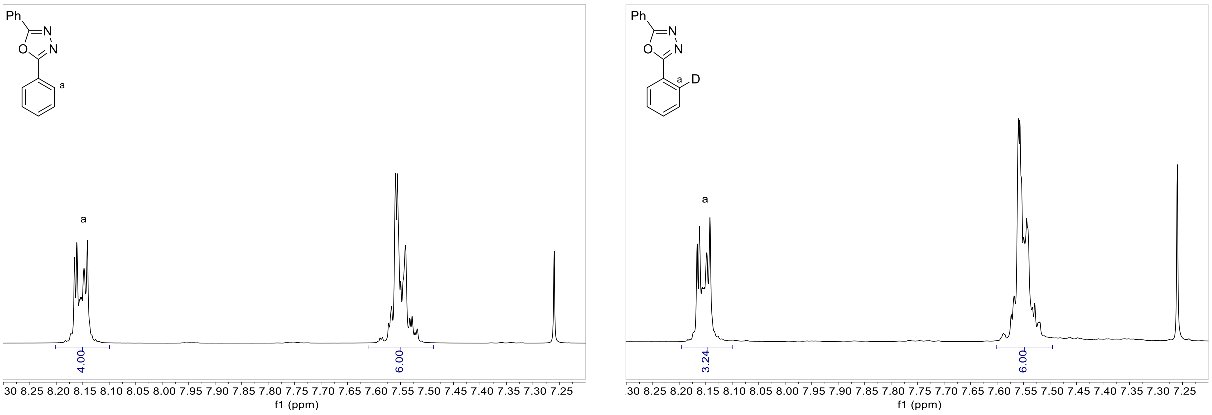

Supplementary Figure 10: ^{1}H-NMR spectra of **4c** (left) and after deutorolysis of 5c (right).

(2-(4,4-Dimethyl-4,5-dihydrooxazol-2-yl)phenyl)magnesium reagent (5d)

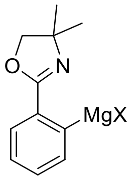

The title compound was prepared according to **TP2** from 4,4-dimethyl-2-phenyl-4,5-dihydrooxazole (**4d**, 88 mg, 0.5 mmol) affording the corresponding magnesium reagent in 77% yield. The yield was determined *via* deuterolysis.

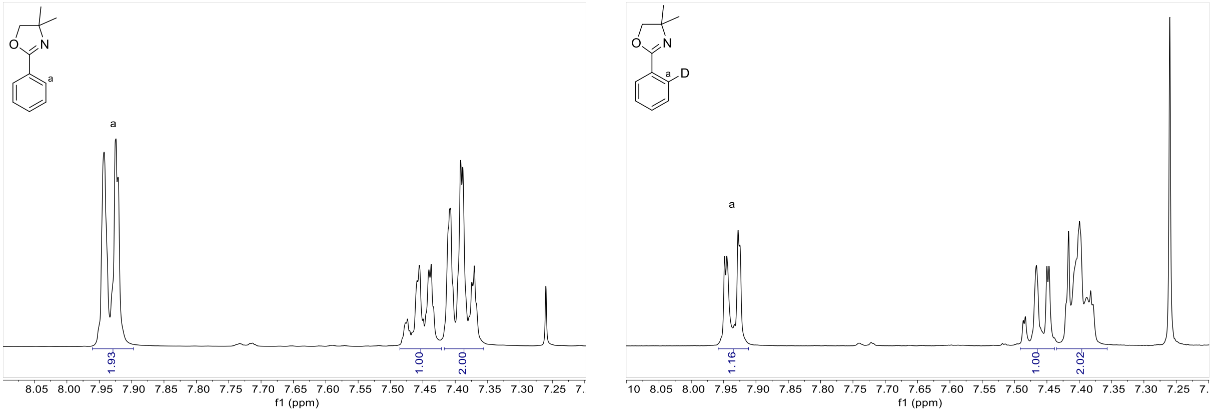

Supplementary Figure 11: ^{1}H-NMR spectra of **4d** (left) and after deutorolysis of 5d (right).

(5-Fluoro-4'-(trifluoromethoxy)-2-(4-(trimethylsilyl)-1*H*-1,2,3-triazol-1-yl)-[1,1'-biphenyl]-3-yl)magnesium reagent (9)

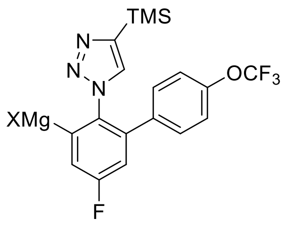

The title compound was prepared according to **TP1** from 1-(5-fluoro-4'-(trifluoromethoxy)-[1,1'-biphenyl]-2-yl)-4-(trimethylsilyl)-1*H*-1,2,3-triazole (**1h**, 99 mg, 0.5 mmol) affording the corresponding magnesium reagent in 80% yield. The yield was determined *via* deuterolysis.

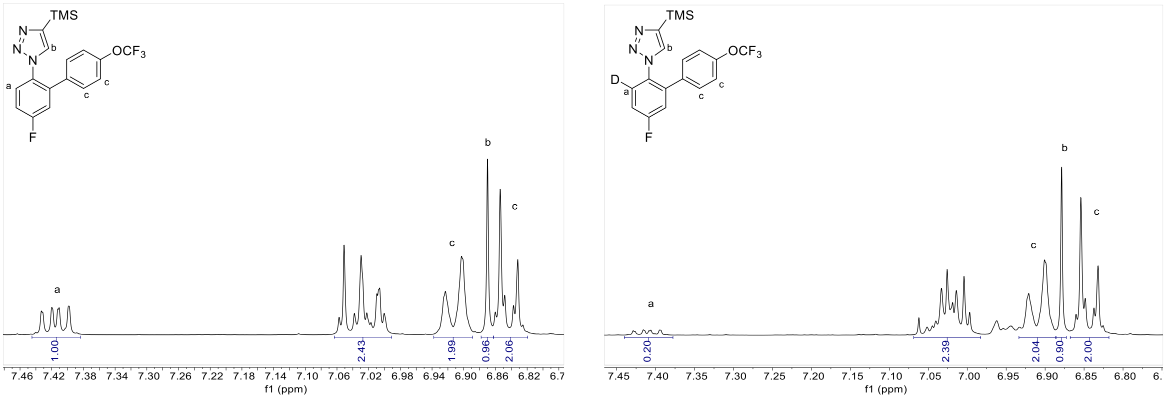

Supplementary Figure 12: ^{1}H-NMR spectra of **1h** (left) and after deutorolysis of **9** (right).

Unsuccesful metalation reactions:

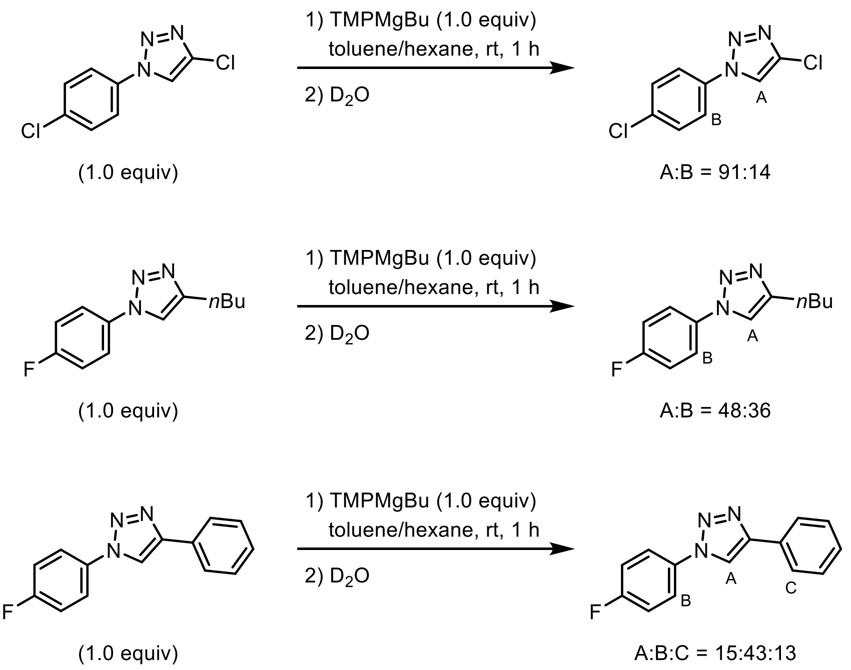

Supplementary Figure 13: Unsuccesful metalation reactions.

Supplementary Table 2: Optimization of the cross-coupling reaction.

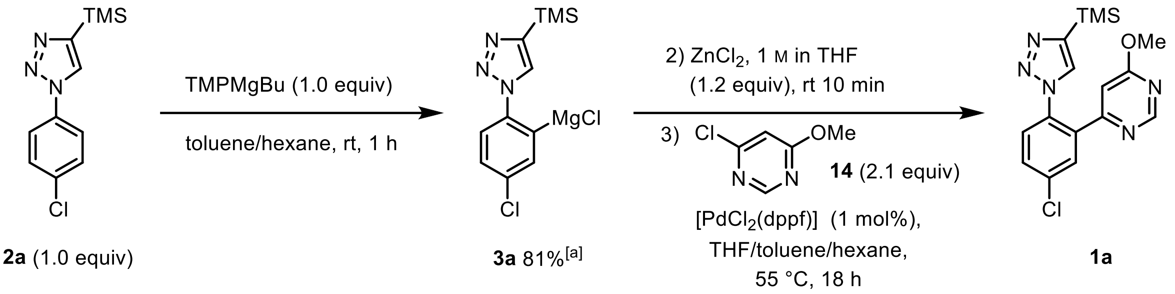

2a (1.0 equiv) **3a** 81%[a] **1a**

entry	variation from standard conditions	yield of **1a**
1	-	86%
2	1.1 equiv of **14**	53%
3	1.5 equiv of **14**	63%
5	no $ZnCl_2$, no $PdCl_2$dppf	0%
6	no $PdCl_2$dppf	0%
7	2.5 equiv of $ZnCl_2$	70%
8	no THF	58%

[a] metalation yiled was determined by ^{1}H-NMR analysis of D_2O-quenched reaction aliquots.

Palladium-catalzed Negishi-cross-coupling of arylmagnesium aryl azoles with aryl bromides TP3

The corresponding arylmagnesium reagent, prepared according to **TP1**, was transmetalated with a $ZnCl_2$ solution (0.5 ml per 0.5 mmol of aryl azole, 1.00 M in THF) and THF (1.0 ml per 0.5 mmol) was added. A dry and argon-flushed *Schlenk*-tube, equipped with a magnetic stirring bar and a septum was charged with Pd(dppf)Cl_2 (1.0 mol%, 0.005 mmol, 3.7 mg) and the respective aryl bromide (1.00 mmol – 1.25 mmol, 1.98 – 2.5 equiv).The freshly prepared arylzinc reagent was added and the reaction mixture was placed in an oil bath at 55 °C. After 16 h, saturated aq. NH_4Cl solution (5 mL) was added, the phases where separated and the aqueous phase was extracted with EtOAc (3 x 25 mL). The combined organic layers were dried over $MgSO_4$. The solvents were removed under reduced pressure and the crude product was subjected to column chromatography on silica yielding the respective title compound.

4-(5-Chloro-2-(4-(trimethylsilyl)-1*H*-1,2,3-triazol-1-yl)phenyl)-6-methoxypyrimidine (1a)

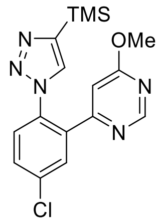

Following **TP3** (5-chloro-2-(4-(trimethylsilyl)-1*H*-1,2,3-triazol-1-yl)phenyl)magnesium reagent (**3a**, 0.405 mmol, 1.00 equiv) was coupled with 4-chloro-6-methoxypyrimidine (0.850 mmol, 123 mg, 2.10 equiv). spectral data were in agreement with the literature.[5]

Isolated yield: 155 mg, 0.431 mmol, 86%, pale yellow crystals

Purification: pentane:ethyl acetate = 7:3.

^{1}H-NMR (400 MHz, CDCl$_3$, ppm): *δ* = 8.68 (s, 1H), 7.80 (s, 1H), 7.47-7.59 (m, 3H), 6.18 (s, 1H), 3.90 (s, 3H), 0.29 (s, 9H).

^{13}C-NMR (100 MHz, CDCl$_3$, ppm): *δ* = 169.8, 161.7, 158.2, 146.9, 136.0, 135.6, 133.7, 131.0, 130.7, 130.4, 128.0, 107.1, 53.9, –1.3.

MS (ESI): m/z (%) = 360.0 [M+H]$^+$.

1-(5-Chloro-4'-methoxy-[1,1'-biphenyl]-2-yl)-4-(trimethylsilyl)-1*H*-1,2,3-triazole (1b)

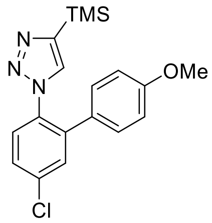

Following **TP3** (5-chloro-2-(4-(trimethylsilyl)-1*H*-1,2,3-triazol-1-yl)phenyl)magnesium reagent (**3a**, 0.405 mmol, 1.00 equiv) was coupled with 1-bromo-4-methoxybenzene (0.850 mmol, 159 mg, 2.10 equiv).

Isolated yield: 122 mg, 0.341 mmol, 84%, colorless crystals

m.p.: 74.1 – 75.6 °C.

Purification: pentane:ethyl acetate = 95:5 to 9:1.

^{1}H-NMR (400 MHz, CDCl$_3$, ppm): *δ* = 7.56 (d, *J* = 8.4 Hz, 1H), 7.49 (d, *J* = 2.3 Hz, 1H), 7.44 (dd, *J* = 8.4, 2.4 Hz, 1H), 7.09 (s, 1H), 6.95 – 6.90 (m, 2H), 6.81 – 6.76 (m, 2H), 3.77 (s, 3H), 0.22 (s, 9H).

^{13}C-NMR (100 MHz, CDCl$_3$, ppm): *δ* = 159.9, 146.6, 138.5, 135.5, 133.8, 131.2, 130.8, 129.7, 128.5, 128.2, 127.9, 114.3, 55.5, −1.1.

FT-IR (ATR, cm^{-1}): $\tilde{\nu}$ = 3105, 2956, 1610, 1514, 1490, 1473, 1463, 1455, 1442, 1404, 1288, 1254, 1241, 1198, 1151, 1124, 1113, 1095, 1037, 1022, 1009, 1000, 982, 906, 819, 775, 762, 755, 724, 711, 680.

MS (EI, 70 eV): *m/z* (%) = 330 (26), 328 (65), 314 (65), 283 (69), 279 (86), 271 (34), 139 (63), 73 (100), 43 (30).

HR-MS (EI, 70 eV): [$C_{18}H_{20}N_3OClSi$], calcd.: 357.1064; found: 357.1051.

1-(3'-(1,3-Dioxolan-2-yl)-[1,1'-biphenyl]-2-yl)-4-(trimethylsilyl)-1*H*-1,2,3-triazole (1c)

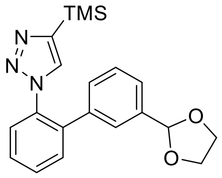

Following **TP3** (2-(4-(trimethylsilyl)-1*H*-1,2,3-triazol-1-yl)phenyl)magnesium reagent (**3c**, 0.360 mmol, 1.00 equiv) was coupled with 2-(3-bromophenyl)-1,3-dioxolane (0.850 mmol, 195 mg, 2.36 equiv).

Isolated yield: 103 mg, 0.281 mmol, 78%, colorless oil.

Purification: pentane:ethyl acetate = 8:2.

^{1}H-NMR (400 MHz, benzene-d$_6$, ppm): δ = 7.19 (dd, *J* = 7.7, 1.6 Hz, 1H), 7.07 (dt, *J* = 7.5, 1.5 Hz, 1H), 7.04 (t, *J* = 1.8 Hz, 1H), 6.89 (d, *J* = 1.5 Hz, 1H), 6.85 – 6.69 (m, 2H), 6.71 – 6.66 (m, 1H), 6.62 (dt, *J* = 7.7, 1.6 Hz, 1H), 6.49 (s, 1H), 5.32 (s, 1H), 3.32 (ddd, *J* = 6.6, 5.1, 2.6 Hz, 2H), 3.21 – 3.13 (m, 2H), 0.00 (s, 9H)

^{13}C-NMR (100 MHz, benzene-d$_6$, ppm): δ = 145.9, 139.6, 138.0, 137.2, 136.0, 131.2, 131.0, 129.5, 129.4, 128.6, 128.6, 127.3, 127.0, 126.4, 103.6, 65.2, -1.0.

FT-IR (ATR, cm^{-1}): $\tilde{\nu}$ = 2956, 2893, 1717, 1504, 1487, 1376, 1248, 1199, 1079, 1054, 1036, 983, 943, 901, 839, 802, 756, 704, 661.

MS (EI, 70 eV): *m/z* (%) = 337 (14), 295 (28), 294 (100), 278 (48), 265 (18), 254 (47), 251 (18), 250 (749, 220 (17), 218 (15), 204 (85), 192 (16), 174 (13), 152 (11), 75 (15), 73 (16).

HR-MS (EI, 70 eV): [$C_{20}H_{23}N_3O_2Si$], calcd.: 365.1560; found: 365.1565.

5'-Chloro-3-fluoro-2'-(4-(trimethylsilyl)-1*H*-1,2,3-triazol-1-yl)-[1,1'-biphenyl]-4-carbonitrile (1e)

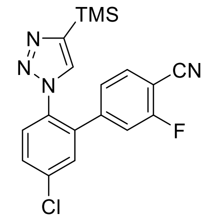

Following **TP3** (5-chloro-2-(4-(trimethylsilyl)-1*H*-1,2,3-triazol-1-yl)phenyl)magnesium reagent (**3a**, 0.405 mmol, 1.00 equiv) was coupled with 4-bromo-2-fluorobenzonitrile (0.850 mmol, 170 mg, 2.10 equiv).

Isolated yield: 131 mg, 0.353 mmol, 87%, orange crystals.

Purification: pentane:ethyl acetate = 95:5 to 9:1.

m.p.: 98.6 – 100.4 °C.

^{1}H-NMR (400 MHz, $CDCl_3$, ppm): δ = 7.59 (dd, *J* = 8.5, 2.1 Hz, 1H), 7.55 (dd, *J* = 8.6, 0.6 Hz, 1H), 7.54 – 7.49 (m, 1H), 7.51 (d, *J* = 2.5 Hz, 1H), 7.29 (s, 1H), 6.95 – 6.92 (m, 1H), 6.92 – 6.89 (m, 1H), 0.27 (s, 9H).

^{13}C-NMR (100 MHz, $CDCl_3$, ppm): δ = 166.0 (d, *J* = 260.8 Hz), 147.6, 143.6 (d, *J* = 8.2 Hz), 136.4, 136.2 (d, *J* = 1.9 Hz), 133.7, 133.6, 130.7 (d, *J* = 10.4 Hz), 130.3, 128.4, 125.0 (d, *J* = 3.7 Hz), 116.6 (d, *J* = 20.8 Hz), 113.4, 101.5 (d, *J* = 15.4 Hz), −1.1.

^{19}F NMR (377 MHz, $CDCl_3$, ppm): δ = −105.16 (dd, *J* = 9.5, 6.5 Hz).

FT-IR (ATR, cm^{-1}): $\tilde{\nu}$ = 2233, 1619, 1556, 1506, 1421, 1245, 1198, 1186, 1145, 1114, 1029, 993, 982, 923, 883, 871, 840, 830, 814, 784, 758, 733, 709, 676, 660.

MS (EI, 70 eV): *m/z* (%) = 343 (20), 330 (35), 329 (29), 327 (22), 293 (41), 234 (14), 195 (24), 77 (30), 73 (40), 45 (20), 43 (22).

HR-MS (EI, 70 eV): [M+H$^+$] = [$C_{18}H_{17}N_4ClFSi^+$], calcd.: 371.0890; found: 371.0898.

3,3'-Difluoro-2'-(4-(trimethylsilyl)-1*H*-1,2,3-triazol-1-yl)-[1,1'-biphenyl]-4-carbonitrile (1e)

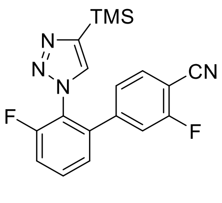

Following **TP3** (3-fluoro-2-(4-(trimethylsilyl)-1*H*-1,2,3-triazol-1-yl)phenyl)magnesium reagent (**3g**, 0.385 mmol, 1.00 equiv) was coupled with 4-bromo-2-fluorobenzonitrile (0.850 mmol, 170 mg, 2.21 equiv).

Isolated yield: 120 mg, 0.339 mmol, 86%, pale yellow oil.

Purification: pentane:ethyl acetate = 8:2.

^{1}H-NMR (400 MHz, $CDCl_3$, ppm): *δ* = 7.62 (td, *J* = 8.1, 5.3 Hz, 1H), 7.54 (d, *J* = 1.1 Hz, 1H), 7.48 (dd, *J* = 8.0, 6.6 Hz, 1H), 7.39 (td, *J* = 8.6, 1.3 Hz, 1H), 7.32 (dt, *J* = 7.9, 1.2 Hz, 1H), 6.97 – 6.87 (m, 2H), 0.31 (s, 9H).

^{13}C-NMR (100 MHz, $CDCl_3$, ppm): *δ* = 162.8 (d, *J* = 260.2 Hz), 157.2 (d, *J* = 255.1 Hz), 147.2, 143.84 (dd, *J* = 8.2, 2.6 Hz), 138.2 (d, *J* = 2.0 Hz), 133.5, 131.8 (d, *J* = 8.6 Hz), 131.7 (d, *J* = 1.2 Hz), 126.0 (d, *J* = 3.6 Hz), 124.9 (d, *J* = 3.6 Hz), 123.7 (d, *J* = 13.7 Hz), 117.4 (d, *J* = 20.1 Hz), 116.5 (d, *J* = 20.8 Hz), 113.6, 101.2 (d, *J* = 15.5 Hz), −1.1.

^{19}F NMR (377 MHz, $CDCl_3$, ppm): δ = −105.64 (dd, *J* = 9.6, 6.6 Hz), −119.96 (dd, *J* = 9.0, 5.4 Hz).

FT-IR (ATR, cm^{-1}): $\tilde{\nu}$ = 2234, 1619, 1589, 1561, 1511, 1497, 1470, 1417, 1387, 1265, 1247, 1201, 1170, 1142, 1125, 1075, 1083, 1034, 999, 985, 958, 835, 797, 763, 744, 721, 711, 701, 665.

MS (EI, 70 eV): *m/z* (%) = 326 (55), 312 (61), 311 (45), 311 (45), 311 (45), 311 (62), 311 (64), 311 (65), 310 (32), 249 (24), 215 (29), 208 (28), 195 (26), 77 (100).

HR-MS (EI, 70 eV): [$C_{18}H_{16}F_2N_4Si$], calcd.: 354.1112; found: 354.1110.

Ethyl 5'-chloro-2'-(4-(trimethylsilyl)-1*H*-1,2,3-triazol-1-yl)-[1,1'-biphenyl]-3-carboxylate (1f)

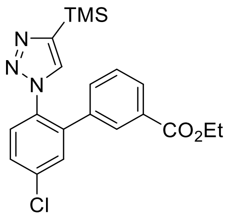

Following **TP3** (5-chloro-2-(4-(trimethylsilyl)-1*H*-1,2,3-triazol-1-yl)phenyl)magnesium reagent (**3a**, 0.405 mmol, 1.00 equiv) was coupled with ethyl 3-bromobenzoate (0.850 mmol, 195 mg, 2.10 equiv).

Isolated yield: 122 mg, 0.305 mmol, 75%, pale yellow solid.

Purification: pentane:ethyl acetate = 95 :5.

m.p.: 97.0 – 98.8 °C.

^{1}H-NMR (400 MHz, $CDCl_3$, ppm): *δ* = 7.99 (dt, *J* = 7.8, 1.4 Hz, 1H), 7.81 (td, *J* = 1.8, 0.5 Hz, 1H), 7.60 (d, *J* = 8.4 Hz, 1H), 7.55 (d, *J* = 2.2 Hz, 1H), 7.52 (dd, *J* = 8.4, 2.3 Hz, 1H), 7.30 (td, *J* = 7.8, 0.6 Hz, 1H), 7.11 (s, 1H), 7.10 – 7.07 (m, 1H), 4.36 (q, *J* = 7.1 Hz, 2H), 1.38 (t, *J* = 7.1 Hz, 3H), 0.20 (s, 9H).

^{13}C-NMR (100 MHz, $CDCl_3$, ppm): *δ* = 165.9, 146.8, 137.9, 136.6, 135.8, 133.8, 132.7, 131.3, 131.1, 130.9, 129.6, 129.1, 128.8, 128.1, 61.4, 14.4, −1.2.

FT-IR (ATR, cm^{-1}): $\tilde{\nu}$ = 2956, 1715, 1500, 1484, 1490, 1366, 1296, 1285, 1266, 1249, 1236, 1200, 1172, 1148, 1133, 1124, 1109, 1096, 1049, 1034, 996, 982, 927, 903, 871, 838, 808, 777, 758, 735, 708, 673, 659.

MS (EI, 70 eV): *m/z* (%) = 371 (10), 356 (12), 254 (11), 190 (16), 103 (100), 75 (63), 73 (28), 59 (12).

HR-MS (EI, 70 eV): [$C_{20}H_{22}ClN_3O_2Si$], calcd.: 399.1170; found: 399.1120.

1-(4-Fluoro-2-(naphthalen-1-yl)phenyl)-4-(trimethylsilyl)-1*H*-1,2,3-triazole (1g)

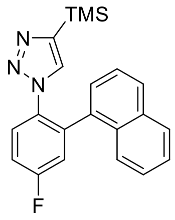

Following **TP3** (5-fluoro-2-(4-(trimethylsilyl)-1*H*-1,2,3-triazol-1-yl)phenyl)magnesium reagent (**3b**, 0.430 mmol, 1.00 equiv) was coupled with 1-bromonaphthalene (0.850 mmol, 176 mg, 1.98 equiv).

Isolated yield: 115 mg, 0.318 mmol, 74%, pale yellow oil.

Purification: pentane:ethyl acetate = 9:1.

^{1}H-NMR (400 MHz, $CDCl_3$, ppm): *δ* = 7.92 – 7.85 (m, 3H), 7.52 – 7.44 (m, 3H), 7.41 – 7.28 (m, 4H), 6.73 (s, 1H), 0.00 (s, 9H).

^{13}C-NMR (100 MHz, $CDCl_3$, ppm): *δ* = 162.4 (d, *J* = 250.6 Hz), 145.8, 137.1 (d, *J* = 8.6 Hz), 134.2 (d, *J* = 1.4 Hz), 133.3, 132.7 (d, *J* = 3.1 Hz), 131.0, 130.4, 129.1, 128.3, 127.6 (d, *J* = 9.0 Hz), 127.5, 127.0, 126.4, 125.3, 124.5, 118.7 (d, *J* = 23.0 Hz), 116.1 (d, *J* = 22.7 Hz), −1.5.

^{19}F NMR (377 MHz, $CDCl_3$, ppm): δ = −111.63 (m).

FT-IR (ATR, cm^{-1}): $\tilde{\nu}$ = 2956, 1614, 1585, 1502, 1402, 1301, 1248, 1197, 1171, 1146, 1033, 1020, 983, 894, 878, 838, 798, 775, 757, 731, 709, 696, 688, 667.

MS (EI, 70 eV): *m/z* (%) = 334 (24), 333 (100), 332 (62), 320 (20), 319 (93), 318 (57), 318 (57), 318 (57), 318 (87), 318 (52), 318 (54), 318 (55), 317 (50), 302 (29), 288 (20), 258 (14), 240 (19), 220 (33).

HR-MS (EI, 70 eV): [$C_{21}H_{20}FN_3Si$], calcd.: 361.1411; found: 361.1398.

1-(5-Fluoro-4'-(trifluoromethoxy)-[1,1'-biphenyl]-2-yl)-4-(trimethylsilyl)-1*H*-1,2,3-triazole (1h)

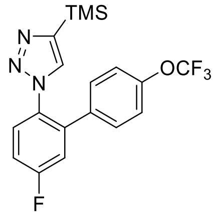

Following **TP3** (5-fluoro-2-(4-(trimethylsilyl)-1*H*-1,2,3-triazol-1-yl)phenyl)magnesium reagent (**3b**, 0.430 mmol, 1.00 equiv) was coupled with 1-bromo-4-(trifluoromethoxy)benzene (0.850 mmol, 205 mg, 1.98 equiv).

Isolated yield: 150 mg, 0.379 mmol, 88% colorless crystals.

Large scale-experiment

Following **TP3** (5-fluoro-2-(4-(trimethylsilyl)-1*H*-1,2,3-triazol-1-yl)phenyl)magnesium reagent (**3b**, 4.30 mmol, 1.00 equiv) was coupled with 1-bromo-4-(trifluoromethoxy)benzene (8.5 mmol, 2.05 g, 1.98 equiv).

Isolated yield: 1.29 g, 3.262 mmol, 76%, colorless crystals.

Purification: pentane:ethyl acetate = 9:1.

m.p.: 74.6 – 76.6 °C.

^{1}H-NMR (400 MHz, CDCl$_3$, ppm): δ = 7.60 (ddd, *J* = 8.3, 5.2, 0.9 Hz, 1H), 7.25 – 7.17 (m, 1H), 7.20 (d, *J* = 7.8 Hz, 1H), 7.12 – 7.07 (m, 2H), 7.06 (s, 1H), 7.05 – 7.01 (m, 2H), 0.19 (s, 9H).

^{13}C-NMR (100 MHz, CDCl$_3$, ppm): δ = 162.9 (d, *J* = 251.2 Hz), 149.4 (q, *J* = 1.9 Hz), 146.9, 138.4 (d, *J* = 8.6 Hz), 135.1 (d, *J* = 1.6 Hz), 131.5 (d, *J* = 3.2 Hz), 131.2, 130.0, 128.9 (d, *J* = 9.2 Hz), 121.3 (q, *J* = 1.0 Hz), 120.5 (q, *J* = 257.7 Hz), 117.6 (d, *J* = 23.5 Hz), 116.0 (d, *J* = 22.7 Hz), −1.3.

^{19}F NMR (377 MHz, CDCl$_3$, ppm): δ = −58.04 (t, *J* = 1.1 Hz), −109.19 – −111.70 (m).

FT-IR (ATR, cm^{-1}): $\tilde{\nu}$ = 1582, 1517, 1501, 1391, 1302, 1263, 1247, 1217, 1198, 1185, 1165, 1147, 1117, 1104, 1037, 1018, 999, 986, 958, 921, 889, 879, 843, 831, 815, 807, 767, 756, 677, 662.

MS (EI, 70 eV): *m/z* (%) = 367 (10), 366 (41), 365 (26), 313 (12), 287 (20), 286 (100), 270 (13), 259 (15), 257 (65), 224 (11), 222 (18), 190 (10), 77 (22), 73 (11).

HR-MS (EI, 70 eV): [$C_{18}H_{17}F_4N_3OSi$], calcd.: 395.1077; found: 395.1074.

1-(5-Fluoro-4'-(pentafluoro-λ^6-sulfaneyl)-[1,1'-biphenyl]-2-yl)-4-(trimethylsilyl)-1*H*-1,2,3-triazole (1i)

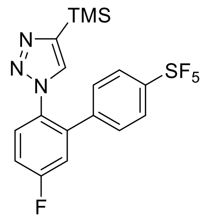

Following **TP3** (5-fluoro-2-(4-(trimethylsilyl)-1*H*-1,2,3-triazol-1-yl)phenyl)magnesium reagent (**3b**, 0.430 mmol, 1.00 equiv) was coupled with (4-bromophenyl)pentafluoro- λ^6-sulfane (0.850 mmol, 241 mg, 1.98 equiv).

Isolated yield: 178 mg, 0.407 mmol, 95%, pale yellow crystals.

Purification: pentane:ethyl acetate = 9:1.

m.p.: 84.1 – 85.9 °C.

^{1}H-NMR (400 MHz, CDCl$_3$, ppm): δ = 7.65 (ddd, *J* = 8.3, 2.3, 1.0 Hz, 1H), 7.59 – 7.52 (m, 1H), 7.42 – 7.34 (m, 1H), 7.30 (t, *J* = 1.9 Hz, 1H), 7.26 (s, 1H), 7.26 – 7.20 (m, 2H), 7.22 (d, *J* = 8.3 Hz, 2H), 0.19 (s, 9H).

^{13}C-NMR (100 MHz, CDCl$_3$, ppm): *δ* = 163.0 (d, *J* = 251.8 Hz), 154.0 (p, *J* = 17.8 Hz), 147.0, 137.96 (d, *J* = 8.6 Hz), 137.4 (d, *J* = 1.6 Hz), 131.6, 131.53 (d, *J* = 3.3 Hz), 131.0, 129.3, 129.1 (d, *J* = 9.1 Hz), 125.8 (m) 117.7 (d, *J* = 23.7 Hz), 116.4 (d, *J* = 22.7 Hz), −1.3.

^{19}F NMR (377 MHz, CDCl$_3$, ppm): δ = 85.25 – 81.34 (m), 62.70 (d, *J* = 150.3 Hz), −109.90 (td, *J* = 8.0, 5.1 Hz).

FT-IR (ATR, cm^{-1}): $\tilde{\nu}$ = 1511, 1486, 1475, 1387, 1281, 1249, 1200, 1192, 1146, 1112, 1035, 983, 923, 915, 905, 880, 875, 863, 826, 791, 761, 745, 761, 745, 692, 709, 6679, 668.

MS (EI, 70 eV): *m/z* (%) = 409 (50), 285 (72), 271 (18), 270 (100), 258 (20), 256 (27), 224 (44), 222 26), 77 (22).

HR-MS (EI, 70 eV): [$C_{17}H_{17}F_6N_3SSi$], calcd.: 437.0817; found: 437.0824.

1-(5-Methoxy-3'-(trifluoromethyl)-[1,1'-biphenyl]-2-yl)-4-(trimethylsilyl)-1*H*-1,2,3-triazole (1j)

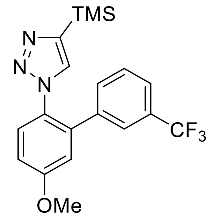

Following **TP3** (5-methoxy-2-(4-(trimethylsilyl)-1*H*-1,2,3-triazol-1-yl)phenyl)magnesium reagent (**3e**, 0.385 mmol, 1.00 equiv) was coupled with 1-bromo-3-(trifluoromethyl)benzene (0.850 mmol, 191 mg, 2.21 equiv).

Isolated yield: 106 mg, 0.271 mmol, 70%, colorless crystals.

Purification: pentane:ethyl acetate = 9:1 to 85:15.

m.p.:.98.8 – 100.2 °C

^{1}H-NMR (400 MHz, CDCl$_3$, ppm): *δ* = 7.31 (d, *J* = 8.6 Hz, 2H), 7.20 – 7.13 (m, 1H), 7.07 – 7.01 (m, 2H), 6.95 (s, 1H), 6.87 – 6.79 (m, 2H), 3.70 (s, 3H), 0.00 (s, 9H).

^{13}C-NMR (100 MHz, CDCl$_3$, ppm): *δ* = 160.5, 146.5, 138.3, 137.5, 131.9 (d, *J* = 1.5 Hz), 131.3, 131.0 (q, *J* = 32.5 Hz), 129.1, 128.4, 128.3, 125.3 – 125.1 (m), 124.7 (q, *J* = 3.8 Hz), 123.8 (q, *J* = 272.9 Hz), 116.0, 114.2, 56.0, −1.22.

^{19}F NMR (377 MHz, CDCl$_3$, ppm): δ = −62.74 (s).

FT-IR (ATR, cm^{-1}): $\tilde{\nu}$ = 1606, 1576, 1513, 1486, 1455, 1442, 1332, 1296, 1275, 1249, 1214, 4201, 1164, 1123, 10075, 1035, 996, 983, 909, 878, 839, 804, 788, 759, 732, 715, 702, 658, 676.

MS (EI, 70 eV): *m/z* (%) = 364 (15), 363 (61), 333 (25), 253 (12), 252 (70), 237 (12), 209 (58), 45 (16), 42 (100).

HR-MS (EI, 70 eV): [$C_{19}H_{20}F_3N_3OSi$], calcd.: 391.1328; found: 391.1320.

1-(5-Chloro-2'-fluoro-[1,1'-biphenyl]-2-yl)-4-(trimethylsilyl)-1*H*-1,2,3-triazole (1k)

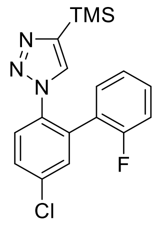

Following **TP3** (5-chloro-2-(4-(trimethylsilyl)-1*H*-1,2,3-triazol-1-yl)phenyl)magnesium reagent (**3a**, 0.405 mmol, 1.00 equiv) was coupled with 1-bromo-2-fluorobenzene (0.850 mmol, 149 mg, 2.10 equiv).

Isolated yield: 100 mg, 0.289 mmol, 71%, yellow crystals.

Purification: pentane:ethyl acetate = 9:1.

m.p.: 111.0 – 113.2 °C.

^{1}H-NMR (400 MHz, $CDCl_3$, ppm): *δ* = 7.64 (dd, J = 8.2, 0.8 Hz, 1H), 7.56 – 7.48 (m, 2H), 7.32 (dddd, J = 8.2, 7.3, 5.2, 2.1 Hz, 1H), 7.17 (s, 1H), 7.12 – 6.98 (m, 3H), 0.20 (s, 9H).

^{13}C-NMR (100 MHz, $CDCl_3$, ppm): *δ* = 159.5 (d, *J* = 248.2 Hz), 146.5, 135.2, 134.6, 132.4, 131.6 (d, *J* = 1.6 Hz), 131.0 (d, *J* = 2.6 Hz), 130.8 (d, *J* = 8.1 Hz), 130.3, 129.5, 127.4, 124.6 (d, *J* = 3.7 Hz), 124.0 (d, *J* = 15.4 Hz), 115.9 (d, *J* = 21.8 Hz), −1.2.

^{19}F NMR (377 MHz, $CDCl_3$, ppm): δ = −115.51 (ddd, *J* = 9.9, 7.0, 5.3 Hz).

FT-IR (ATR, cm^{-1}): $\tilde{\nu}$ = 1507, 1490, 1454, 1447, 1399, 1264, 1249, 1207, 1196, 1154, 1130, 1108, 1041, 984, 8887, 835, 827, 786, 753, 743, 704, 694, 657.

MS (EI, 70 eV): *m/z* (%) = 319 (14), 317 (43), 267 (14), 191 (15), 190 (100), 170 (24), 77 (37), 77 (37), 77 (56), 77 (56), 77 (56), 73 (18).

HR-MS (EI, 70 eV): [M+H$^+$] = [$C_{17}H_{18}ClFN_3Si^+$], calcd.: 346.0937; found: 346.0955.

3-(5-Chloro-2-(4-(trimethylsilyl)-1*H*-1,2,3-triazol-1-yl)phenyl)pyridine (1l)

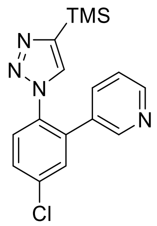

Following **TP3** (5-chloro-2-(4-(trimethylsilyl)-1*H*-1,2,3-triazol-1-yl)phenyl)magnesium reagent (**3a**, 0.405 mmol, 1.00 equiv) was coupled with 3-bromopyridine (0.850 mmol, 134 mg, 2.10 equiv).

Isolated yield: 112 mg, 0.341 mmol, 84%, colorless crystals.

Purification: pentane:ethyl acetate = 7:3.

m.p.: 129.5 – 131.3 °C.

^{1}H-NMR (400 MHz, $CDCl_3$, ppm): *δ* = 8.55 (dd, *J* = 4.8, 1.7 Hz, 1H), 8.44 (dd, *J* = 2.4, 0.9 Hz, 1H), 7.58 (dd, *J* = 8.2, 0.8 Hz, 1H), 7.56 (d, *J* = 2.2 Hz, 1H), 7.53 (dd, *J* = 2.3, 0.9 Hz, 1H), 7.23 (ddd, *J* = 7.9, 2.3, 1.7 Hz, 1H), 7.18 (s, 1H), 7.15 (ddd, *J* = 8.0, 4.8, 0.9 Hz, 1H), 0.22 (s, 9H).

^{13}C-NMR (100 MHz, $CDCl_3$, ppm): *δ* = 149.7, 149.0, 147.2, 136.1, 135.8, 135.6, 134.1, 132.3, 131.0, 130.9, 129.6, 128.4, 123.3, −1.1.

FT-IR (ATR, cm^{-1}): $\tilde{\nu}$ = 1501, 1473, 1411, 1250, 1201, 1154, 1125, 1097, 1042, 1014, 979, 911, 832, 814, 757, 741, 714, 741, 668.

MS (EI, 70 eV): *m/z* (%) = 300 (14), 287 (31), 286 (57), 285 (100), 284 (20), 284 (20), 284 (20), 284 (48), 284 (49), 255 (11), 73 (15).

HR-MS (EI, 70 eV): [M+H$^+$] = [$C_{16}H_{18}ClN_4Si^+$], calcd.: 329.0984; found: 329.0985.

3-(5-Methyl-2-(4-(trimethylsilyl)-1*H*-1,2,3-triazol-1-yl)phenyl)pyridine (1m)

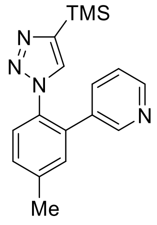

Following **TP3** 1-(*p*ara-tolyl)-4-(trimethylsilyl)-1*H*-1,2,3-triazole (**3d**, 0.340 mmol, 1.00 equiv) was coupled with 3-bromopyridine (0.850 mmol, 134 mg, 2.50 equiv).

Isolated yield: 72 mg, 0.233 mmol, 69%, yellow solid.

Purification: pentane:ethyl acetate = 8:2.

m.p.: 72.2 - 74.0.

^{1}H-NMR (400 MHz, $CDCl_3$, ppm): *δ* = 8.29 (dd, *J* = 4.8, 1.7 Hz, 1H), 8.24 (dd, *J* = 2.4, 0.9 Hz, 1H), 7.28 (d, *J* = 8.0 Hz, 1H), 7.14 (ddd, *J* = 8.1, 2.0, 0.9 Hz, 1H), 7.10 (d, *J* = 1.9 Hz, 1H), 7.01 – 6.95 (m, 2H), 6.89 (ddd, *J* = 8.0, 4.8, 0.9 Hz, 1H), 2.27 (s, 3H), 0.00 (s, 9H).

^{13}C-NMR (100 MHz, $CDCl_3$, ppm): *δ* = 149.2, 149.1, 146.7, 140.5, 135.9, 133.8, 133.6, 133.1, 131.4, 131.2, 130.0, 126.9, 123.1, 21.4, −1.1.

FT-IR (ATR, cm^{-1}): $\tilde{\nu}$ = 2956, 1512, 1409, 1248, 1205, 1156, 1134, 1039, 102, 997, 981, 832, 816, 755, 714, 668.

MS (EI, 70 eV): *m/z* (%) = 280 (18), 266 (16), 365 (72), 264 (100), 235 (10).

HR-MS (EI, 70 eV): [$C_{17}H_{20}N_4Si$], calcd.: 308.1457; found: 308.1451.

(5-Methyl-2-(4-(trimethylsilyl)-1*H*-1,2,3-triazol-1-yl)phenyl)magnesium reagent (1n)

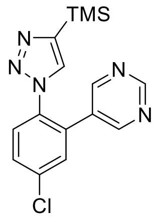

Following **TP3** (5-chloro-2-(4-(trimethylsilyl)-1*H*-1,2,3-triazol-1-yl)phenyl)magnesium reagent (**3a**, 0.405 mmol, 1.00 equiv) was coupled with 5-bromopyrimidine (0.850 mmol, 135 mg, 2.10 equiv).

Isolated yield: 83 mg, 0.252 mmol, 62%, yellow crystals.

Purification: pentane:ethyl acetate = 8:2.

m.p.: 125.8 – 127.4 °C.

^{1}H-NMR (400 MHz, $CDCl_3$, ppm): δ = 9.15 (s, 1H), 8.44 (s, 2H), 7.60 (dd, *J* = 8.5, 2.2 Hz, 1H), 7.55 (d, *J* = 8.5 Hz, 1H), 7.53 (d, *J* = 2.2 Hz, 1H), 7.38 (s, 1H), 0.27 (s, 9H).

^{13}C-NMR (100 MHz, $CDCl_3$, ppm): δ = 158.4, 155.9, 147.7, 136.5, 134.0, 132.5, 131.0, 130.7, 130.7, 130.3, 128.5, −1.1.

FT-IR (ATR, cm^{-1}): $\tilde{\nu}$ = 3103, 1548, 1507, 1218, 1404, 1386, 1247, 1205, 1185, 1157, 1115, 1037, 1018, 986, 979, 953, 890, 843, 822, 759, 737, 728, 737, 667.

MS (EI, 70 eV): *m/z* (%) = 301 (13), 288 (32), 287 (47), 286 (100), 285 (97), 259 (16), 73 (19).

HR-MS (EI, 70 eV): [$C_{15}H_{16}ClN_5Si$], calcd.: 329.0863; found: 329.0858

5-(2-Methoxy-6-(4-(trimethylsilyl)-1*H*-1,2,3-triazol-1-yl)phenyl)-1-methyl-1*H*-indole (1o)

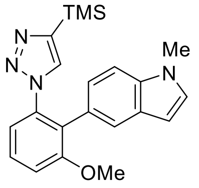

Following **TP3** (2-methoxy-6-(4-(trimethylsilyl)-1*H*-1,2,3-triazol-1-yl)phenyl)magnesium (**3f**, 0.350 mmol, 1.00 equiv) was coupled with 5-bromo-1-methyl-1*H*-indole (0.850 mmol, 179 mg, 2.43 equiv).

Isolated yield: 111 mg, 0.295 mmol, 84%, pale yellow oil.

Purification: pentane:ethyl acetate = 8:2.

^{1}H-NMR (400 MHz, $CDCl_3$, ppm): δ = 7.45 (t, *J* = 8.1 Hz, 1H), 7.34 (dd, *J* = 8.1, 1.1 Hz, 1H), 7.27 (dd, *J* = 1.7, 0.7 Hz, 1H), 7.19 (dt, *J* = 8.4, 0.8 Hz, 1H), 7.10 (dd, *J* = 8.4, 1.1 Hz, 1H), 7.00 (d, *J* = 3.1 Hz, 1H), 6.87 (dd, *J* = 8.5, 1.6 Hz, 1H), 6.82 (s, 1H), 6.36 (dd, *J* = 3.2, 0.8 Hz, 1H), 3.81 (s, 3H), 3.75 (s, 3H), 0.00 (s, 9H).

^{13}C-NMR (100 MHz, $CDCl_3$, ppm): δ = 157.7, 145.4, 137.4, 136.2, 131.3, 129.3, 128.6, 128.6, 127.4, 123.8, 123.4, 122.5, 118.2, 111.7, 109.1, 101.4, 56.3, 32.9, −1.3.

FT-IR (ATR, cm^{-1}): $\tilde{\nu}$ = 2953, 1598, 1581, 1513, 1488, 1473, 1438, 1422, 1331, 1300, 1246, 4210, 1181, 1092, 1037, 1014, 989, 839, 803, 792, 778, 760, 735, 719, 697, 667.

MS (EI, 70 eV): *m/z* (%) = 348 (25), 347 (34), 334 (25), 333 (100), 319 (11), 318 (42), 301 (7), 290 (6), 73 (7).

HR-MS (EI, 70 eV): [$C_{21}H_{24}N_4OSi$], calcd.: 376.1719; found: 376.1713.

1-(2-(Benzo[*b*]thiophen-5-yl)-4-methoxyphenyl)-4-(trimethylsilyl)-1*H*-1,2,3-triazol (1p)

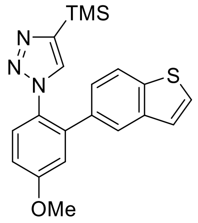

Following **TP3** (5-methoxy-2-(4-(trimethylsilyl)-1*H*-1,2,3-triazol-1-yl)phenyl)magnesium reagent (**3e**, 0.385 mmol, 1.00 equiv) was coupled with 5-bromobenzo[*b*]thiophene (0.850 mmol, 181 mg, 2.21 equiv).

Isolated yield: 130 mg, 0.343 mmol, 89%, pale yellow oil.

Purification: pentane:ethyl acetate = 9:1.

^{1}H-NMR (400 MHz, $CDCl_3$, ppm): δ = 7.73 (dt, *J* = 8.4, 0.7 Hz, 1H), 7.56 (d, *J* = 8.7 Hz, 1H), 7.48 (dd, *J* = 1.6, 0.7 Hz, 1H), 7.44 (d, *J* = 5.4 Hz, 1H), 7.23 (dd, *J* = 5.5, 0.8 Hz, 1H), 7.09 (d, *J* = 2.8 Hz, 1H), 7.05 (s, 1H), 7.04 – 6.94 (m, 2H), 3.91 (s, 3H), 0.12 (s, 9H).

^{13}C-NMR (100 MHz, $CDCl_3$, ppm): δ = 160.4, 146.2, 139.8, 139.4, 138.8, 133.6, 131.6, 128.7, 128.1, 127.4, 124.6, 124.0, 123.5, 122.6, 116.2, 113.7, 55.9, −1.2.

FT-IR (ATR, cm^{-1}): $\tilde{\nu}$ = 2953, 1603, 1575, 1544, 1513, 1500, 1463, 1436, 1412, 1328, 1291, 1247, 1220, 1199, 1183, 1168, 1154, 1128, 1090, 1055, 1037, 995, 983, 837, 814, 755, 739, 702, 667.

MS (EI, 70 eV): *m/z* (%) = 358 (25), 357 (80), 355 (100), 340 (16), 339 (63), 337 (16), 320 (17), 306 (20), 306 (20), 305 (80), 246 (18), 73 (12).

HR-MS (EI, 70 eV): [$C_{20}H_{21}N_3OSSi$], calcd.: 379.1175; found: 379.1170.

1-(2-(Thiophen-3-yl)phenyl)-4-(trimethylsilyl)-1*H*-1,2,3-triazole (1q)

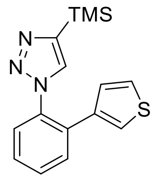

Following **TP3** (2-(4-(trimethylsilyl)-1*H*-1,2,3-triazol-1-yl)phenyl)magnesium reagent (**3c**, 0.360 mmol, 1.00 equiv) was coupled with 3-bromothiophene (0.850 mmol, 139 mg, 2.36 equiv).

Isolated yield: 70 mg, 0.234 mmol, 65%, colorless oil.

Purification: pentane:ethyl acetate = 9:1.

^{1}H-NMR (400 MHz, CDCl$_3$, ppm): *δ* = 7.60 – 7.56 (m, 2H), 7.50 (m, 2H), 7.24 (s, 1H), 7.20 (dd, *J* = 5.0, 3.0 Hz, 1H), 6.96 (dd, *J* = 3.0, 1.3 Hz, 1H), 6.62 (dd, *J* = 5.0, 1.3 Hz, 1H), 0.26 (s, 9H).

^{13}C-NMR (100 MHz, CDCl$_3$, ppm): *δ* = 146.6, 137.6, 135.1, 132.5, 131.2, 130.5, 129.9, 128.5, 127.5, 127.0, 126.0, 123.6, −1.1.

FT-IR (ATR, cm^{-1}): $\tilde{\nu}$ = 2956, 1495, 1364, 1248, 1200, 1150, 1112, 1084, 1053, 1036, 983, 838, 792, 756, 740, 704, 664.

MS (EI, 70 eV): *m/z* (%) = 271 (24), 270 (76), 258 89), 257 (20), 256 (100), 255 (26), 239 (21), 225 (15), 166 (13), 115 (9).

HR-MS (EI, 70 eV): [$C_{15}H_{17}N_3SSi$], calcd.: 299.0912; found: 299.2912.

1-(4-Chloro-2-(thiophen-3-yl)phenyl)-4-(trimethylsilyl)-1*H*-1,2,3-triazole (1r)

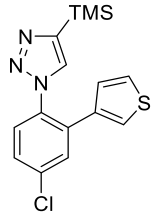

Following **TP3** (5-chloro-2-(4-(trimethylsilyl)-1*H*-1,2,3-triazol-1-yl)phenyl)magnesium reagent (**3a**, 0.405 mmol, 1.00 equiv) was coupled with 3-bromothiophene (0.850 mmol, 139 mg, 2.10 equiv).

Isolated yield: 130 mg, 0.389 mmol, 96%, pale yellow crystals

m.p.: 57.5 – 59.7 °C.

Purification: pentane:ethyl acetate = 95:5.

^{1}H-NMR (400 MHz, CDCl$_3$, ppm): *δ* = 7.56 (d, *J* = 2.3 Hz, 1H), 7.52 (d, *J* = 8.4 Hz, 1H), 7.43 (dd, *J* = 8.5, 2.4 Hz, 1H), 7.21 (m, 2H), 6.97 (dd, *J* = 2.9, 1.3 Hz, 1H), 6.59 (dd, *J* = 5.0, 1.4 Hz, 1H), 0.25 (s, 9H).

^{13}C-NMR (100 MHz, CDCl$_3$, ppm): δ = 146.8, 136.2, 135.6, 133.9, 133.5, 131.1, 130.3, 128.4, 128.2, 127.2, 126.5, 124.3, −1.1.

FT-IR (ATR, cm^{-1}): $\tilde{\nu}$ = 3108, 2956, 1533, 1497, 1471, 1247, 1204, 1152, 1126, 1091, 1082, 1041, 1000, 979, 908, 890, 830, 787, 754, 736, 708, 694, 673.

MS (EI, 70 eV): *m/z* (%) = 307 (12), 306 (41), 305 (31), 304 (99), 292 (35), 291 (34), 290 (100), 289 (13), 261 (11), 256 (14), 255 (42), 240 (29), 208 (11), 73 (23), 44 (11).

HR-MS (EI, 70 eV): [$C_{15}H_{16}ClN_3SSi$], calcd.: 333.0523; found: 333.0529.

Ethyl 5-(5-chloro-2-(4-(trimethylsilyl)-1H-1,2,3-triazol-1-yl)phenyl)furan-2-carboxylate (1s)

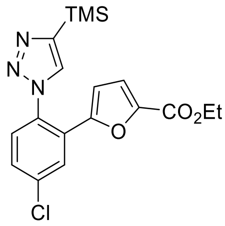

Following **TP3** (5-chloro-2-(4-(trimethylsilyl)-1*H*-1,2,3-triazol-1-yl)phenyl)magnesium reagent (**3b**, 0.405 mmol, 1.00 equiv) was coupled with ethyl 5-bromofuran-2-carboxylate (0.850 mmol, 186 mg, 2.07 equiv).

Isolated yield: 149 mg, 0.381 mmol, 94%, orange crystals.

Purification: pentane:ethyl acetate = 9:1.

m.p.: 136.6 – 138.4 °C.

^{1}H-NMR (400 MHz, CDCl$_3$, ppm): δ = 8.05 (d, *J* = 2.3 Hz, 1H), 7.60 (s, 1H), 7.46 (dd, *J* = 8.4, 2.3 Hz, 1H), 7.36 (d, *J* = 8.4 Hz, 1H), 7.00 (d, *J* = 3.7 Hz, 1H), 5.26 (d, *J* = 3.7 Hz, 1H), 4.36 (q, *J* = 7.1 Hz, 2H), 1.37 (t, *J* = 7.1 Hz, 3H), 0.36 (s, 9H).

^{13}C-NMR (100 MHz, CDCl$_3$, ppm): δ = 158.4, 150.6, 147.7, 145.0, 136.9, 132.2, 131.0, 129.4, 129.4, 128.4, 128.2, 119.5, 111.5, 61.4, 14.5, −1.0.

FT-IR (ATR, cm^{-1}): $\tilde{\nu}$ =1726, 1499, 1468, 1386, 1297, 1148, 1215, 1199, 1158, 1149, 1124, 1114, 1098, 1058, 1035, 1024, 996, 968, 980, 935, 883, 826, 834, 819, 784, 762, 710, 678, 668.

MS (EI, 70 eV): *m/z* (%) = 361 (17), 348 (27), 332 (77), 318 (14), 303 (38), 288 (25), 258 (20), 253 (15), 103 (45), 75 (100), 73 (83).

HR-MS (EI, 70 eV): [$C_{18}H_{20}ClN_3O_3Si$], calcd.: 389.0962; found: 389.0943.

1-(2-(Benzo[*d*][1,3]dioxol-5-yl)-4-chlorophenyl)-3,5-dimethyl-1*H*-pyrazole (6a)

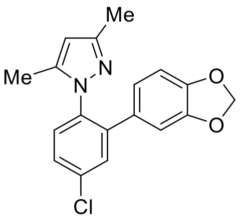

Following **TP3** (5-chloro-2-(3,5-dimethyl-1*H*-pyrazol-1-yl)phenyl)magnesium reagent (**5a**, 0.410 mmol, 1.00 equiv) was coupled with 5-bromobenzo[*d*][1,3]dioxole (0.850 mmol, 171 mg, 2.07 equiv).

Isolated yield: 127 mg, 0.389 mmol, 95%, pale yellow oil.

Purification: pentane:ethyl acetate = 95:5.

^{1}H-NMR (400 MHz, $CDCl_3$, ppm): δ = 7.45 (dd, *J* = 1.8, 1.0 Hz, 1H), 7.40 – 7.33 (m, 2H), 6.72 (d, *J* = 8.1 Hz, 1H), 6.59 (dd, *J* = 8.1, 1.8 Hz, 1H), 6.50 (d, *J* = 1.8 Hz, 1H), 5.94 (s, 2H), 5.79 (s, 1H), 2.28 (s, 3H), 1.66 (s, 3H).

^{13}C-NMR (100 MHz, $CDCl_3$, ppm): δ = 149.2, 147.9, 147.5, 140.8, 140.4, 136.0, 134.8, 131.2, 130.4, 130.2, 128.0, 122.4, 108.8, 108.5, 106.0, 101.3, 13.7, 11.3.

FT-IR (ATR, cm^{-1}): $\tilde{\nu}$ = 2898, 1608, 1594, 1553, 1499, 1483, 1434, 1401, 1364, 1335, 1277, 1251, 1238, 1220, 1149, 1134, 1105, 1094, 1037, 1023, 1012, 973, 934, 906, 862, 824, 814, 797, 759, 738, 718, 689, 667.

MS (EI, 70 eV): *m/z* (%) = 328 (14), 327 (13), 326 (43), 325 (16), 313 (29), 312 (16), 311 (100), 138 (7).

HR-MS (EI, 70 eV): [$C_{18}H_{15}ClN_2O_2$], calcd.: 326.0822; found: 326.0822.

3-(5-Chloro-2-(3,5-dimethyl-1*H*-pyrazol-1-yl)phenyl)pyridine (6b)

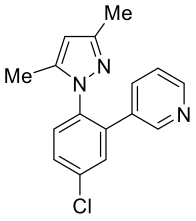

Following **TP3** (5-chloro-2-(3,5-dimethyl-1*H*-pyrazol-1-yl)phenyl)magnesium reagent (**5a**, 0.410 mmol, 1.00 equiv) was coupled with 3-bromopyridine (0.850 mmol, 134 mg, 2.07 equiv).

Isolated yield: 79 mg, 0.277 mmol, 68%, pale yellow oil.

Purification: pentane:ethyl acetate = 8:2 to 7:3.

^{1}H-NMR (400 MHz, $CDCl_3$, ppm): δ = 8.52 (dd, *J* = 4.8, 1.7 Hz, 1H), 8.39 (dd, *J* = 2.4, 0.9 Hz, 1H), 7.51 (d, *J* = 2.3 Hz, 1H), 7.48 (dd, *J* = 8.4, 2.3 Hz, 1H), 7.43 (d, *J* = 8.3 Hz, 1H), 7.31 (ddd, *J* = 8.0, 2.4, 1.7 Hz, 1H), 7.18 (ddd, *J* = 8.0, 4.8, 0.9 Hz, 1H), 5.79 (s, 1H), 2.25 (s, 3H), 1.75 – 1.60 (m, 3H).

^{13}C-NMR (100 MHz, CDCl$_3$, ppm): δ = 149.5, 149.2, 149.1, 140.6, 137.6, 136.4, 135.8, 135.3, 133.2, 130.6, 130.2, 129.3, 123.4, 106.4, 13.6, 11.3.

FT-IR (ATR, cm^{-1}): $\tilde{\nu}$ = 1588, 1553, 1499, 1473, 1415, 1393, 1363, 1332, 1292, 1252, 1190, 1131, 1096, 1028, 1017, 1010, 972, 882, 821, 808, 781, 740, 712, 668, 653.

MS (EI, 70 eV): *m/z* (%) = 270 (30), 269 (16), 268 (100), 192 (9).

HR-MS (EI, 70 eV): [M-Me^{-}] = [$C_{15}H_{11}ClN_3^{+}$], calcd.: 268.0636; found:268.0633.

5'-Chloro-2'-(1*H*-pyrazol-1-yl)-[1,1'-biphenyl]-3-yl 4-methylbenzenesulfonate (6c)

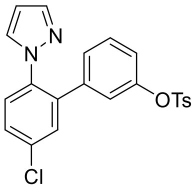

Following **TP3** (5-chloro-2-(1*H*-pyrazol-1-yl)phenyl)magnesium reagent (**5b**, 0.390 mmol, 1.00 equiv) was coupled with 3-bromophenyl 4-methylbenzenesulfonate (0.850 mmol, 278 mg, 2.18 equiv).

Isolated yield: 148 mg, 0.348 mmol, 89%, colorless oil.

Purification: pentane:ethyl acetate = 85:15.

^{1}H-NMR (400 MHz, CDCl$_3$, ppm): δ = 7.66 – 7.56 (m, 2H), 7.53 (d, *J* = 1.8 Hz, 1H), 7.45 (d, *J* = 8.5 Hz, 1H), 7.36 (dd, *J* = 8.5, 2.4 Hz, 1H), 7.27 (d, J = 8.1 Hz, 2H), 7.15 – 7.06 (m, 2H), 6.96 – 6.86 (m, 2H), 6.77 (dt, *J* = 7.8, 1.3 Hz, 1H), 6.68 (t, *J* = 2.0 Hz, 1H), 6.13 (t, *J* = 2.1 Hz, 1H), 2.40 (s, 3H),

^{13}C-NMR (100 MHz, CDCl$_3$, ppm): δ = 149.8, 145.8, 140.9, 139.0, 137.1, 136.6, 134.0, 132.2, 131.2, 130.7, 130.0, 129.9, 129.0, 128.7, 128.0, 127.2, 122.6, 122.2, 107.1, 21.9.

FT-IR (ATR, cm^{-1}): $\tilde{\nu}$ = 1596, 1580, 1566, 1517, 1476, 1430, 1371, 1329, 1307, 1293, 1263, 1211, 1192, 1179, 1147, 1110, 1091, 1042, 1019, 1002, 936, 916, 893, 877, 805, 793, 746, 695, 659.

MS (EI, 70 eV): *m/z* (%) = 271 (32), 270 (17), 269 (100), 90 (16), 61 (9), 43 (52), 42 (18).

HR-MS (EI, 70 eV): [$C_{22}H_{17}ClN_2O3S$], calcd.: 424.0648; found: 424.0626.

5'-Chloro-3-fluoro-2'-(1*H*-pyrazol-1-yl)-[1,1'-biphenyl]-4-carbonitrile (6d)

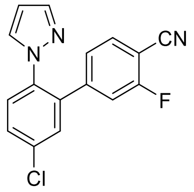

Following **TP3** (5-chloro-2-(1*H*-pyrazol-1-yl)phenyl)magnesium reagent (**5b**, 0.390 mmol, 1.00 equiv) was coupled with 4-bromo-2-fluorobenzonitrile (0.850 mmol, 170 mg, 2.18 equiv).

Isolated yield: 102 mg, 0.342 mmol, 88%, yellow crystals.

Purification: pentane:ethyl acetate = 85:15.

m.p.: 128.8 – 130.4 °C.

^{1}H-NMR (400 MHz, CDCl$_3$, ppm): δ = 7.61 (d, J = 1.8 Hz, 1H), 7.57 – 7.49 (m, 3H), 7.45 (dd, J = 1.8, 1.0 Hz, 1H), 7.20 (d, J = 2.4 Hz, 1H), 6.98 – 6.90 (m, 2H), 6.31 (t, J = 2.1 Hz, 1H).

^{13}C-NMR (100 MHz, CDCl$_3$, ppm): δ = 163.0 (d, J = 259.9 Hz), 144.8 (d, J = 8.4 Hz), 141.3, 137.2, 135.7 (d, J = 1.9 Hz), 134.7, 133.6 (d, J = 0.8 Hz), 131.1, 130.5, 130.1, 128.4, 124.9 (d, J = 3.5 Hz), 116.5 (d, J = 20.7 Hz), 113.7, 107.7, 101.0 (d, J = 15.5 Hz).

^{19}F NMR (377 MHz, CDCl$_3$, ppm): δ = −105.54 (dd, J = 9.7, 6.6 Hz).

FT-IR (ATR, cm^{-1}): $\tilde{\nu}$ = 2232, 1622, 1575, 1558, 1550, 1486, 1421, 1392, 1325, 1300, 1259, 1203, 1191, 1185, 1121, 1111, 1095, 1048, 1041, 1022, 937, 913, 894, 848, 833, 779, 763, 749, 732, 719, 689.

MS (EI, 70 eV): *m/z* (%) = 298 (32), 297 (16), 296 (100), 261 (15), 234 (12), 233 (11), 195 (7).

HR-MS (EI, 70 eV): [M-H$^+$] = [$C_{16}H_8ClFN_3^-$], calcd.: 296.0396; found: 296.0384.

2-([1,1'-Biphenyl]-2-yl)-5-phenyl-1,3,4-oxadiazole (6e)

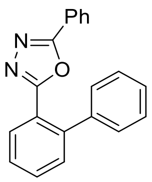

Following **TP3** (2-(5-phenyl-1,3,4-oxadiazol-2-yl)phenyl)magnesium reagent (**5c**, 0.380 mmol, 1.00 equiv) was coupled with bromobenzene (0.850 mmol, 133 mg, 2.24 equiv).

Isolated yield: 91 mg, 0.305 mmol, 80%, pale yellow oil.

Purification: pentane:ethyl acetate = 9:1.

^{1}H-NMR (400 MHz, CDCl$_3$, ppm): δ = 8.19 (dd, J = 7.8, 1.4 Hz, 1H), 7.63 – 7.57 (m, 3H), 7.56 – 7.42 (m, 3H), 7.42 – 7.34 (m, 5H), 7.34 – 7.29 (m, 2H).

^{13}C-NMR (100 MHz, CDCl$_3$, ppm): δ = 165.2, 164.7, 142.1, 140.9, 131.6, 131.5, 131.1, 130.2, 128.9, 128.8, 128.3, 127.9, 127.6, 126.7, 123.8, 122.8.

FT-IR (ATR, cm^{-1}): $\tilde{\nu}$ = 3057, 1607, 1598, 1571, 150, 1488, 1461, 1448, 1437, 1362, 1315, 1292, 1272, 1249, 1178, 1159, 1116, 1107, 1069, 1043, 1026, 1009, 988, 965, 924, 842, 778, 769, 758, 742, 715, 696, 669.

MS (EI, 70 eV): *m/z* (%) = 298 (21), 297 (100), 166 (28), 153 (5), 152 (18).

HR-MS (EI, 70 eV): [M-H$^+$] = [$C_{20}H_{13}N_2O^-$], calcd.: 297.1033; found: 297.1021.

Ethyl 2'-(5-phenyl-1,3,4-oxadiazol-2-yl)-[1,1'-biphenyl]-3-carboxylate (6f)

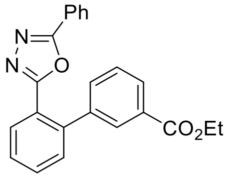

Following **TP3** (2-(5-phenyl-1,3,4-oxadiazol-2-yl)phenyl)magnesium reagent (**5c**, 0.380 mmol, 1.00 equiv) was coupled with ethyl 3-bromobenzoate (0.850 mmol, 195 mg, 2.24 equiv).

Isolated yield: 106 mg, 0.286 mmol, 75%, yellow solid.

Purification: pentane:ethyl acetate = 9:1.

m.p.: 121.9 – 123.7 °C.

^{1}H-NMR (400 MHz, $CDCl_3$, ppm): *δ* = 8.24 – 8.18 (m, 1H), 8.12 – 8.05 (m, 2H), 7.65 – 7.52 (m, 4H), 7.51 – 7.41 (m, 4H), 7.38 (ddt, *J* = 8.5, 6.8, 1.3 Hz, 2H), 4.35 (q, *J* = 7.1 Hz, 2H), 1.34 (t, *J* = 7.1 Hz, 3H).

^{13}C-NMR (100 MHz, $CDCl_3$, ppm): *δ* = 166.3, 164.7, 164.7, 141.0, 140.8, 133.3, 131.6, 131.5, 131.2, 130.6, 130.1, 129.9, 128.9, 128.8, 128.3, 126.6, 123.6, 122.7, 61.2, 14.3.

FT-IR (ATR, cm^{-1}): $\tilde{\nu}$ = 1709, 1597, 1582, 1543, 1496, 1489, 1474, 1448, 1414, 1393, 1367, 1311, 1301, 1277, 1245, 1168, 1138, 1107, 1072, 1049, 1024, 990, 964, 908, 882, 865, 851, 828, 786, 736, 748, 714, 702, 691, 656.

MS (EI, 70 eV): *m/z* (%) = 370 (29), 369 (100), 152 (7), 151 (7), 105 (19), 77 (12).

HR-MS (EI, 70 eV): [$C_{23}H_{17}N_2O_3$], calcd.: 369.1245; found: 369.1233.

4,4-Dimethyl-2-(4'-(pentafluoro-λ^6-sulfaneyl)-[1,1'-biphenyl]-2-yl)-4,5-dihydrooxazole (6g)

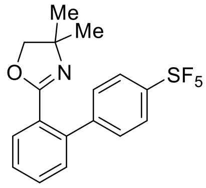

Following **TP3** (2-(4,4-dimethyl-4,5-dihydrooxazol-2-yl)phenyl)magnesium reagent (**5d**, 0.385 mmol, 1.00 equiv) was coupled with (4-bromophenyl)pentafluoro-λ^6-sulfane (0.850 mmol, 241 mg, 2.21 equiv).

Isolated yield: 90 mg, 0.238 mmol, 62%, pale yellow oil.

Purification: pentane:ethyl acetate = 8:2.

^{1}H-NMR (400 MHz, $CDCl_3$, ppm): *δ* = 7.81 – 7.72 (m, 3H), 7.52 (td, *J* = 7.6, 1.5 Hz, 1H), 7.48 – 7.40 (m, 3H), 7.34 (dd, *J* = 7.6, 1.3 Hz, 1H), 3.82 (s, 2H), 1.27 (s, 6H).

^{13}C-NMR (100 MHz, $CDCl_3$, ppm): *δ* = 163.0, 152.9 (p, *J* = 17.2 Hz), 144.9, 139.7, 130.8, 130.5, 130.2, 128.8, 128.2, 128.1, 125.7 (p, *J* = 4.7 Hz), 79.6, 67.9, 28.1.

^{19}F NMR (377 MHz, CDCl3, ppm): δ = 85.77 – 83.67 (m), 63.13 (d, *J* = 150.1 Hz).

FT-IR (ATR, cm^{-1}): $\tilde{\nu}$ = 2967, 1654, 1598, 1784, 1463, 1448, 1399, 1365, 1349, 1310, 1213, 1189, 1100, 1076, 1061, 1037, 963, 822, 770, 754, 730, 699, 683, 662.

MS (EI, 70 eV): *m/z* (%) = 376(100), 322 (22), 291 (75), 250 (23), 220 (29), 183 (60), 180 (23), 179 (19), 178 (45), 177 (48), 163 (25), 152 (31), 151 (72), 150 (36).

HR-MS (EI, 70 eV): [M-H$^+$] = [$C_{17}H_{15}F_5NO_2S^-$], calcd:. 376.0800; found: 376.0793.

4,4-Dimethyl-2-(2-(1-methyl-1*H*-indol-5-yl)phenyl)-4,5-dihydrooxazole (6h)

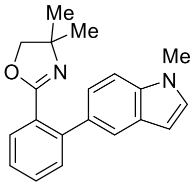

Following **TP3** (2-(4,4-dimethyl-4,5-dihydrooxazol-2-yl)phenyl)magnesium reagent (**5d**, 0.385 mmol, 1.00 equiv) was coupled with 5-bromo-1-methyl-1*H*-indole (0.850 mmol, 179 mg, 2.18 equiv).

Isolated yield: 107 mg, 0.352 mmol, 91%, orange crystals.

Purification: pentane:ethyl acetate = 8:2.

m.p.: 154.1 – 155.9 °C.

^{1}H-NMR (400 MHz, CDCl$_3$, ppm): δ = 7.72 (dt, *J* = 7.7, 1.1 Hz, 1H), 7.69 (dd, *J* = 1.7, 0.8 Hz, 1H), 7.51 – 7.44 (m, 2H), 7.37 – 7.32 (m, 2H), 7.30 (dd, *J* = 8.5, 1.7 Hz, 1H), 7.08 (d, *J* = 3.1 Hz, 1H), 6.49 (dd, *J* = 3.1, 0.8 Hz, 1H), 3.82 (s, 3H), 3.74 (s, 2H), 1.31 (s, 6H).

^{13}C-NMR (100 MHz, CDCl$_3$, ppm): δ = 164.6, 142.8, 136.2, 132.6, 130.7, 130.4, 130.3, 129.4, 128.5, 128.3, 126.4, 122.6, 120.7, 108.8, 101.3, 79.7, 67.4, 33.1, 28.2.

FT-IR (ATR, cm^{-1}): $\tilde{\nu}$ = 2968, 1658, 1615, 1509, 1470, 1436, 1212, 1365, 1334, 138, 1299, 1246, 1181, 1155, 1107, 1083, 1066, 1032, 963, 920, 871, 810, 774, 755, 718, 691, 655.

MS (EI, 70 eV): *m/z* (%) = 304 (20), 303 (100), 248 (61), 231 (19), 218 (36), 217 (13), 190 (13).

HR-MS (EI, 70 eV): [M-H$^+$] = [$C_{20}H_{29}N_2O^-$], calcd.: 303.1503; found: 303.1493.

1-(4-Chloro-2-iodophenyl)-4-(trimethylsilyl)-1*H*-1,2,3-triazole (7a)

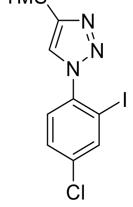

(5-Chloro-2-(4-(trimethylsilyl)-1*H*-1,2,3-triazol-1-yl)phenyl)magnesium reagent (**3a**, 0.405 mmol, 1.00 equiv) was cooled to 0 °C and quenched with an iodine solution (1.75 mmol, 4.32 equiv) in THF (2 mL). The reaction mixture

was stirred for 15 min and the remaining iodine was quenched with saturated aq. $Na_2S_2O_3$ solution (15 mL). The mixture was extracted with DCM (3 x 25 mL). The combined organic layers were dried over $MgSO_4$. The solvents were removed under reduced pressure and the crude product was subjected to column chromatography on silica yielding 1-(4-chloro-2-iodophenyl)-4-(trimethylsilyl)-1*H*-1,2,3-triazole.

Isolated yield: 150 mg, 0.397 mmol, 98%, white crystals.

Purification: pentane:ethyl acetate = 95:5.

m.p.: 111.0 – 113.2 °C

^{1}H-NMR (400 MHz, $CDCl_3$, ppm): δ = 7.98 (d, J = 2.2 Hz, 1H), 7.80 (s, 1H), 7.47 (dd, J = 8.4, 2.3 Hz, 1H), 7.35 (d, J = 8.4 Hz, 1H), 0.38 (s, 9H).

^{13}C-NMR (100 MHz, $CDCl_3$, ppm): δ = 146.8, 139.6, 139.1, 136.5, 130.9, 129.5, 128.4, 94.4, −1.00.

FT-IR (ATR, cm^{-1}): $\tilde{\nu}$ = 3076, 2957, 1574, 1495, 1473, 1436, 1401, 1372, 1250, 1204, 1160, 1101, 1047, 1040, 1005, 999, 985, 875, 835, 825, 773, 756, 710, 672, 697.

MS (EI, 70 eV): *m/z* (%) = 348 (12), 335 (29), 334 (60), 209 (32), 208 (19), 207 (100), 194 (18), 192 (55), 73 (19).

HR-MS (EI, 70 eV): [M+H$^+$] = [$C_{11}H_{14}N_3ClSi^+$], calcd.: 377.9685; found: 377.9669.

(5-Chloro-2-(4-(trimethylsilyl)-1*H*-1,2,3-triazol-1-yl)phenyl)(phenyl)methanol (7b)

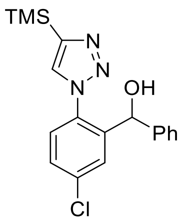

(5-Chloro-2-(4-(trimethylsilyl)-1*H*-1,2,3-triazol-1-yl)phenyl)magnesium reagent (**3a**, 0.405 mmol, 1.00 equiv) was cooled to 0 °C and benzaldehyde (0.10 mL, 1.00 mmol, 2.47 equiv) was added dropwise. The reaction was stirred for 1.5 h at this temperature and was then allowed to warm to room temperature. Saturated aq. NH_4Cl solution (5 mL) was added and the mixture was extracted with EtOAc (3 x 25 mL). The combined organic layers were dried over $MgSO_4$. The solvents were removed under reduced pressure and the crude product was subjected to column chromatography on silica yielding (5-chloro-2-(4-(trimethylsilyl)-1*H*-1,2,3-triazol-1-yl)phenyl)(phenyl)methanol.

Isolated yield: 125 mg, 0.349 mmol, 86%, pale yellow crystals.

Purification: pentane:ethyl acetate = 8:2.

m.p.: 103.8 – 105.7 °C.

^{1}H-NMR (400 MHz, $CDCl_3$, ppm): δ = 7.83 (d, J = 2.4 Hz, 1H), 7.35 (dd, J = 8.3, 2.4 Hz, 1H), 7.22 (s, 1H), 7.17 – 7.08 (m, 4H), 6.97 – 6.89 (m, 2H), 5.87 (d, J = 5.6 Hz, 1H), 4.80 (d, J = 5.7 Hz, 1H), 0.26 (s, 9H).

^{13}C-NMR (100 MHz, $CDCl_3$, ppm): δ = 146.7, 142.4, 141.8, 136.2, 133.4, 131.0, 129.2, 128.4, 128.3, 127.5, 127.26, 126.5, 71.2, −1.2.

FT-IR (ATR, cm^{-1}): $\tilde{\nu}$ = 3220, 1493, 1448, 1403, 1345, 1268, 1251, 1235, 1209, 1179, 1157, 1119, 1096, 1054, 1046, 1027, 1016, 986, 926, 906, 883, 836, 821, 772, 759, 736, 726, 700, 661.

MS (EI, 70 eV): *m/z* (%) = 314 (18), 312 (40), 254 (26), 252 (54), 241 (31), 239 (90), 237 (35), 235 (46), 203 (34), 76 (29), 75 (80), 73 (100).

HR-MS (EI, 70 eV): [$C_{18}H_{20}ClN_3OSi$], calcd.: 357.1064; found: 357.1065.

1-(4-Chloro-2-(methylthio)phenyl)-4-(trimethylsilyl)-1*H*-1,2,3-triazole (7c)

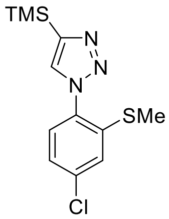

(5-Chloro-2-(4-(trimethylsilyl)-1*H*-1,2,3-triazol-1-yl)phenyl)magnesium reagent (**3a**, 0.405 mmol, 1.00 equiv) was cooled to 0 °C and MeSSO$_2$Me (1.00 mmol, 126 mg, 2.47 equiv) was added portionwise. The reaction was stirred for 1h at this temperature and was allowed to warm to room temperature. Saturated aq. NH_4Cl solution (5 mL) was added and the mixture was extracted with EtOAc (3 x 25 mL). The combined organic layers were dried over $MgSO_4$. The solvents were removed under reduced pressure and the crude product was subjected to column chromatography on silica yielding 1-(4-chloro-2-(methylthio)phenyl)-4-(trimethylsilyl)-1*H*-1,2,3-triazole.

Isolated yield: 90 mg, 0.302 mmol, 75%, colorless crystals.

Purification: pentane:ethyl acetate = 95:5.

m.p.: 84.0 – 85.8 °C.

^{1}H-NMR (400 MHz, $CDCl_3$, ppm): δ = 7.84 (s, 1H), 7.34 (d, *J* = 8.4 Hz, 1H), 7.31 (d, *J* = 2.2 Hz, 1H), 7.26 – 7.23 (m, 1H), 2.39 (s, 3H), 0.38 (s, 9H).

^{13}C-NMR (100 MHz, $CDCl_3$, ppm): δ = 146.6, 137.6, 136.2, 133.7, 130.9, 127.9, 126.3, 125.7, 15.7, −0.9.

FT-IR (ATR, cm^{-1}): $\tilde{\nu}$ = 3077, 1583, 1493, 1472, 1437, 1426, 1400, 1382, 1249, 1204, 1191, 1154, 1096, 1075, 7039, 1005, 987, 980, 955, 822, 796, 756, 711, 699, 680, 655.

MS (EI, 70 eV): *m/z* (%) = 253 (22), 240 (38), 239 (17), 238 (100), 223 (10), 73 (17).

HR-MS (EI, 70 eV): [$C_{12}H_{16}ClN_3SSi$], calcd.: 297.0523; found: 297.0513.

(5-Chloro-2-(4-(trimethylsilyl)-1*H*-1,2,3-triazol-1-yl)phenyl)(phenyl)methanone (7d)

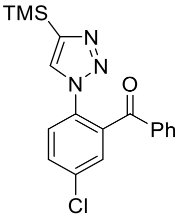

(5-Chloro-2-(4-(trimethylsilyl)-1*H*-1,2,3-triazol-1-yl)phenyl)magnesium reagent (**3a**, 0.405 mmol, 1.00 equiv) was cooled to -40 °C, CuCN·2•LiCl solution (1 M in THF, 1.20 mmol, 2.96 equiv) was added and the reaction mixture was for 30 min. Benzoyl chloride (0.12 mL, 1.00 mmol, 2.47 equiv) was added dropwise and the reaction mixture was allowed to come to room temperature for 16 h. Saturated aq. NH_4Cl solution (5 mL) was added and the mixture was extracted with EtOAc (3 x 25 mL). The combined organic layers were dried over $MgSO_4$. The solvents were removed under reduced pressure and the crude product was subjected to column chromatography on silica yielding (5-chloro-2-(4-(trimethylsilyl)-1*H*-1,2,3-triazol-1-yl)phenyl)(phenyl)methanone.

Isolated yield: 89 mg, 0.250 mmol, 62%, colorless crystals.

Purification: pentane:ethyl acetate = 9:1.

m.p.: 127.1 – 128.9 °C.

^{1}H-NMR (400 MHz, $CDCl_3$, ppm): δ = 7.68 – 7.63 (m, 2H), 7.61 – 7.54 (m, 4H), 7.46 (ddt, J = 8.8, 7.0, 1.3 Hz, 1H), 7.33 – 7.27 (m, 2H), 0.18 (s, 9H).

^{13}C-NMR (100 MHz, $CDCl_3$, ppm): δ = 193.7, 147.4, 135.9, 135.8, 135.5, 133.8, 133.6, 131.7, 130.2, 129.9, 129.3, 128.6, 126.2, −1.2.

FT-IR (ATR, cm^{-1}): $\tilde{\nu}$ = 3116, 1656, 1594, 1495, 1448, 1393, 1313, 1244, 1201, 1157, 1112, 1040, 984, 956, 891, 832, 785, 759, 739, 709, 694, 668, 660.

MS (EI, 70 eV): *m/z* (%) = 327 (28), 315 817), 314 (100), 313 (43), 312 (50), 203 (11), 77 (19).

HR-MS (EI, 70 eV): [$C_{18}H_{18}N_3Cl_2Si$], calcd.: 355.0908; found: 355.0898.

Ethyl 2-(5-chloro-2-(4-(trimethylsilyl)-1*H*-1,2,3-triazol-1-yl)benzyl)acrylate (7e)

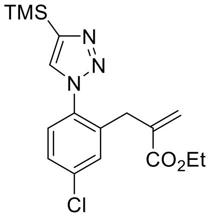

(5-Chloro-2-(4-(trimethylsilyl)-1*H*-1,2,3-triazol-1-yl)phenyl)magnesium reagent (**3a**, 0.405 mmol, 1.00 equiv) was cooled to -30 °C and CuCN·2 LiCl solution (1 M in THF, 1.20 mmol, 2.96 equiv) was added dropwise. The reaction mixture was stirred at this temperature for 30 min and ethyl 2-(bromomethyl)acrylate (193 mg, 1.0 mmol, 2.47 equiv)

was added. The reaction mixture was allowed to come to room temperature for 16 h. Saturated aq. NH_4Cl solution (5 mL) was added and the mixture was extracted with EtOAc (3 x 25 mL). The combined organic layers were dried over $MgSO_4$. The solvents were removed under reduced pressure and the crude product was subjected to column chromatography on silica yielding ethyl 2-(5-chloro-2-(4-(trimethylsilyl)-1*H*-1,2,3-triazol-1-yl)benzyl)acrylate.

Isolated yield: 113 mg, 0.311 mmol, 77%, colorless oil.

Purification: pentane:ethyl acetate = 7:3.

^{1}H-NMR (400 MHz, $CDCl_3$, ppm): δ = 7.72 (s, 1H), 7.38 (d, J = 2.3 Hz, 1H), 7.34 (dd, J = 8.4, 2.3 Hz, 1H), 7.26 (d, J = 8.4 Hz, 1H), 6.18 (d, J = 1.2 Hz, 1H), 5.27 (q, J = 1.3 Hz, 1H), 4.14 (q, J = 7.1 Hz, 2H), 3.52 (s, 2H), 1.24 (t, J = 7.1 Hz, 3H), 0.36 (s, 9H).

^{13}C-NMR (100 MHz, $CDCl_3$, ppm): δ = 166.2, 146.7, 137.8, 136.9, 135.7, 135.1, 131.1, 130.9, 127.8, 127.8, 127.8, 61.1, 33.8, 14.3, −1.00.

FT-IR (ATR, cm^{-1}): $\tilde{\nu}$ = 2956, 1713, 1631, 1598, 1499, 1478, 1407, 1368, 1327, 1300, 1249, 1203, 1186, 1145, 1114, 1094, 1033, 997, 983, 997, 983, 951, 932, 838, 818, 757, 709, 697, 662, 677.

MS (EI, 70 eV): *m/z* (%) = 306 (15), 263 (23), 261 (55), 249 (24), 248 (28), 215 (25), 190 (23), 189 (33), 75 (66), 73 (100).

HR-MS (EI, 70 eV): [$C_{17}H_{22}ClN_3O_2Si$], calcd.: 363.1170; found: 363.1156.

1-(5-fluoro-4'-(trifluoromethoxy)-[1,1'-biphenyl]-2-yl)-1*H*-1,2,3-triazole (8)

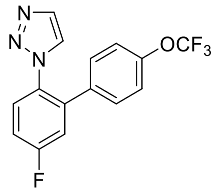

According to the literature[6], 1-(5-fluoro-4'-(trifluoromethoxy)-[1,1'-biphenyl]-2-yl)-4-(trimethylsilyl)-1*H*-1,2,3-triazole (**1h**, 119 mg, 0.3 mmol, 1.00 equiv) was dissolved in THF (0.3 mL) and TBAF (1 M in THF, 0.45 mmol, 1.50 equiv) was added. The reaction mixture was stirred for 16 h. Saturated aq. $NaHCO_3$ solution (5 mL) was added and the mixture was extracted with EtOAc (3 x 25 mL). The combined organic layers were dried over $MgSO_4$. The solvents were removed under reduced pressure and the crude product was subjected to column chromatography on silica yielding the title compound.

Isolated yield: 88 mg, 0.27 mmol, 91%, colorless oil.

Purification: pentane:ethyl acetate = 9:1.

^{1}H-NMR (400 MHz, $CDCl_3$, ppm): δ = 7.55 (d, J = 1.1 Hz, 1H), 7.52 (dd, J = 8.6, 5.1 Hz, 1H), 7.19 (d, J = 1.1 Hz, 1H), 7.18 – 7.12 (m, 2H), 7.04 (m, 4H).

^{13}C-NMR (100 MHz, CDCl$_3$, ppm): *δ* = 163.0 (d, *J* = 251.7 Hz), 149.4 (q, *J* = 1.9 Hz), 138.4 (d, *J* = 8.6 Hz), 134.8 (d, *J* = 1.5 Hz), 133.9, 131.3 (d, *J* = 3.3 Hz), 129.9, 129.1 (d, *J* = 9.2 Hz), 125.9, 121.2, 120.4 (q, *J* = 258.0 Hz), 117.9 (d, *J* = 23.5 Hz), 116.0 (d, *J* = 22.6 Hz).

^{19}F NMR (377 MHz, CDCl3, ppm): δ = −57.81 (s), −109.88 (td, J = 8.3, 5.1 Hz).

FT-IR (ATR, cm^{-1}): $\tilde{\nu}$ = 1617, 1593, 1580, 1519, 1501, 1474, 1001, 1253, 1206, 1161, 1107, 1089, 1039, 1026, 1018, 982, 945, 922, 890, 853, 826, 806, 780, 701, 674, 660.

MS (EI, 70 eV): *m/z* (%) = 323 (40), 295 (100), 226 (13), 210 (61), 208 (29), 198 (56), 183 (19), 170 (32), 157 (38).

HR-MS (EI, 70 eV): [$C_{15}H_9N_3OF_4$], calcd.: 323.0682; found: 323.0677.

5-(5-fluoro-4'-(trifluoromethoxy)-2-(4-(trimethylsilyl)-1*H*-1,2,3-triazol-1-yl)-[1,1'-biphenyl]-3-yl)-1-methyl-1*H*-indole (10)

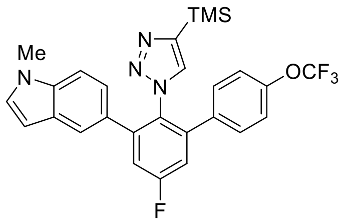

Following **TP3** (5-fluoro-4'-(trifluoromethoxy)-2-(4-(trimethylsilyl)-1*H*-1,2,3-triazol-1-yl)-[1,1'-biphenyl]-3-yl)magnesium reagent (**9**, 0.400 mmol, 1.00 equiv) was coupled with 5-bromo-1-methyl-1*H*-indole (0.85 mmol, 179 mg, 2.34 equiv).

Isolated yield: 184 mg, 0.351 mmol, 88%, pale yellow crystals.

Purification: pentane:ethyl acetate = 8:2.

m.p.: 64.9 – 66.6 °C.

^{1}H-NMR (400 MHz, CDCl$_3$, ppm): *δ* = 7.37 (dd, *J* = 1.7, 0.7 Hz, 1H), 7.33 (dd, *J* = 8.9, 2.9 Hz, 1H), 7.19 – 7.11 (m, 4H), 7.10 – 7.04 (m, 3H), 7.03 (d, *J* = 3.1 Hz, 1H), 6.89 (dd, *J* = 8.5, 1.7 Hz, 1H), 6.39 (dd, *J* = 3.2, 0.9 Hz, 1H), 3.74 (s, 3H), 0.08 (s, 9H).

^{13}C-NMR (100 MHz, CDCl$_3$, ppm): *δ* = 162.5 (d, J = 251.1 Hz), 149.0 (d, J = 2.0 Hz), 146.1, 144.2 (d, J = 9.2 Hz), 141.1 (d, J = 9.2 Hz), 136.4, 135.87 (d, J = 1.8 Hz), 132.6, 129.9, 129.7, 128.5, 127.9 (d, J = 1.6 Hz), 121.8, 121.1, 120.7, 120.5 (q, J = 257.7 Hz), 117.6 (d, J = 22.4 Hz), 115.8 (d, J = 23.3 Hz), 109.2, 101.5, 33.0, -1.3.

^{19}F NMR (377 MHz, CDCl3, ppm): δ = −57.86 (s), −110.53 (t, *J* = 8.7 Hz).

FT-IR (ATR, cm^{-1}): $\tilde{\nu}$ = 2956, 1594, 1511, 1511, 1494, 1463, 1446, 1422, 1325, 1247, 1204, 1160, 1103, 1080, 1035, 1019, 984, 955, 921, 873, 838, 801, 760, 722, 679.

MS (EI, 70 eV): *m/z* (%) = 524 (6), 497 (22), 496 (64), 495 (100), 482 (29), 481 (87), 424 (18), 415 (32), 338 (23), 337 (24), 335 (59).

HR-MS (EI, 70 eV): [$C_{27}H_{24}N_4OSi$], calcd.: 524.1656; found: 524.1645.

4-Bromo-1-(5-fluoro-4'-(trifluoromethoxy)-[1,1'-biphenyl]-2-yl)-1*H*-1,2,3-triazole (11)

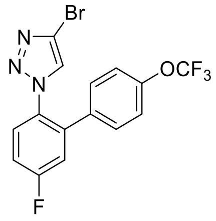

1-(5-fluoro-4'-(trifluoromethoxy)-[1,1'-biphenyl]-2-yl)-4-(trimethylsilyl)-1*H*-1,2,3-triazole (**1h**, 790 mg, 2.00 mmol, 1.00 equiv) was dissolved in DMF (4 mL) cooled with an icebath to 5 °C and DBDMH (686 mg, 2.40 mmol, 1.20 equiv) was added and the reaction mixture was stirred for 16 h. After removal of the solvent the crude product was subjected to column chromatography on silica yielding the title compound.

Isolated yield: 769 mg, 1.92 mmol, 93%, pale yellow crystals.

Purification: pentane:ethyl acetate = 9:1.

m.p.: 77.8 – 79.5 °C.

^{1}H-NMR (400 MHz, $CDCl_3$, ppm): δ = 7.37 (dd, *J* = 8.6, 5.1 Hz, 1H), 7.08 – 7.00 (m, 3H), 6.99 – 6.94 (m, 2H), 6.93 – 6.88 (m, 2H).

^{13}C-NMR (100 MHz, $CDCl_3$, ppm): δ = 163.3 (d, *J* = 252.7 Hz), 149.6 (t, *J* = 1.9 Hz), 138.6 (d, *J* = 8.6 Hz), 134.4 (d, *J* = 1.5 Hz), 130.8 (d, *J* = 3.4 Hz), 129.9, 129.1 (d, *J* = 9.3 Hz), 126.0, 121.4, 120.8, 120.5 (q, *J* = 258.1 Hz), 118.1 (d, *J* = 23.7 Hz), 116.2 (d, *J* = 22.8 Hz).

^{19}F NMR (377 MHz, CDCl3, ppm): δ = −57.81 (s), −108.80 (td, *J* = 7.9, 5.1 Hz).

FT-IR (ATR, cm^{-1}): $\tilde{\nu}$ = 3158, 1615, 1594, 1580, 1516, 1500, 1478, 1442, 1395, 1278, 1246, 1221, 1205, 1190, 1164, 1104, 1040, 1034, 1017, 991, 976, 951, 921, 893, 881, 853, 830, 817, 806, 746, 698, 658, 676.

MS (EI, 70 eV): *m/z* (%) = 375 (38), 372 (37), 295 (21), 294 (100), 290 (26), 288 (27), 266 (18), 209 (50), 208 (98), 197 (20), 196 (24), 169 (24), 158 (11), 157 (33).

HR-MS (EI, 70 eV): [$C_{15}H_8N_3O_2BrF_4$], calcd.: 400.9787; found: 400.9786.

4-(4-Chlorophenyl)-1-(5-fluoro-4'-(trifluoromethoxy)-[1,1'-biphenyl]-2-yl)-1*H*-1,2,3-triazole (12)

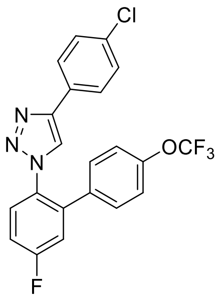

According to the literature[7], 4-bromo-1-(5-fluoro-4'-(trifluoromethoxy)-[1,1'-biphenyl]-2-yl)-1*H*-1,2,3-triazole was (**1h**, 99 mg, 0.250 mmol, 1.00 equiv), K_2CO_3 (69 mg. 0.500 mmol, 2.00 equiv), $Pd(PPh_3)_4$ (29 mg, 0.025 mmol 10 mol%) and (4-chlorophenyl)boronic acid (59 mg, 0.375, 1.5 equiv) were added to a flask under argon. Then dioxane (2.25 ml) and water (0.75 ml) were added and the reaction mixture was heated for 16 h at 100 °C. Saturated aq. NH_4Cl solution (5 mL) was added and the mixture was extracted with EtOAc (3 x 25 mL). The combined organic layers were dried over $MgSO_4$. The solvents were removed under reduced pressure and the crude product was subjected to column chromatography on silica yielding 4-(4-chlorophenyl)-1-(5-fluoro-4'-(trifluoromethoxy)-[1,1'-biphenyl]-2-yl)-1H-1,2,3-triazole.

Isolated yield: 93 mg, 0.22 mmol, 86%, pale orange crystals.

Purification: pentane:ethyl acetate = 9:1.

m.p.: 144.7 – 146.3 °C.

^{1}H-NMR (400 MHz, $CDCl_3$, ppm): *δ* = 7.47 – 7.42 (m, 1H), 7.42 – 7.37 (m, 2H), 7.19 (s, 1H), 7.17 – 7.12 (m, 2H), 7.10 – 7.02 (m, 2H), 6.95 (s, 4H).

^{13}C-NMR (100 MHz, $CDCl_3$, ppm): *δ* = 163.1 (d, *J* = 252.0 Hz), 149.5 (d, *J* = 2.0 Hz), 147.9, 138.3 (d, *J* = 8.6 Hz), 134.9 (d, *J* = 1.5 Hz), 134.4, 131.2 (d, *J* = 3.2 Hz), 130.0, 129.2, 129.0 (d, *J* = 9.2 Hz), 128.5, 127.1, 121.8, 121.4, 120.5 (q, *J* = 257.9 Hz), 118.0 (d, *J* = 23.6 Hz), 116.2 (d, J = 22.8 Hz).

^{19}F NMR (377 MHz, CDCl3, ppm): δ = −58.32 , −110.08 (ddd, *J* = 8.6, 7.6, 5.1 Hz).

FT-IR (ATR, cm^{-1}): $\tilde{\nu}$ = 1610, 1519, 1054, 1475, 1426, 2317, 1301, 1279, 1239, 1224, 1206, 1182, 1112, 1095, 1083, 1035, 1018, 1011, 993, 967, 945, 924, 893, 82, 843, 832, 846, 824, 805, 138, 719, 704, 686, 675, 662.

MS (EI, 70 eV): *m/z* (%) = 407 (32), 406 (30), 405 (100), 370 (32), 369 (52), 318 (11), 286 (11), 285 (44), 272 (17), 170 (11), 150 (18).

HR-MS (EI, 70 eV): [$C_{21}H_{12}N_3O_2ClF_4$], calcd.: 433.0605; found: 433.0600.

^{1}H-NMR and ^{13}C-NMR and ^{19}F-NMR Spectra

1-(5-Chloro-4'-methoxy-[1,1'-biphenyl]-2-yl)-4-(trimethylsilyl)-1H-1,2,3-triazole (1b)

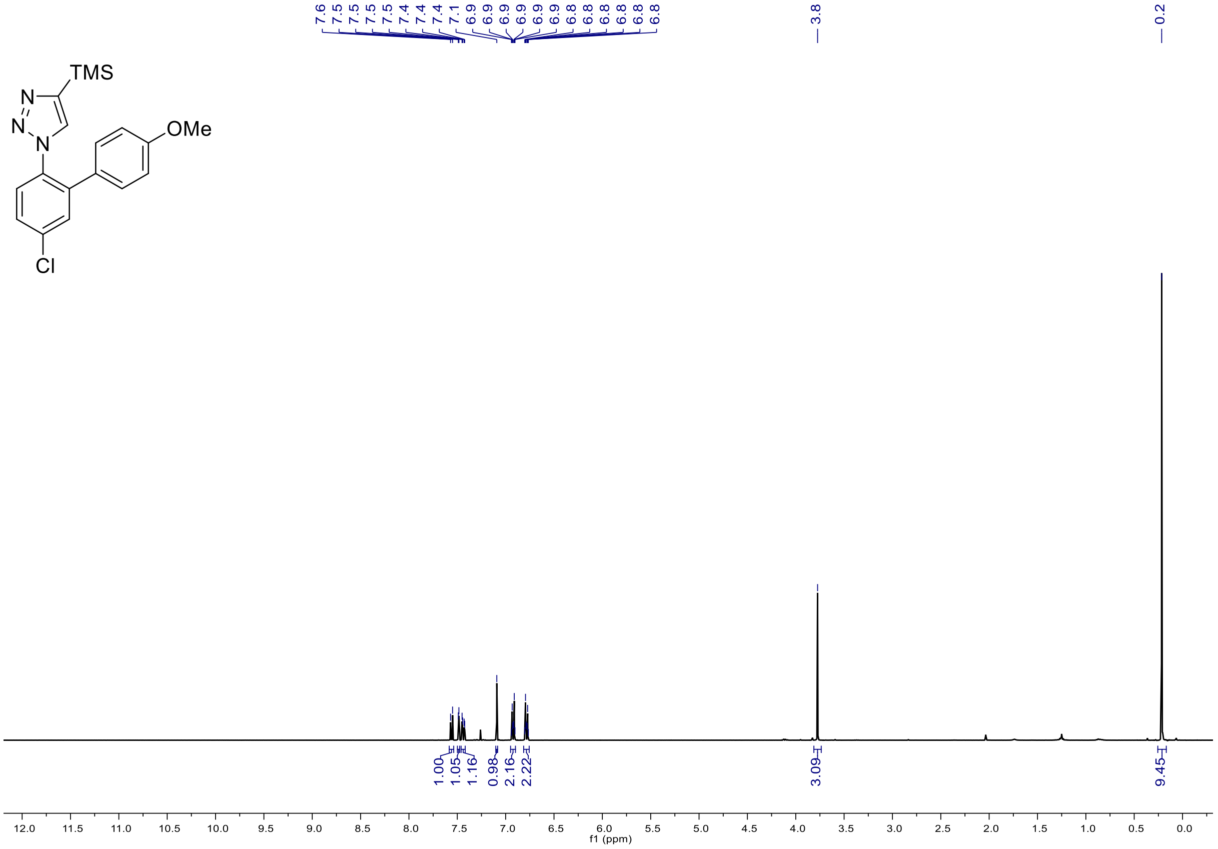

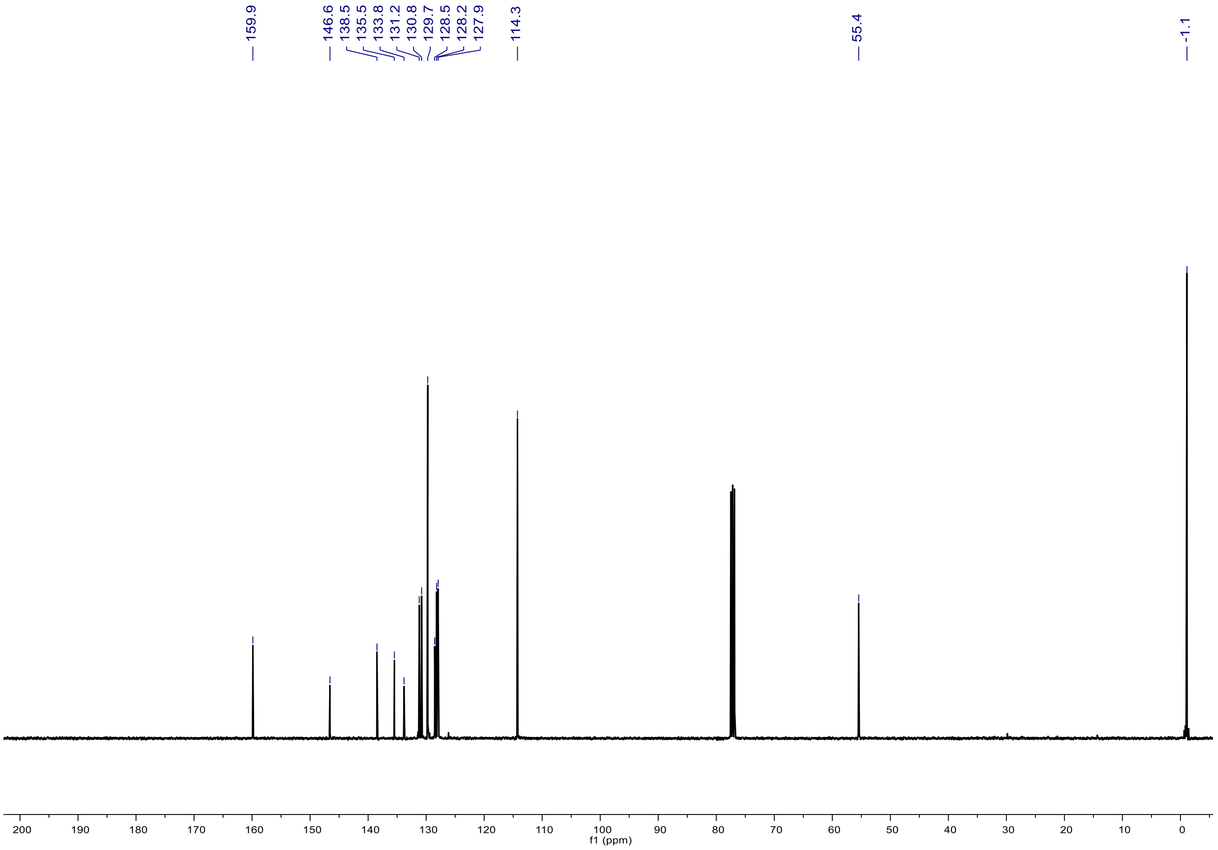

Supplementary Figure 14: ^{1}H-NMR spectrum (top) and ^{13}C-NMR spectrum (bottom) of **1b**.

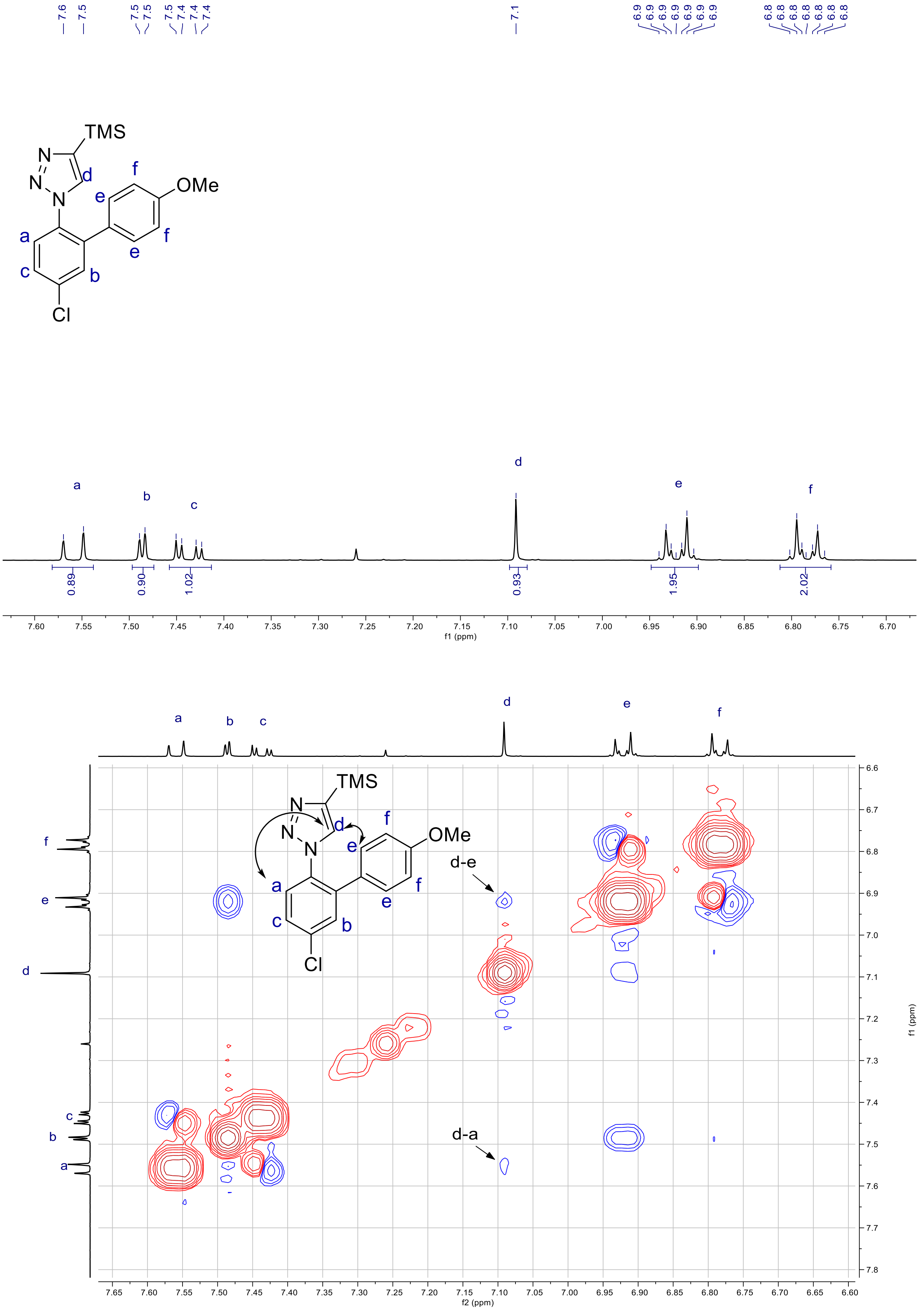

Supplementary Figure 15: ^{1}H-NMR spectrum (top) and NOESY-NMR spectrum (bottom) of **1b**.

1-(3'-(1,3-Dioxolan-2-yl)-[1,1'-biphenyl]-2-yl)-4-(trimethylsilyl)-1*H*-1,2,3-triazole (1c)

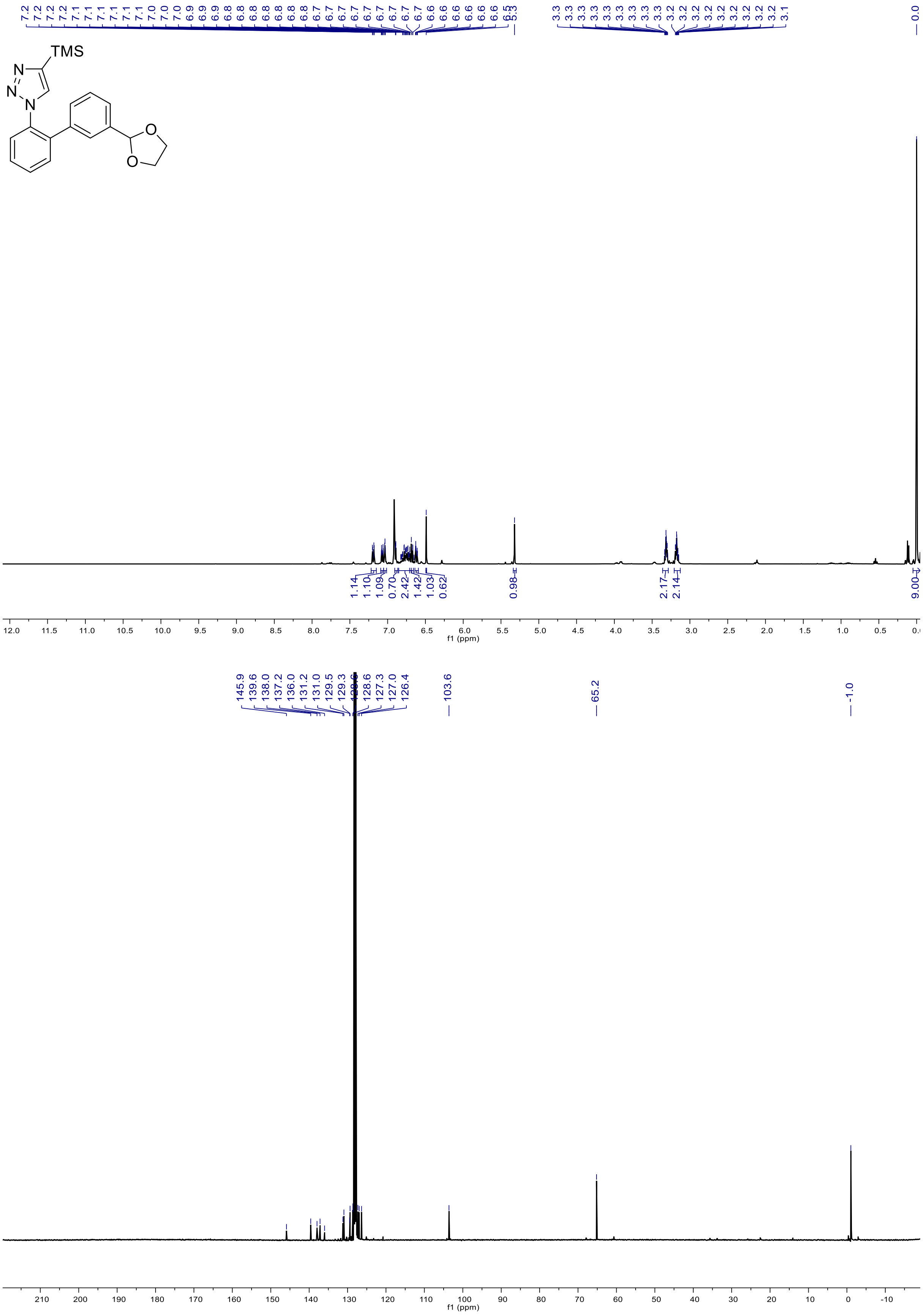

Supplementary Figure 16: ^{1}H-NMR spectrum (top) and ^{13}C-NMR spectrum (bottom) of **1c**.

5'-Chloro-3-fluoro-2'-(4-(trimethylsilyl)-1*H*-1,2,3-triazol-1-yl)-[1,1'-biphenyl]-4-carbonitrile (1d)

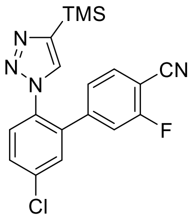

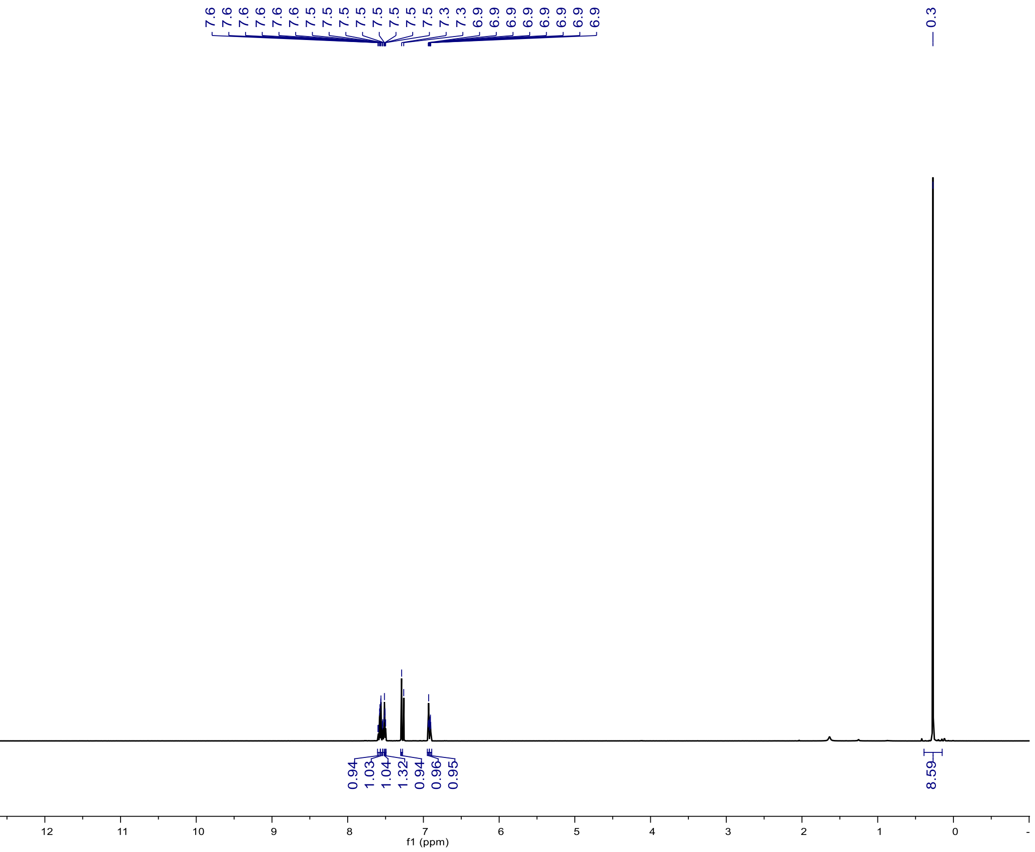

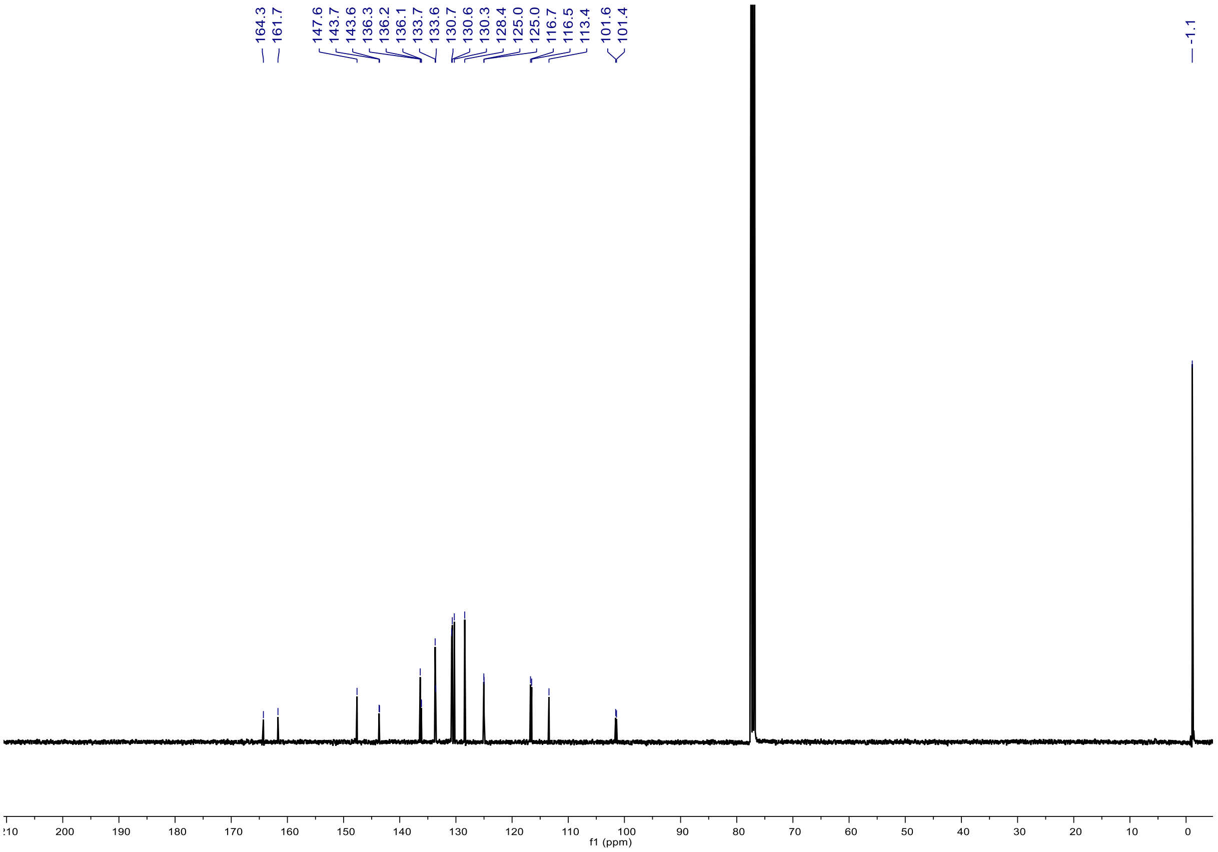

Supplementary Figure 17: ^{1}H-NMR spectrum (top) and ^{13}C-NMR spectrum (bottom) of **1d**.

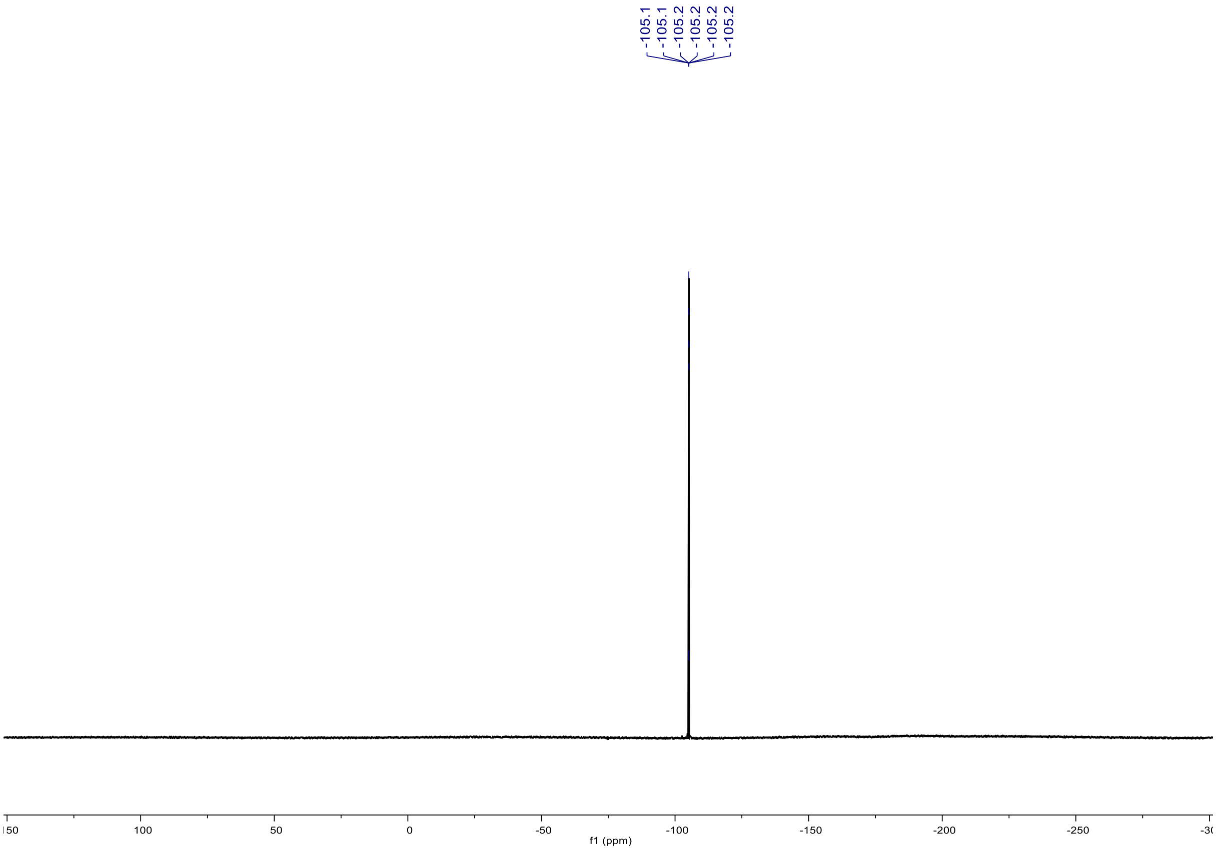

Supplementary Figure 18: ^{19}F-NMR spectrum of **1d**.

3,3'-Difluoro-2'-(4-(trimethylsilyl)-1*H*-1,2,3-triazol-1-yl)-[1,1'-biphenyl]-4-carbonitrile (1e)

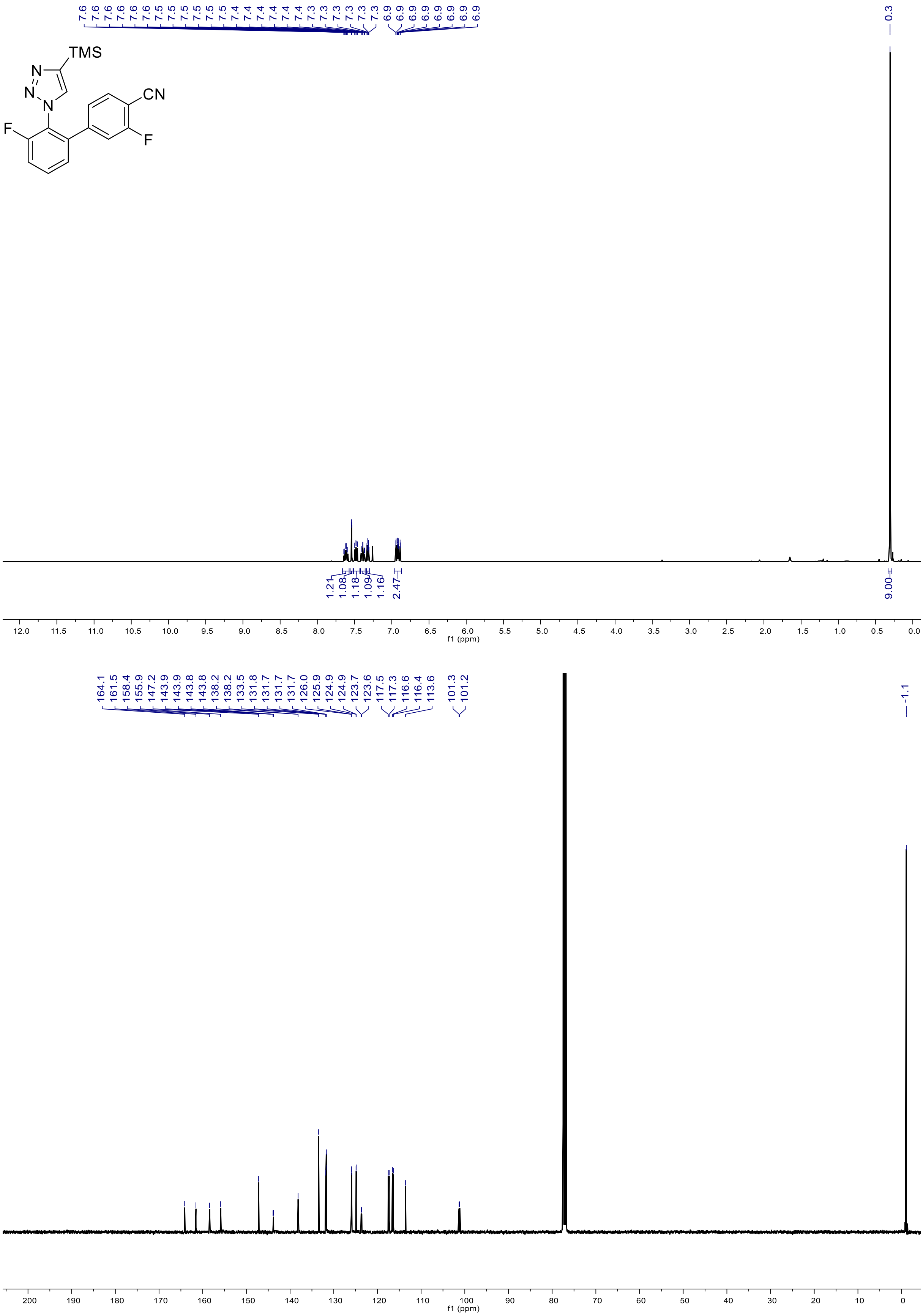

Supplementary Figure 19: ^{1}H-NMR spectrum (top) and ^{13}C-NMR spectrum (bottom) of **1e**.

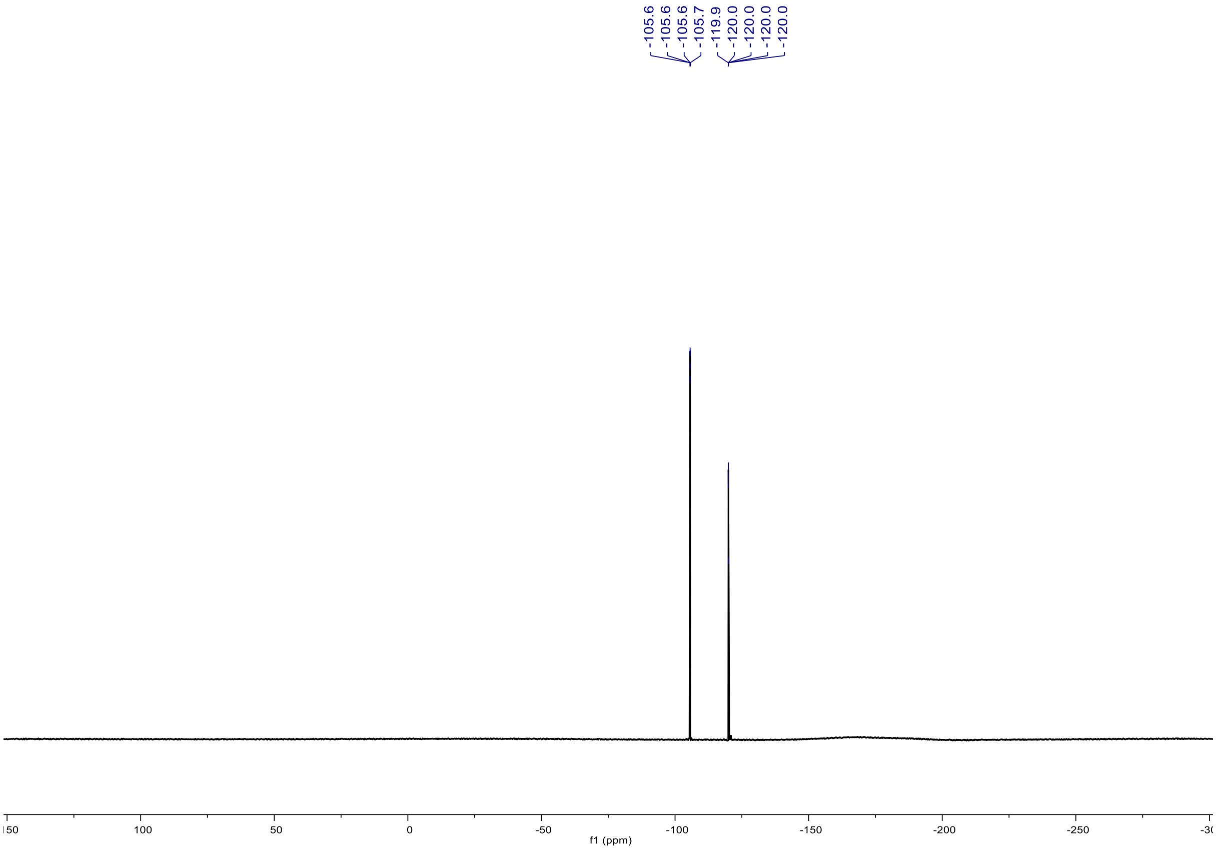

Supplementary Figure 20: ^{19}F-NMR spectrum of **1e**.

Ethyl 5'-chloro-2'-(4-(trimethylsilyl)-1*H*-1,2,3-triazol-1-yl)-[1,1'-biphenyl]-3-carboxylate (1f)

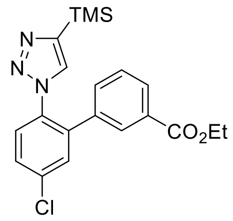

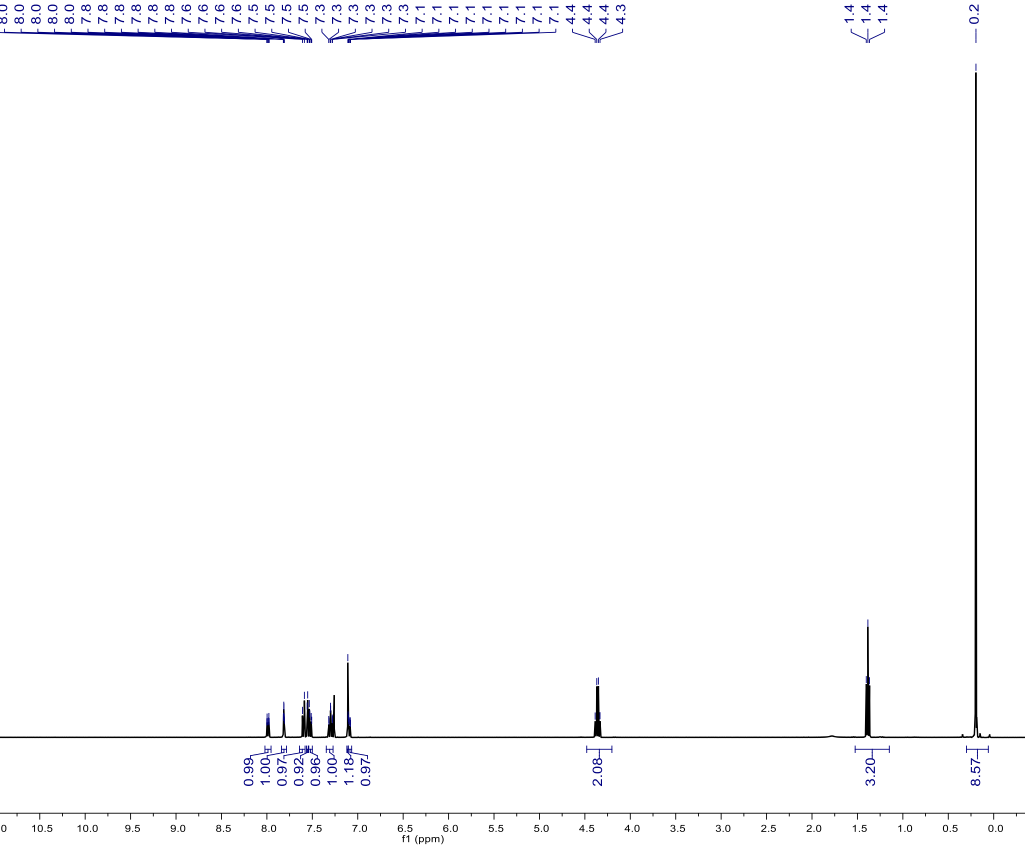

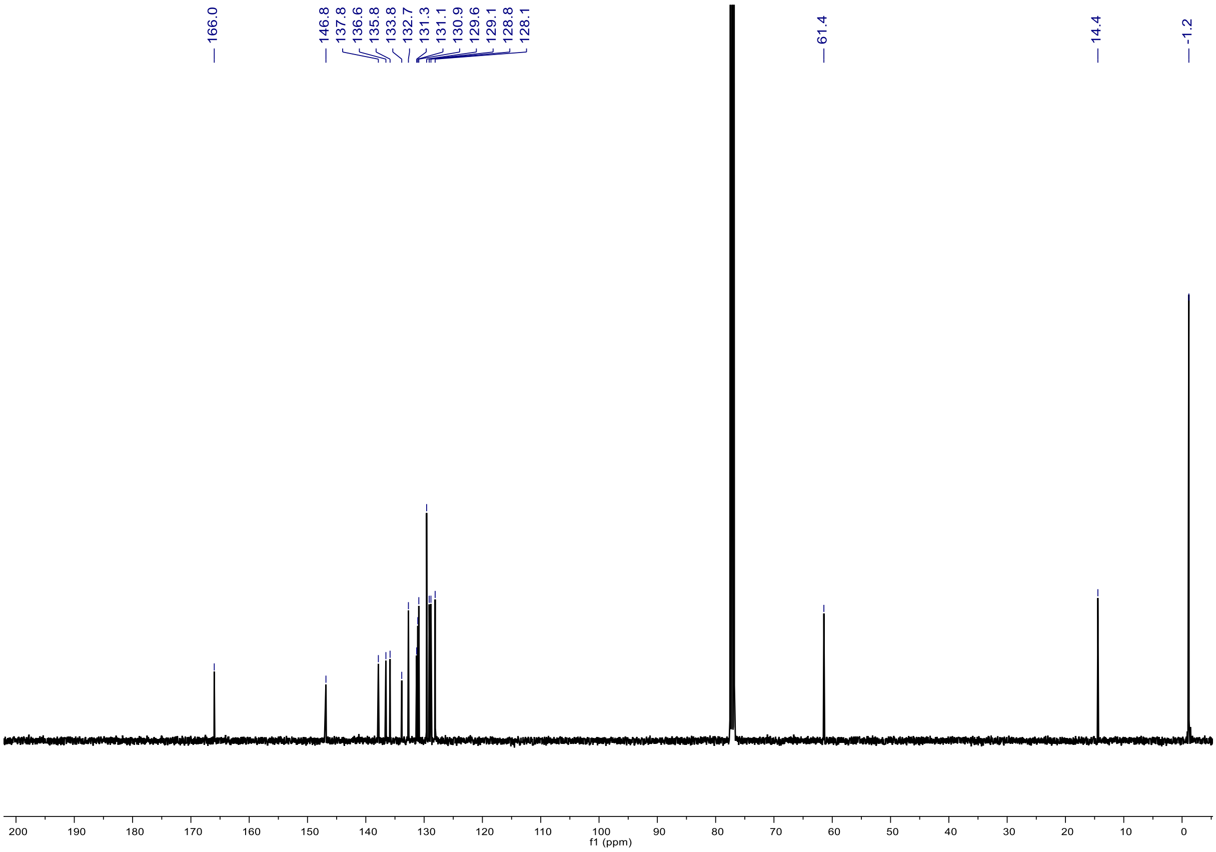

Supplementary Figure 21: ^{1}H-NMR spectrum (top) and ^{13}C-NMR spectrum (bottom) of **1f**.

1-(4-Fluoro-2-(naphthalen-1-yl)phenyl)-4-(trimethylsilyl)-1*H*-1,2,3-triazole (1g)

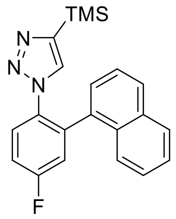

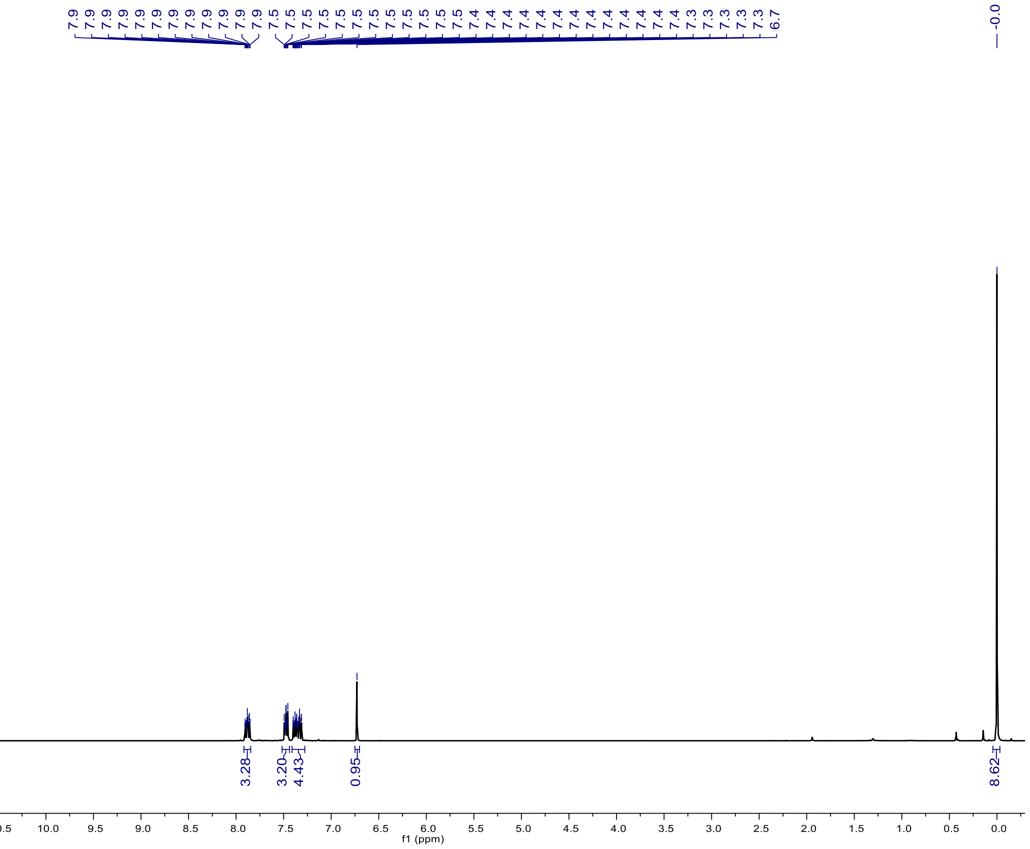

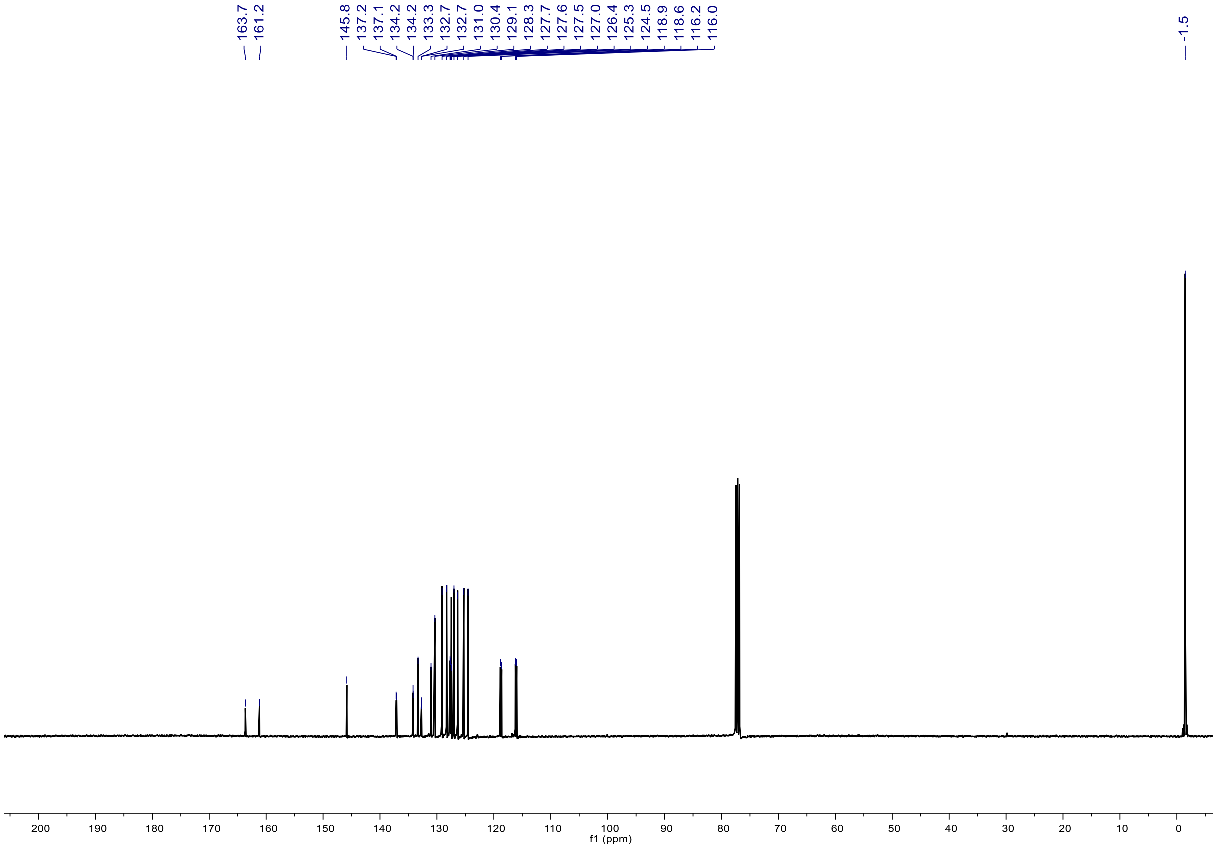

Supplementary Figure 22: ^{1}H-NMR spectrum (top) and ^{13}C-NMR spectrum (bottom) of **1g**.

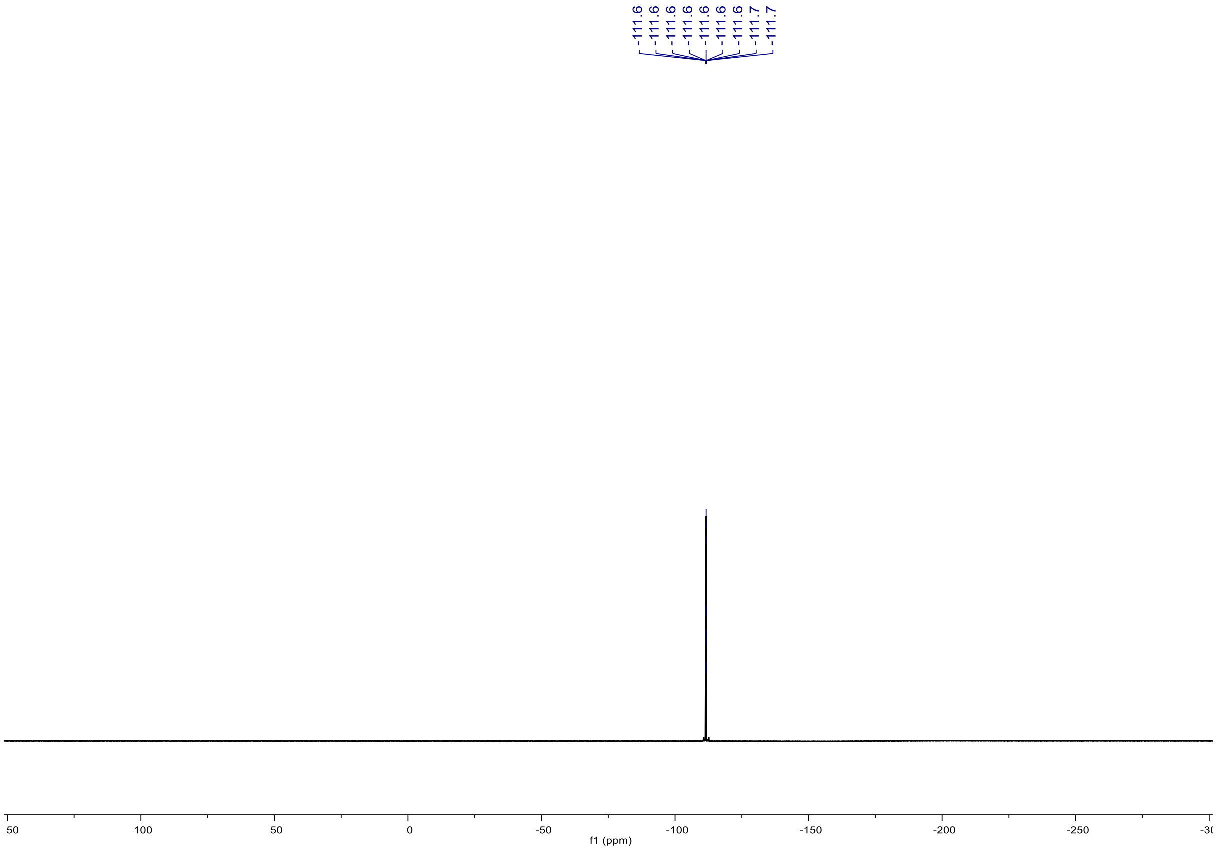

Supplementary Figure 23: ^{19}F-NMR spectrum of **1g**.

1-(5-Fluoro-4'-(trifluoromethoxy)-[1,1'-biphenyl]-2-yl)-4-(trimethylsilyl)-1*H*-1,2,3-triazole (1h)

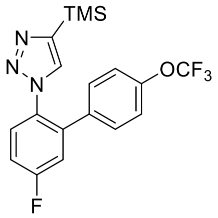

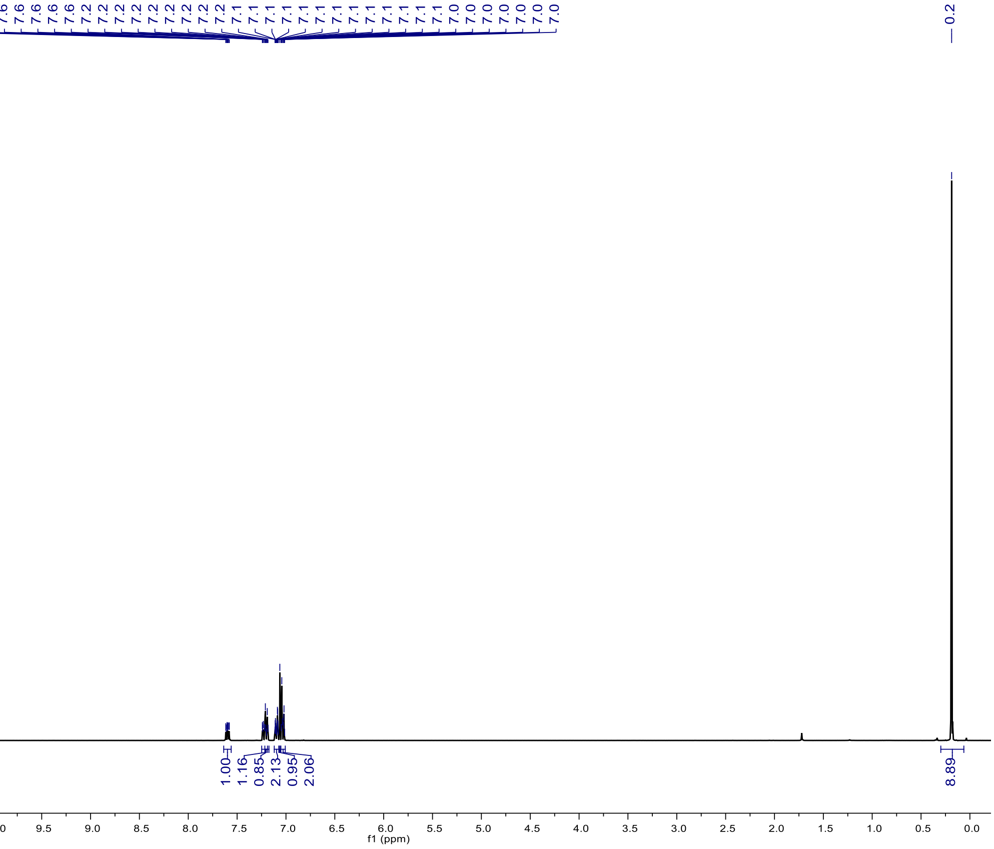

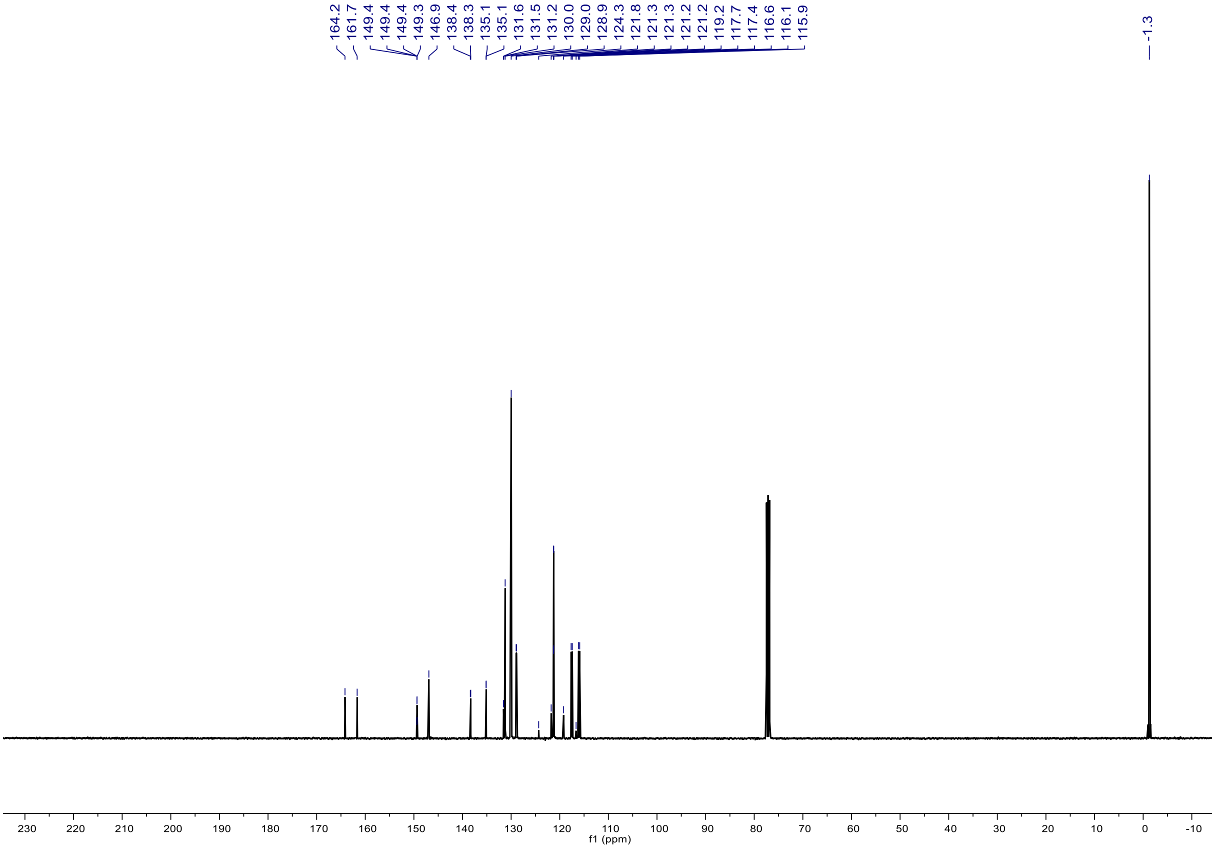

Supplementary Figure 24: ^{1}H-NMR spectrum (top) and ^{13}C-NMR spectrum (bottom) of **1h**.

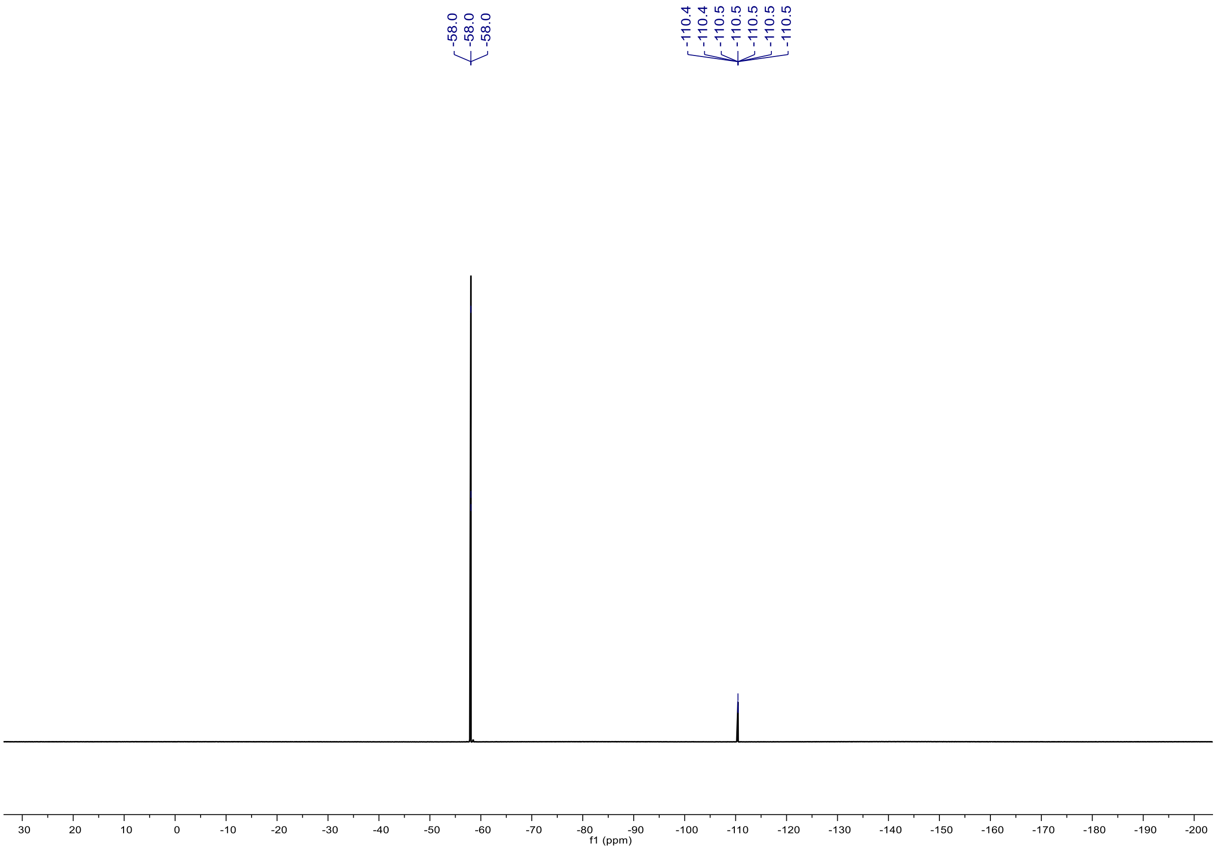

Supplementary Figure 25: ^{19}F-NMR spectrum of **1h**.

1-(5-Fluoro-4'-(pentafluoro-λ^6-sulfaneyl)-[1,1'-biphenyl]-2-yl)-4-(trimethylsilyl)-1*H*-1,2,3-triazole (1i)

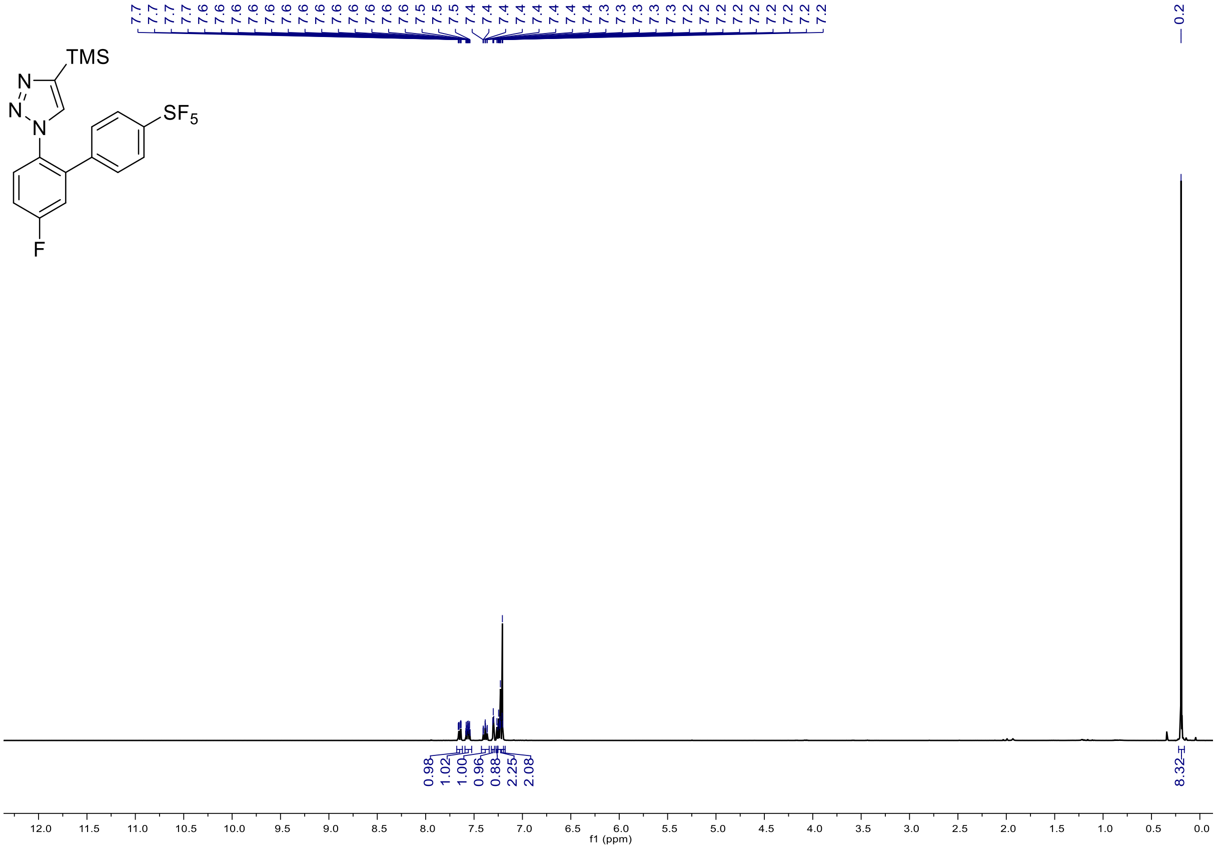

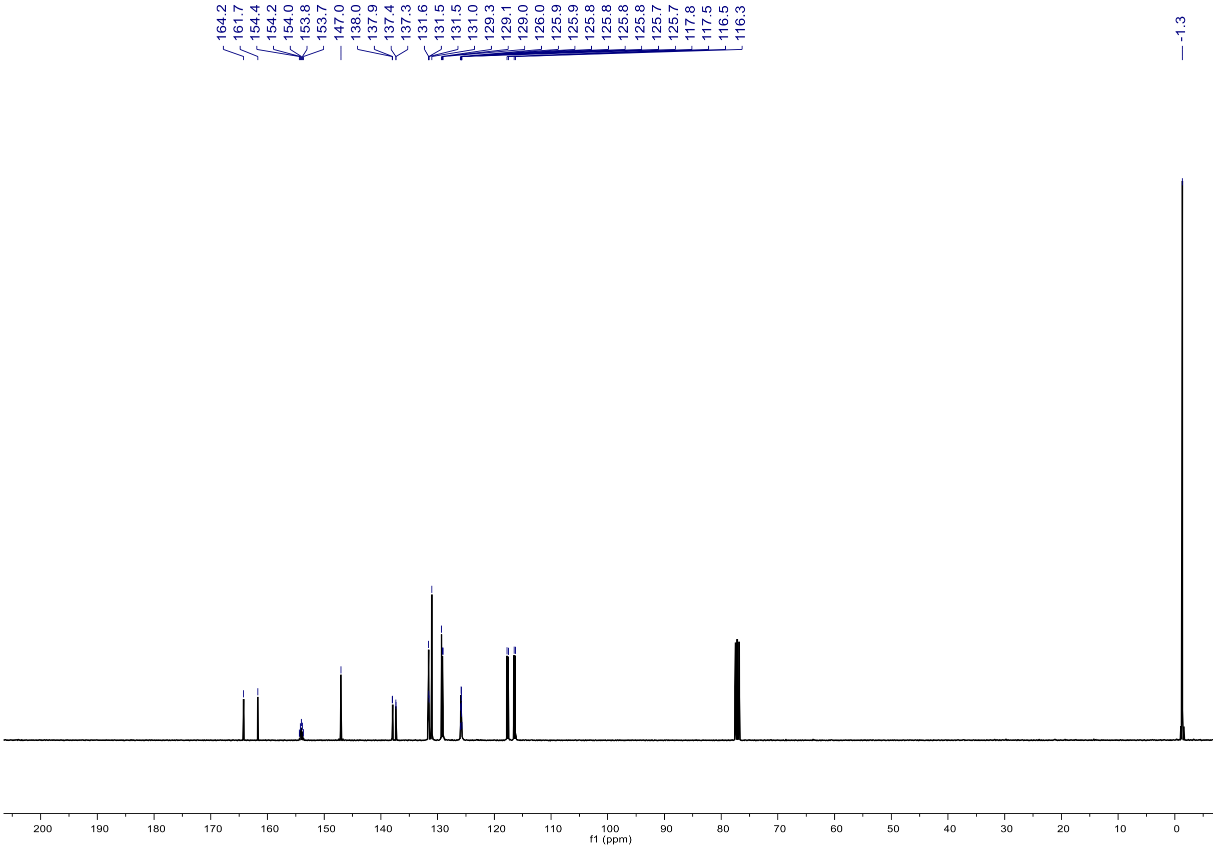

Supplementary Figure 26: ^{1}H-NMR spectrum (top) and ^{13}C-NMR spectrum (bottom) of **1i**.

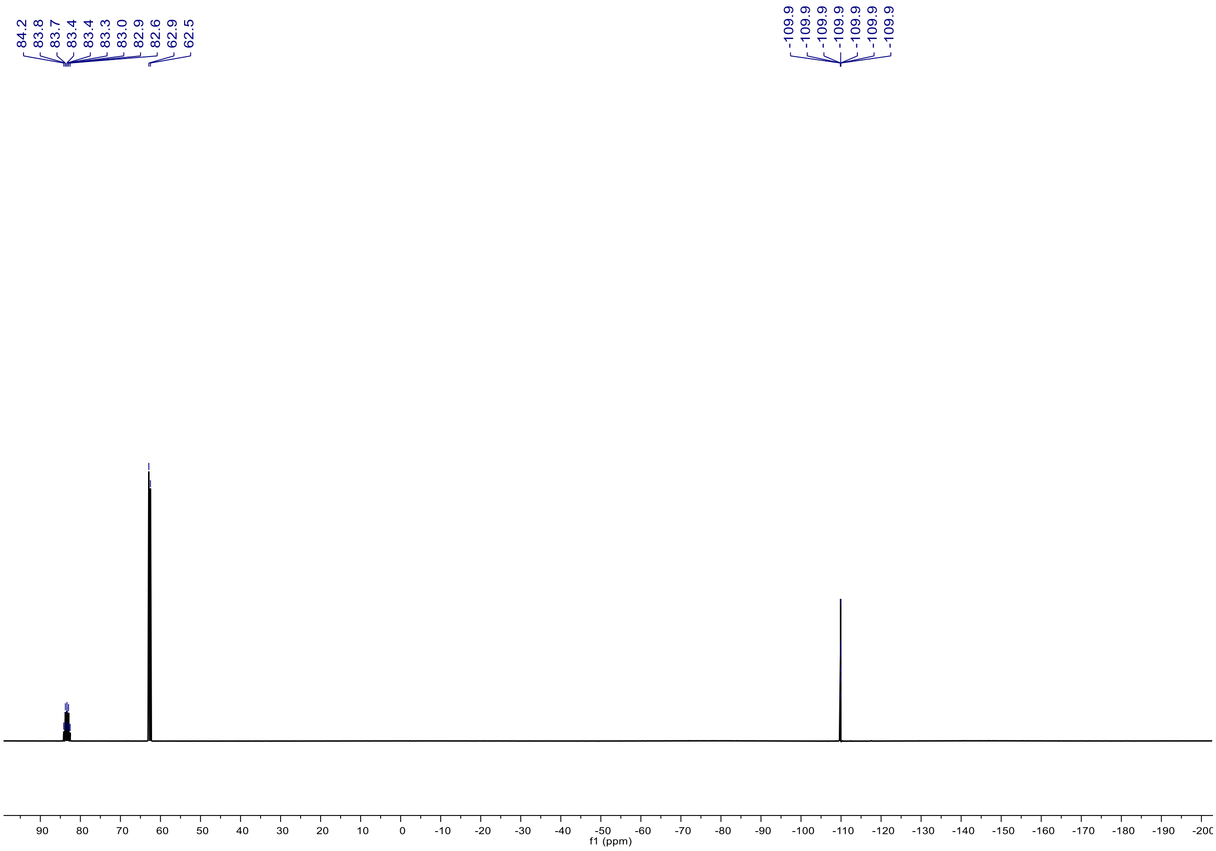

Supplementary Figure 27: ^{19}F-NMR spectrum of **1i**.

1-(5-Methoxy-3'-(trifluoromethyl)-[1,1'-biphenyl]-2-yl)-4-(trimethylsilyl)-1*H*-1,2,3-triazole (1j)

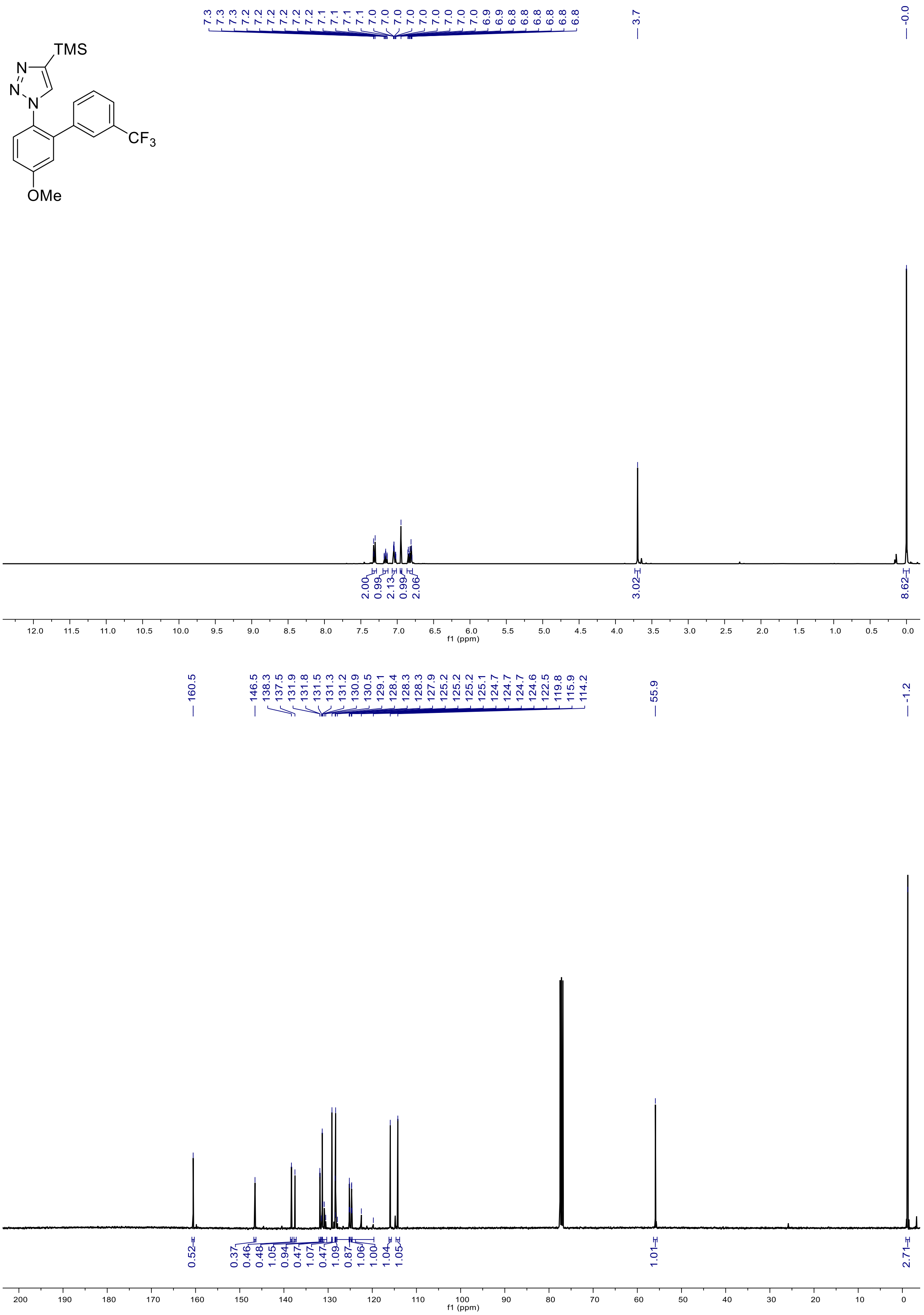

Supplementary Figure 28: ^{1}H-NMR spectrum (top) and ^{13}C-NMR spectrum (bottom) of **1j**.

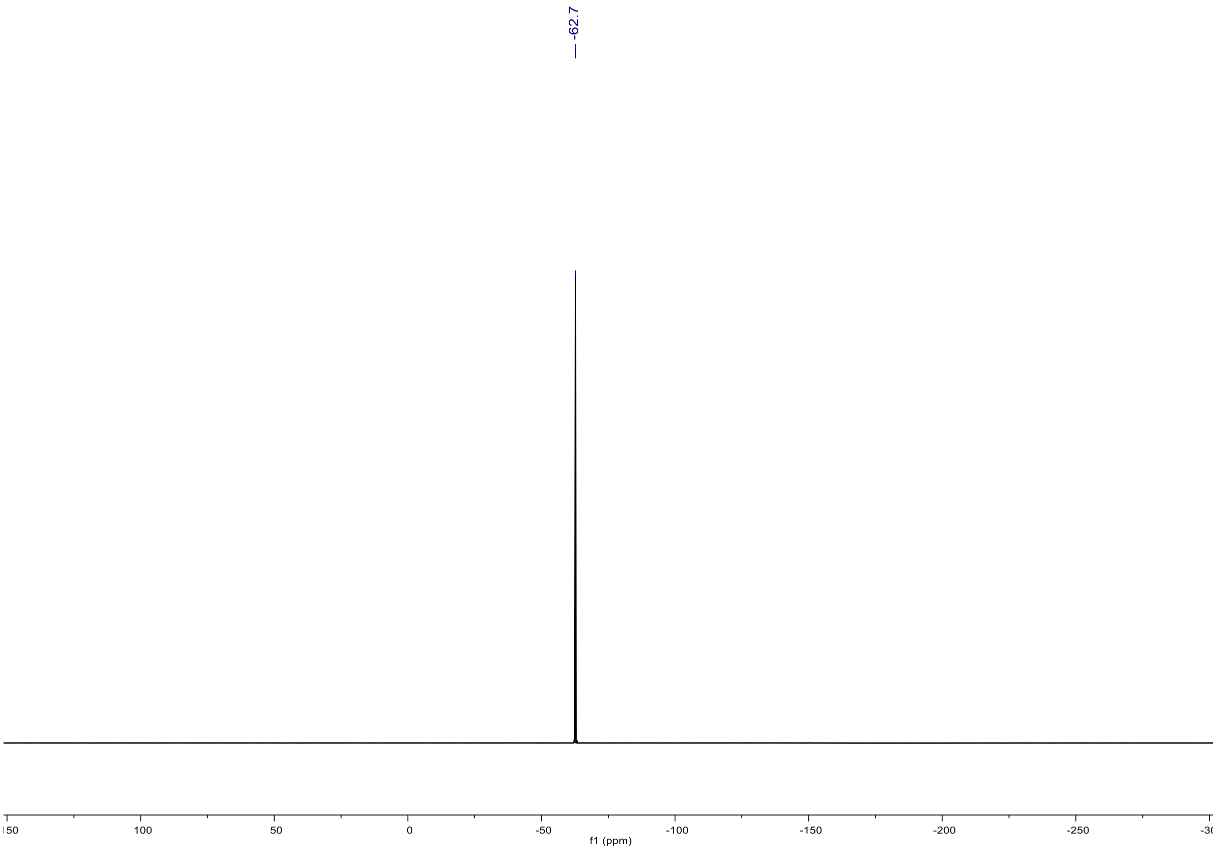

Supplementary Figure 29: ^{19}F-NMR spectrum of **1j**.

1-(5-Chloro-2'-fluoro-[1,1'-biphenyl]-2-yl)-4-(trimethylsilyl)-1*H*-1,2,3-triazole (1k)

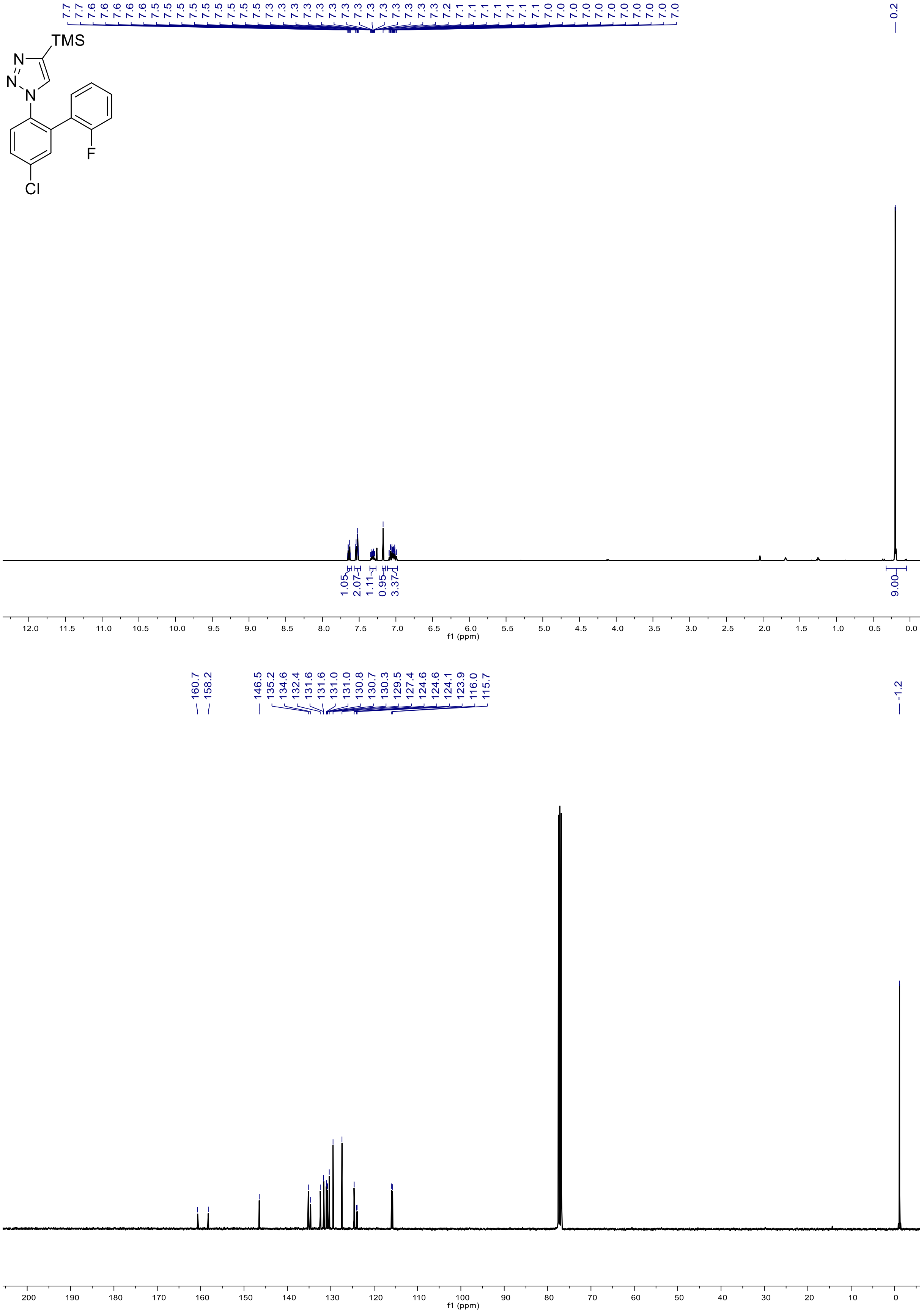

Supplementary Figure 30: ^{1}H-NMR spectrum (top) and ^{13}C-NMR spectrum (bottom) of **1k**.

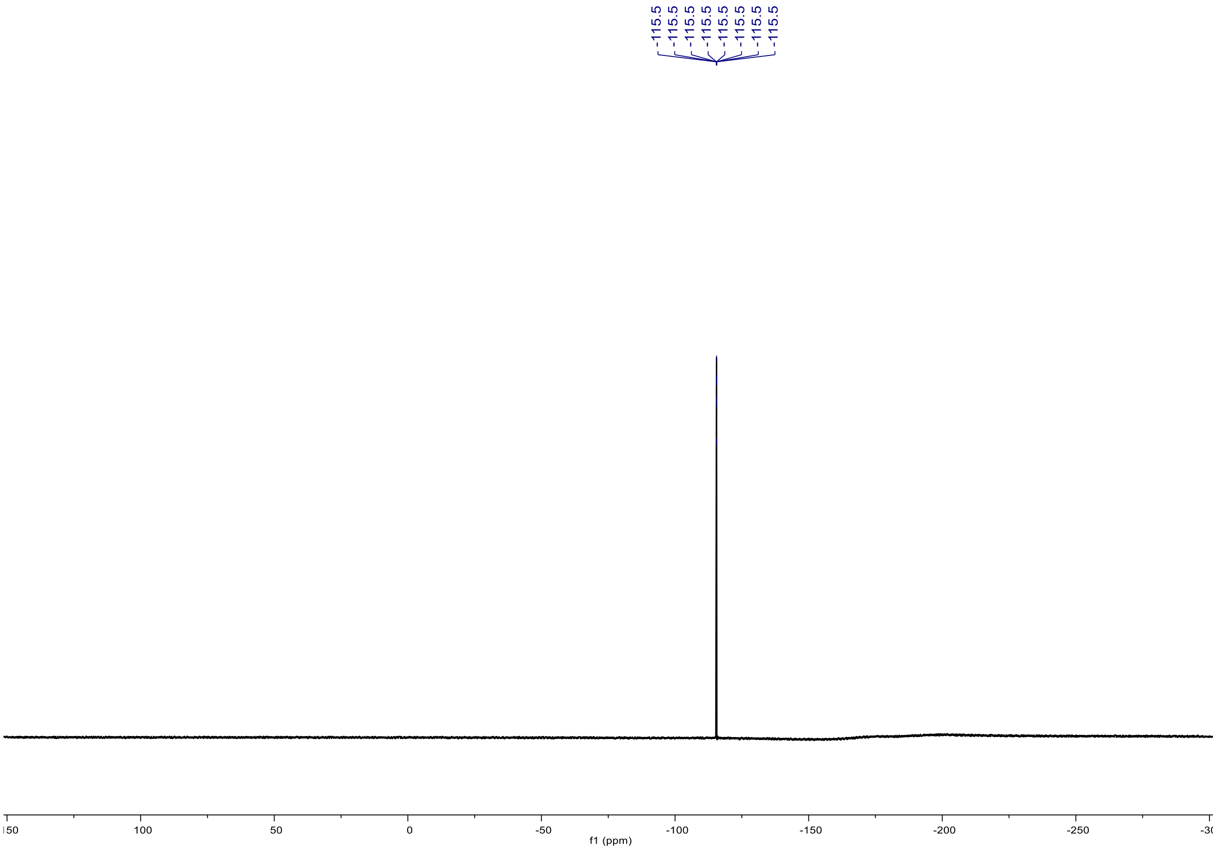

Supplementary Figure 31: ^{19}F-NMR spectrum of **1k**.

3-(5-Chloro-2-(4-(trimethylsilyl)-1*H*-1,2,3-triazol-1-yl)phenyl)pyridine (1l)

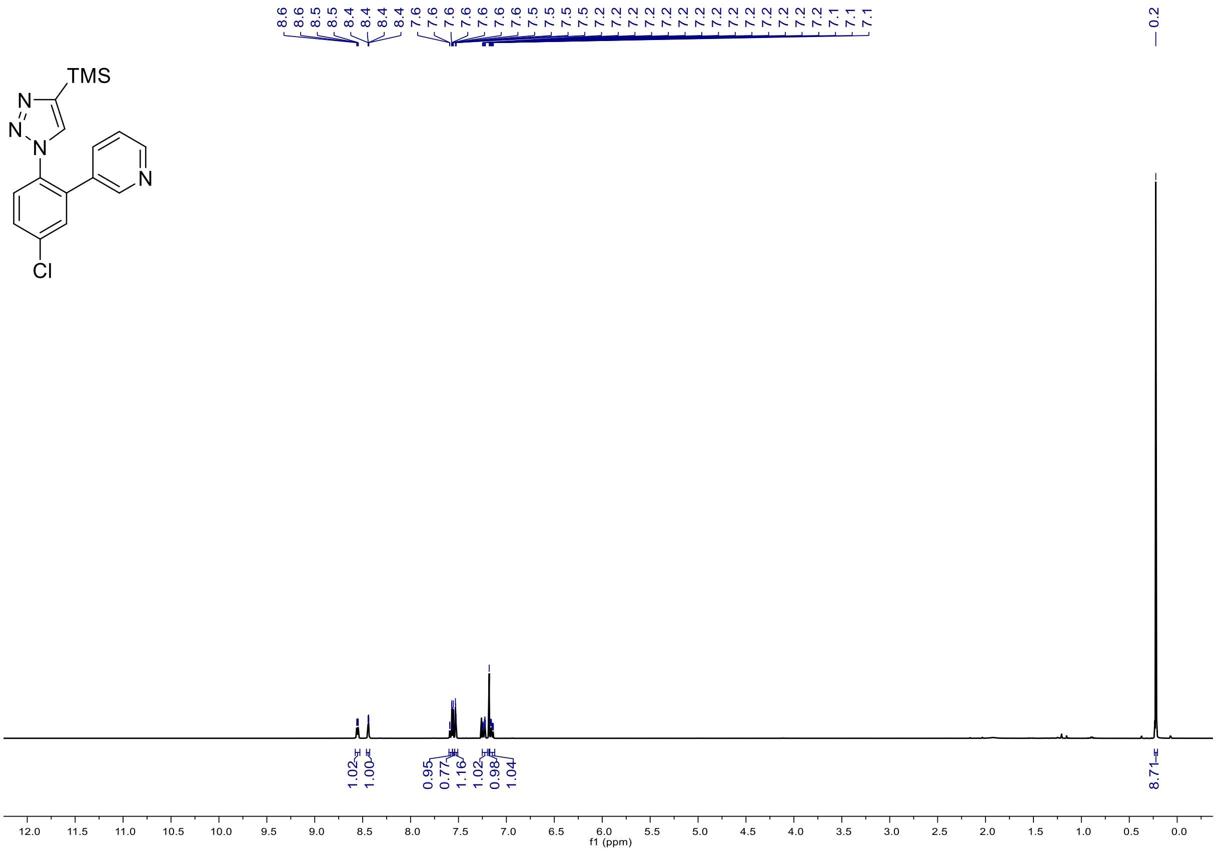

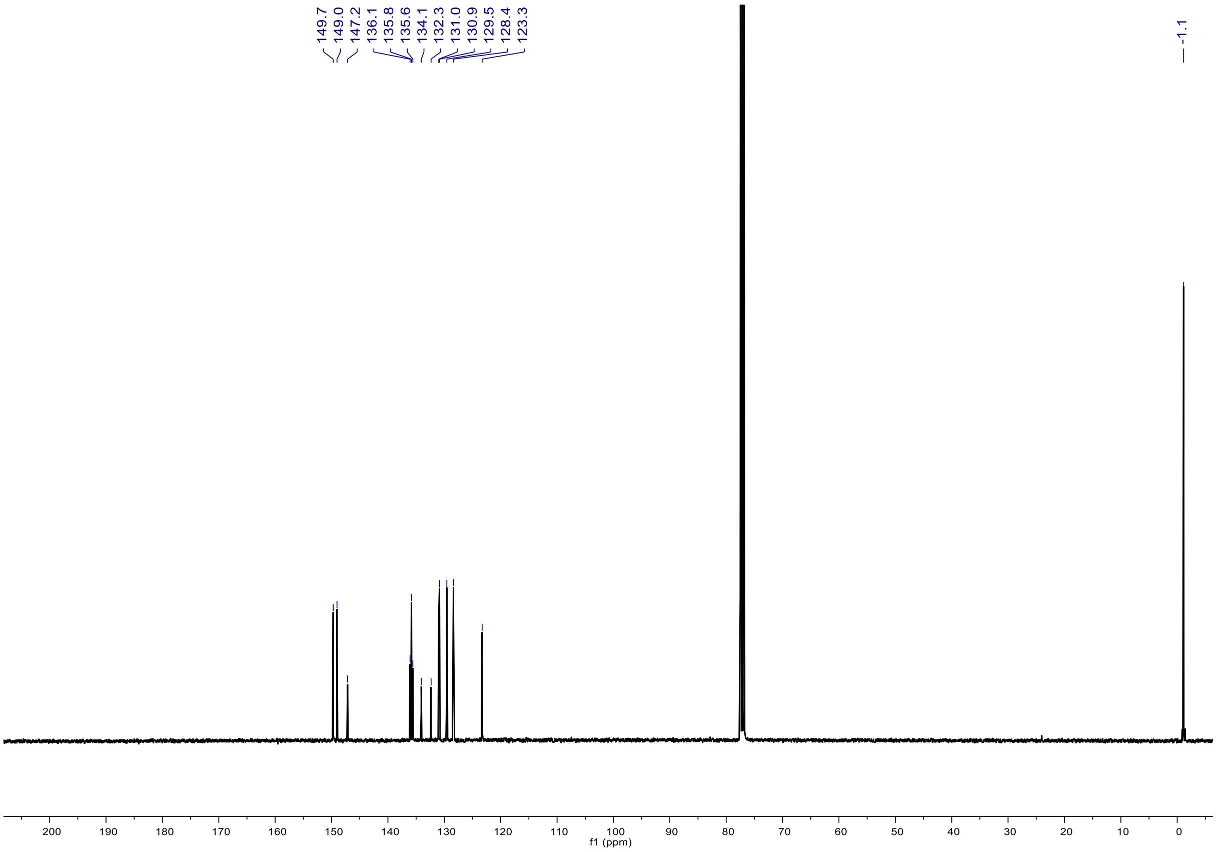

Supplementary Figure 32: ^{1}H-NMR spectrum (top) and ^{13}C-NMR spectrum (bottom) of **1l**.

3-(5-Methyl-2-(4-(trimethylsilyl)-1*H*-1,2,3-triazol-1-yl)phenyl)pyridine (1m)

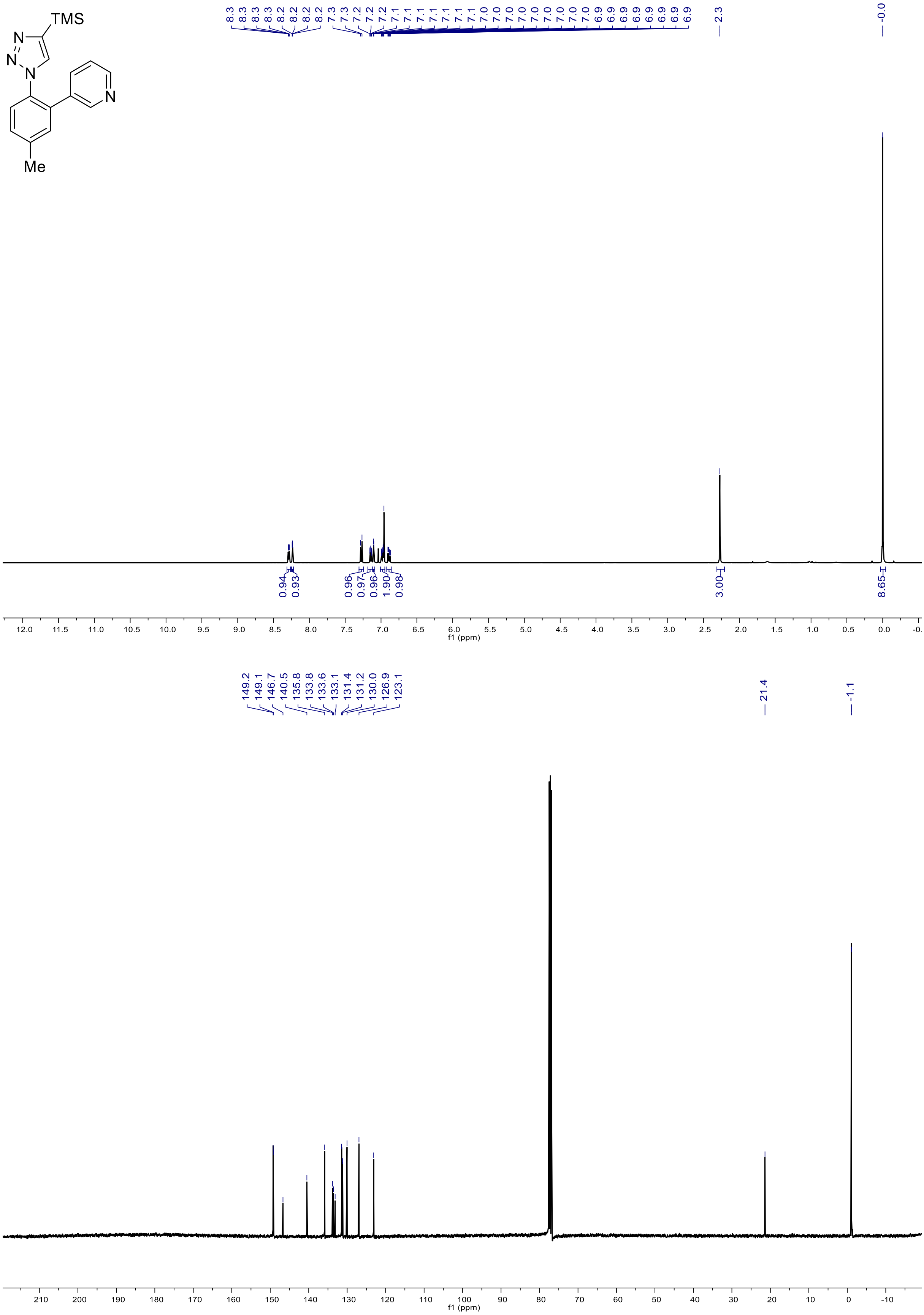

Supplementary Figure 33: ^{1}H-NMR spectrum (top) and ^{13}C-NMR spectrum (bottom) of **1m**.

(5-Methyl-2-(4-(trimethylsilyl)-1*H*-1,2,3-triazol-1-yl)phenyl)magnesium reagent (1n)

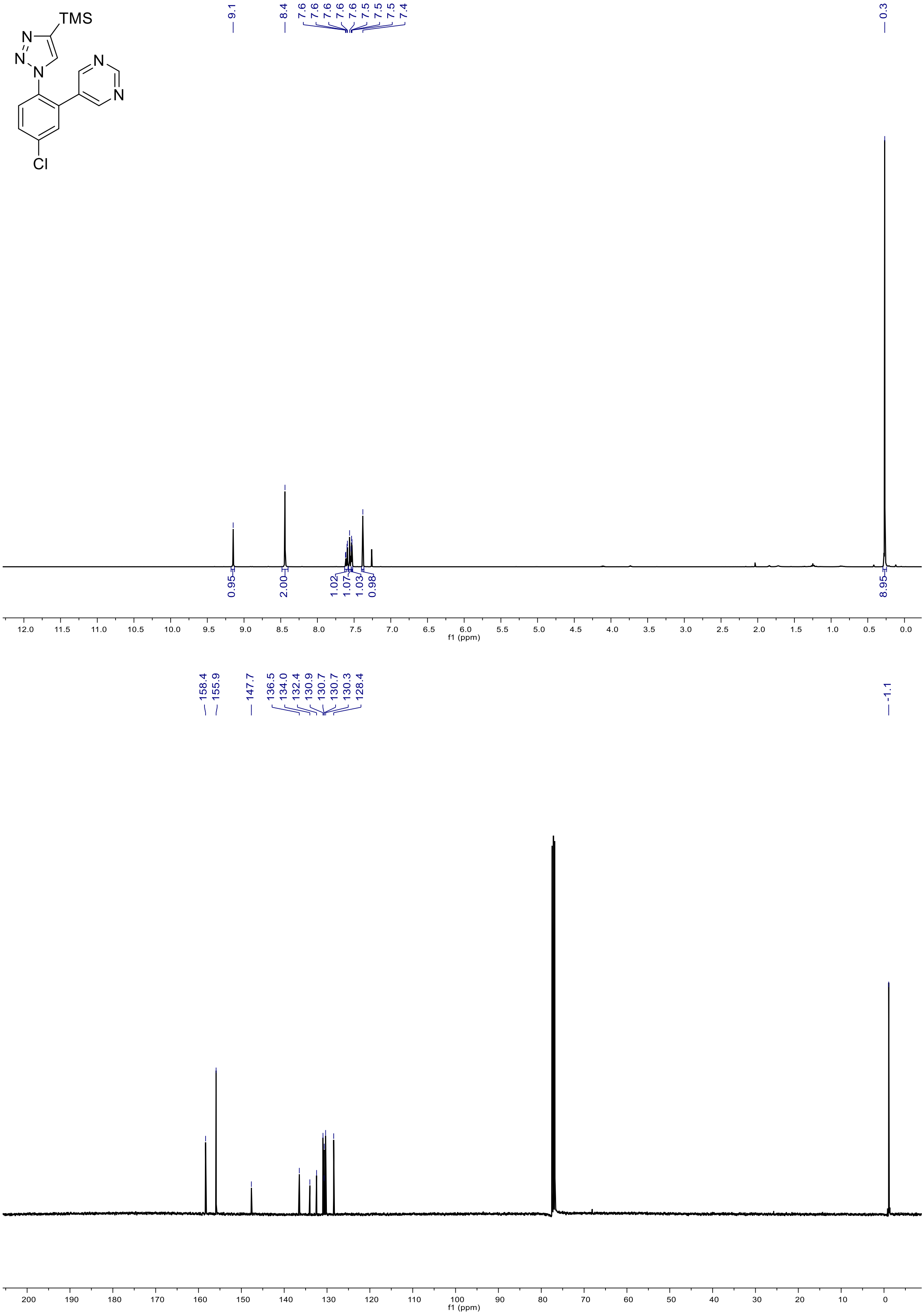

Supplementary Figure 34: ^{1}H-NMR spectrum (top) and ^{13}C-NMR spectrum (bottom) of **1n**.

5-(2-Methoxy-6-(4-(trimethylsilyl)-1*H*-1,2,3-triazol-1-yl)phenyl)-1-methyl-1*H*-indole (1o)

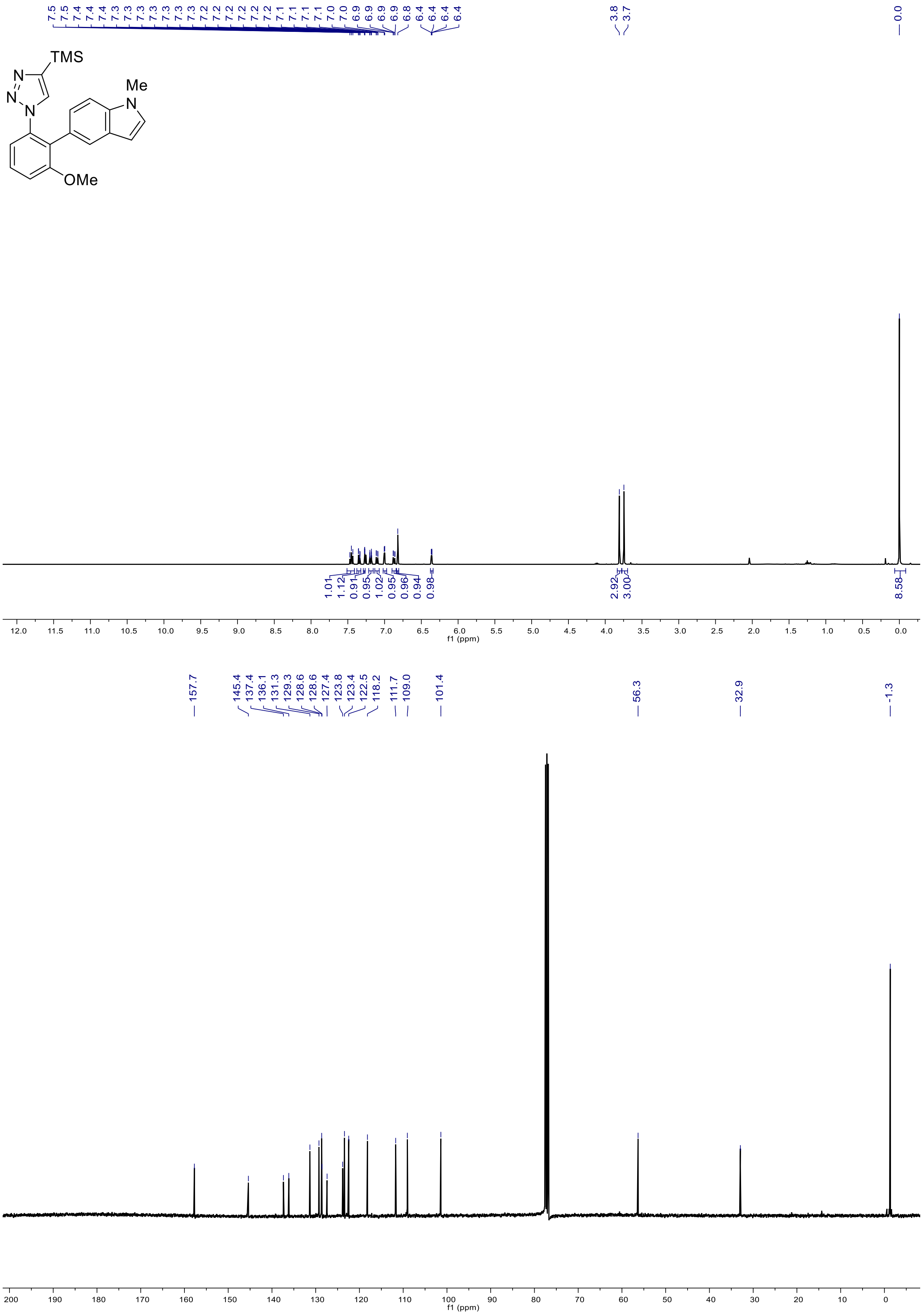

Supplementary Figure 35: ^{1}H-NMR spectrum (top) and ^{13}C-NMR spectrum (bottom) of **1o**.

1-(2-(Benzo[*b*]thiophen-5-yl)-4-methoxyphenyl)-4-(trimethylsilyl)-1*H*-1,2,3-triazol (1p)

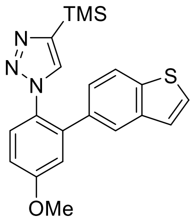

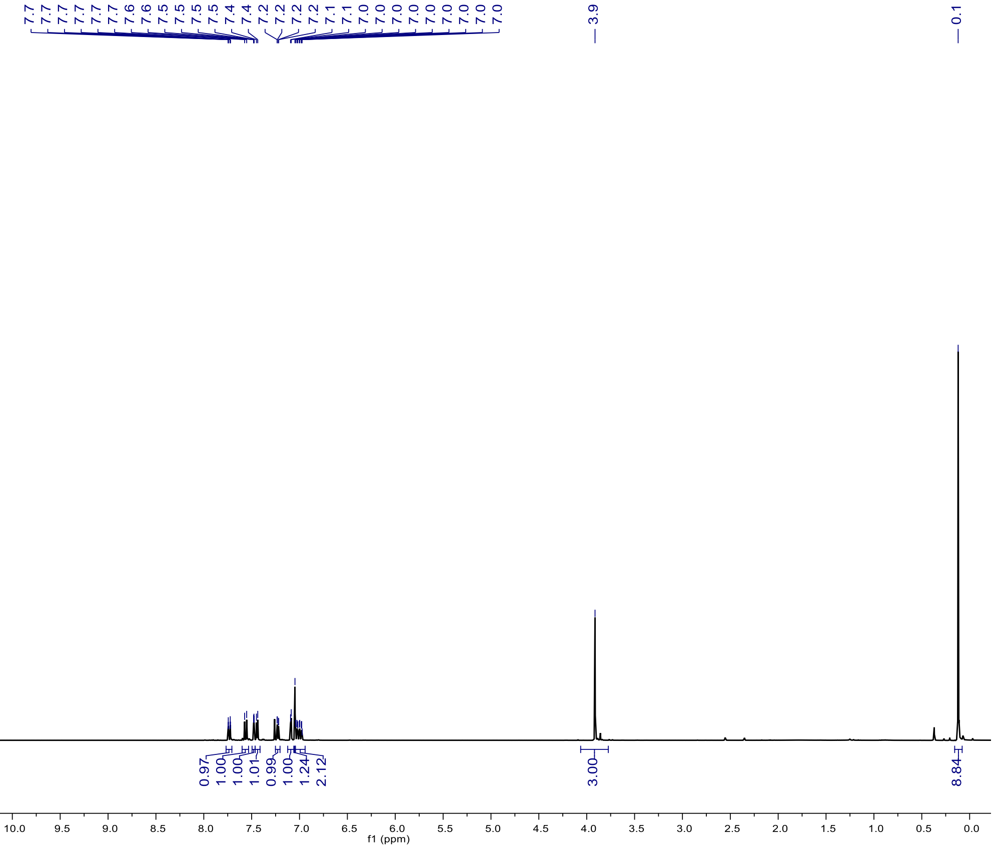

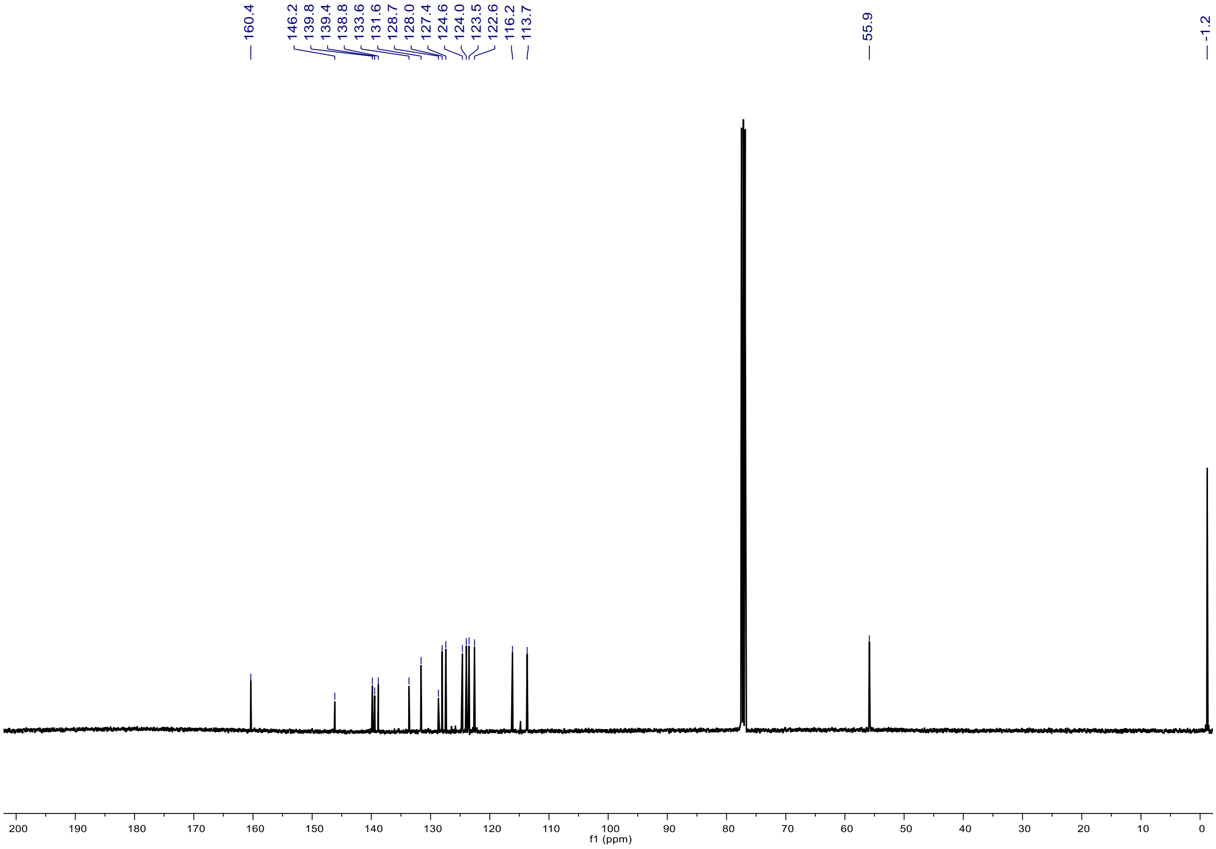

Supplementary Figure 36: ^{1}H-NMR spectrum (top) and ^{13}C-NMR spectrum (bottom) of **1p**.

1-(2-(Thiophen-3-yl)phenyl)-4-(trimethylsilyl)-1*H*-1,2,3-triazole (1q)

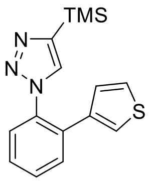

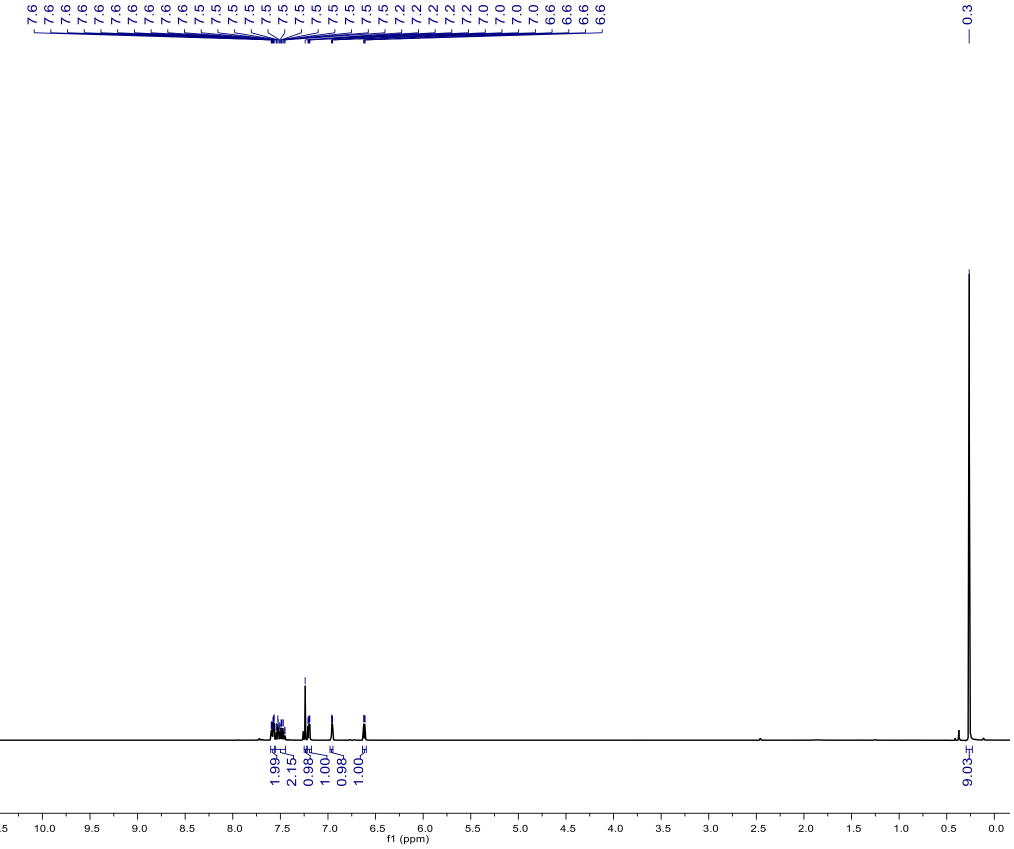

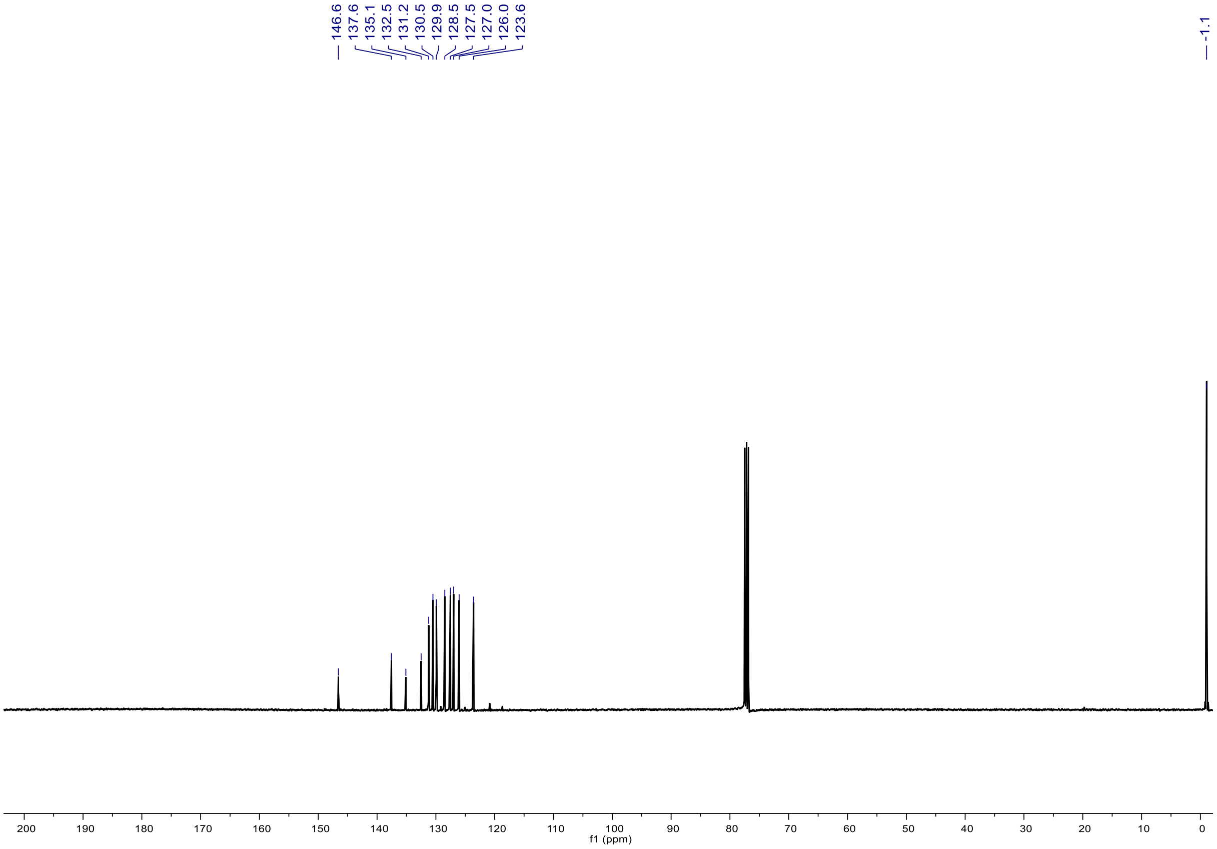

Supplementary Figure 37: ^{1}H-NMR spectrum (top) and ^{13}C-NMR spectrum (bottom) of **1q**).

1-(4-Chloro-2-(thiophen-3-yl)phenyl)-4-(trimethylsilyl)-1*H*-1,2,3-triazole (1r)

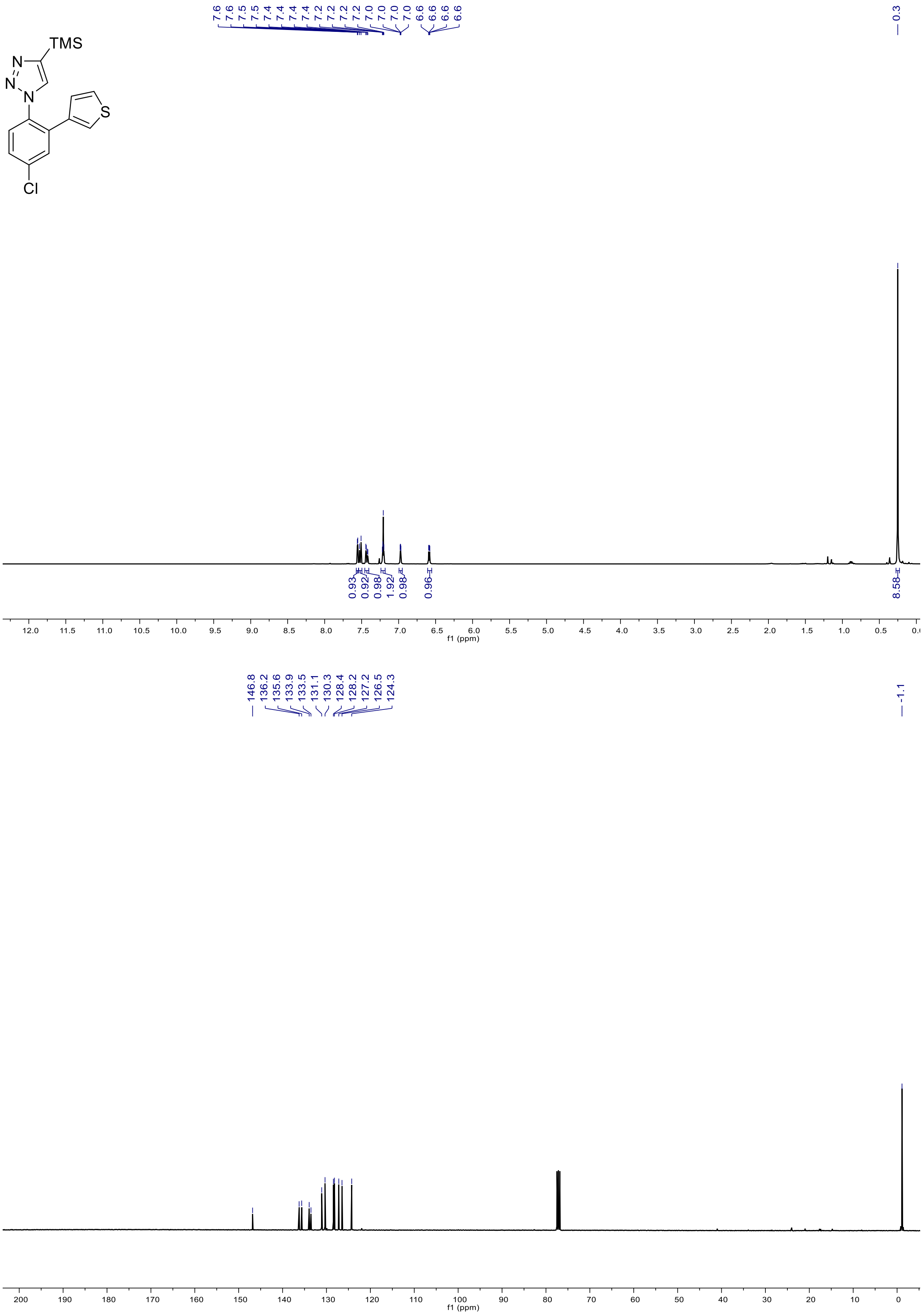

Supplementary Figure 38: ^{1}H-NMR spectrum (top) and ^{13}C-NMR spectrum (bottom) of **1r**.

Ethyl 5-(5-chloro-2-(4-(trimethylsilyl)-1H-1,2,3-triazol-1-yl)phenyl)furan-2-carboxylate (1s)

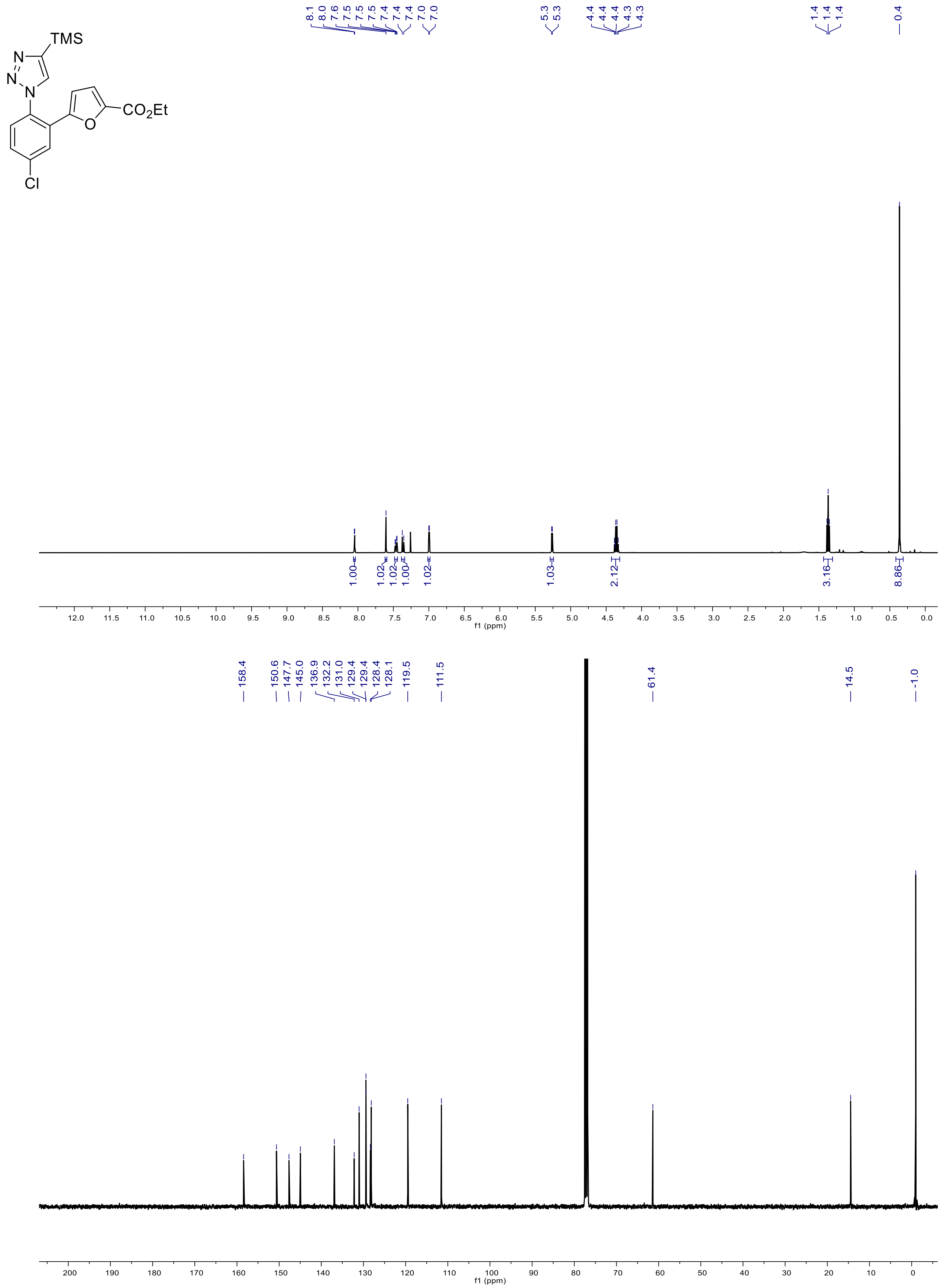

Supplementary Figure 39: ^{1}H-NMR spectrum (top) and ^{13}C-NMR spectrum (bottom) of **1s**.

1-(2-(Benzo[*d*][1,3]dioxol-5-yl)-4-chlorophenyl)-3,5-dimethyl-1*H*-pyrazole (6a)

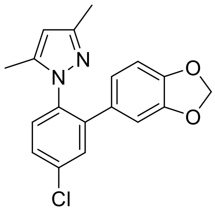

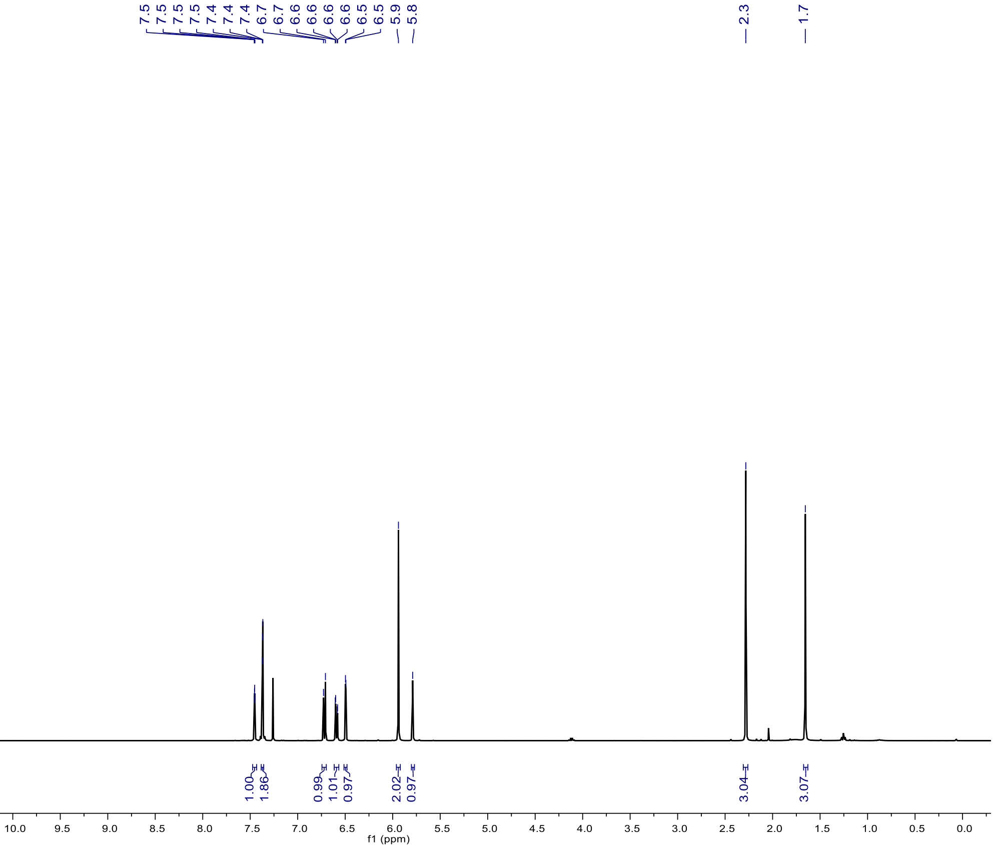

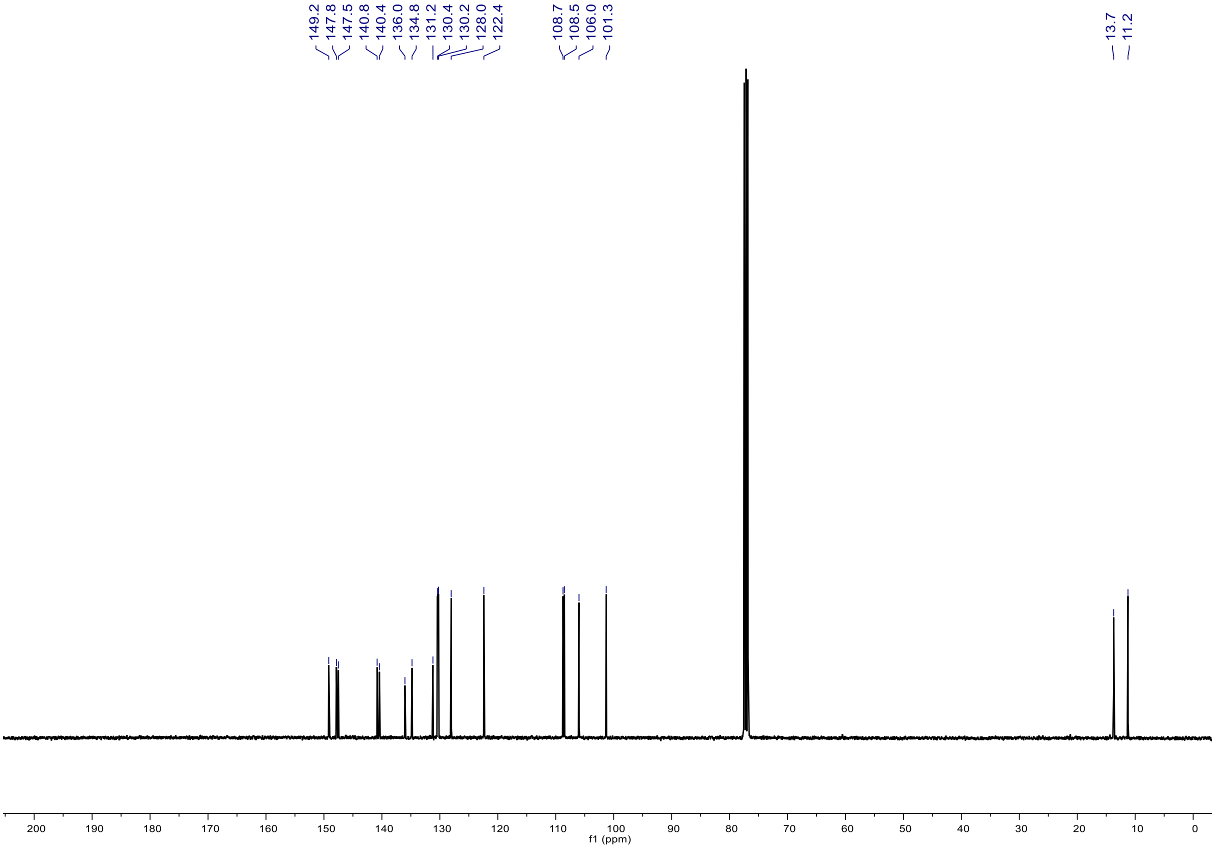

Supplementary Figure 40: ^{1}H-NMR spectrum (top) and ^{13}C-NMR spectrum (bottom) of **6a**.

3-(5-Chloro-2-(3,5-dimethyl-1*H*-pyrazol-1-yl)phenyl)pyridine (6b)

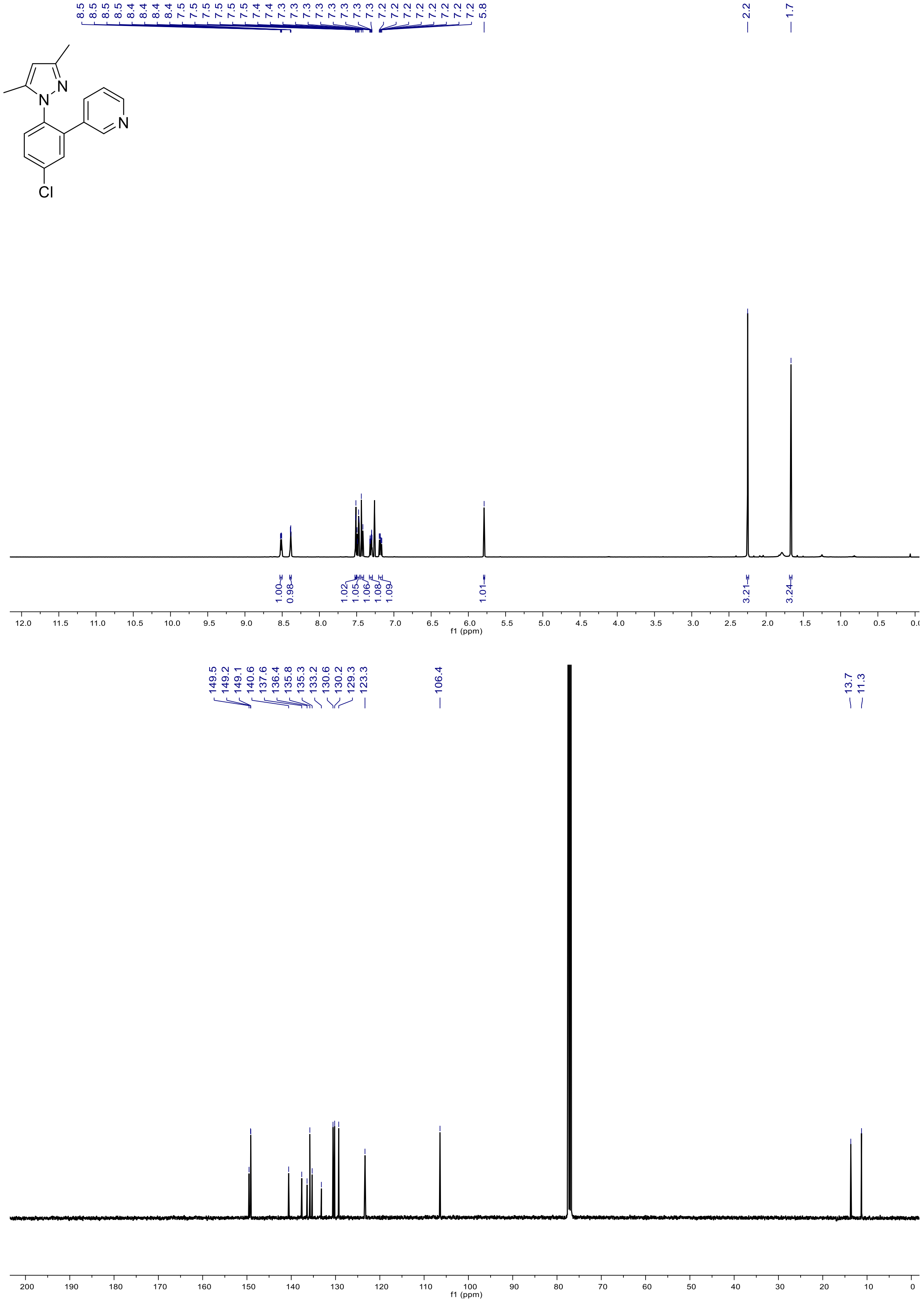

Supplementary Figure 41: ^{1}H-NMR spectrum (top) and ^{13}C-NMR spectrum (bottom) of **6b**.

5'-Chloro-2'-(1*H*-pyrazol-1-yl)-[1,1'-biphenyl]-3-yl 4-methylbenzenesulfonate (6c)

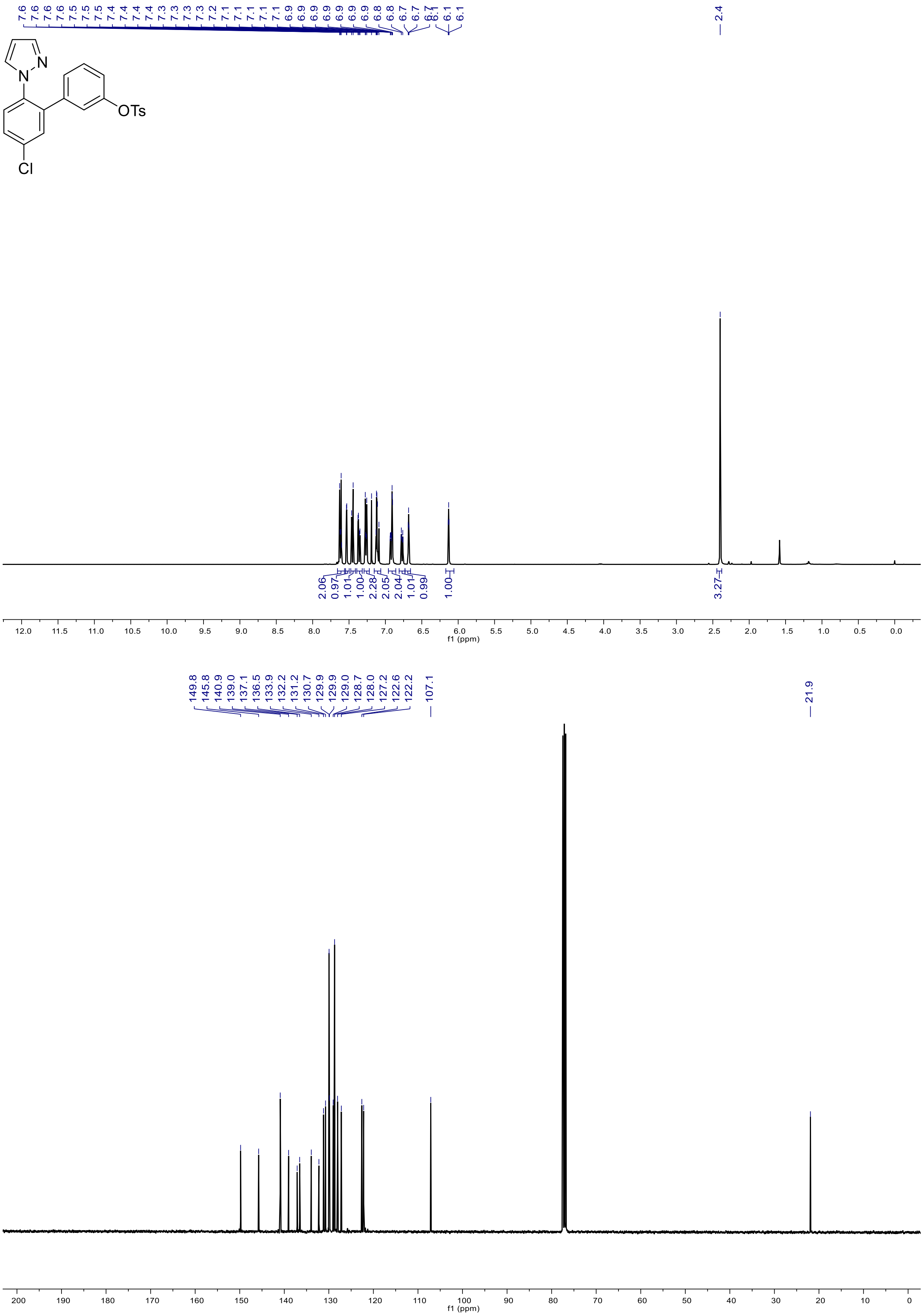

Supplementary Figure 42: ^{1}H-NMR spectrum (top) and ^{13}C-NMR spectrum (bottom) of **6c**.

5'-Chloro-3-fluoro-2'-(1*H*-pyrazol-1-yl)-[1,1'-biphenyl]-4-carbonitrile (6d)

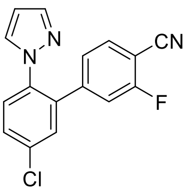

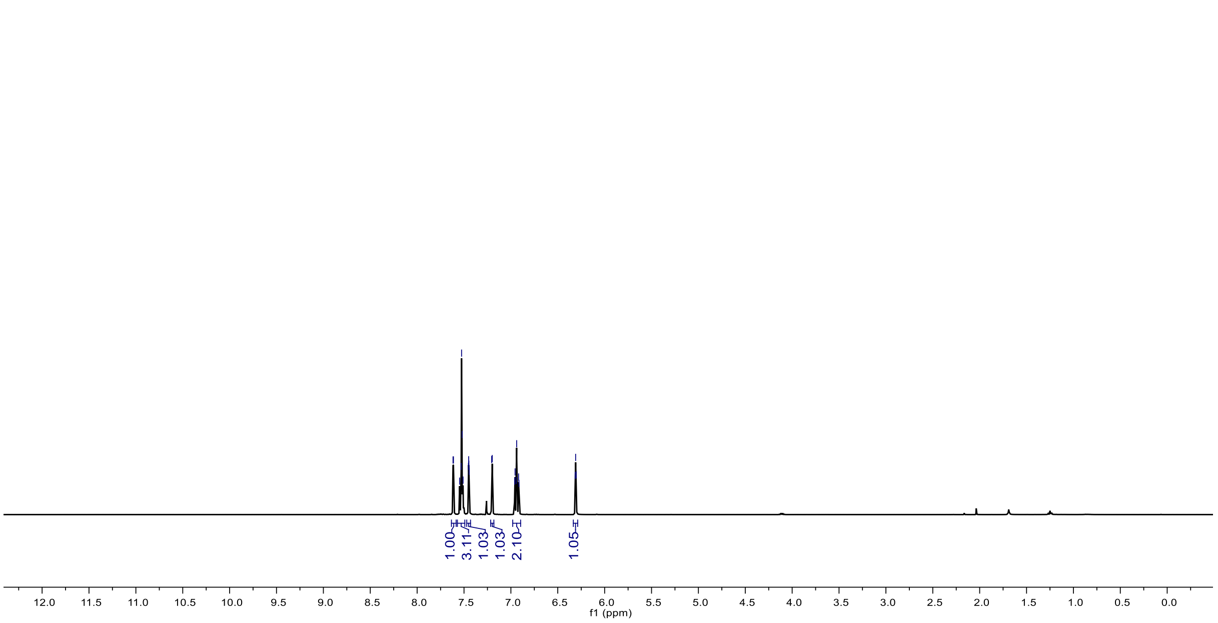

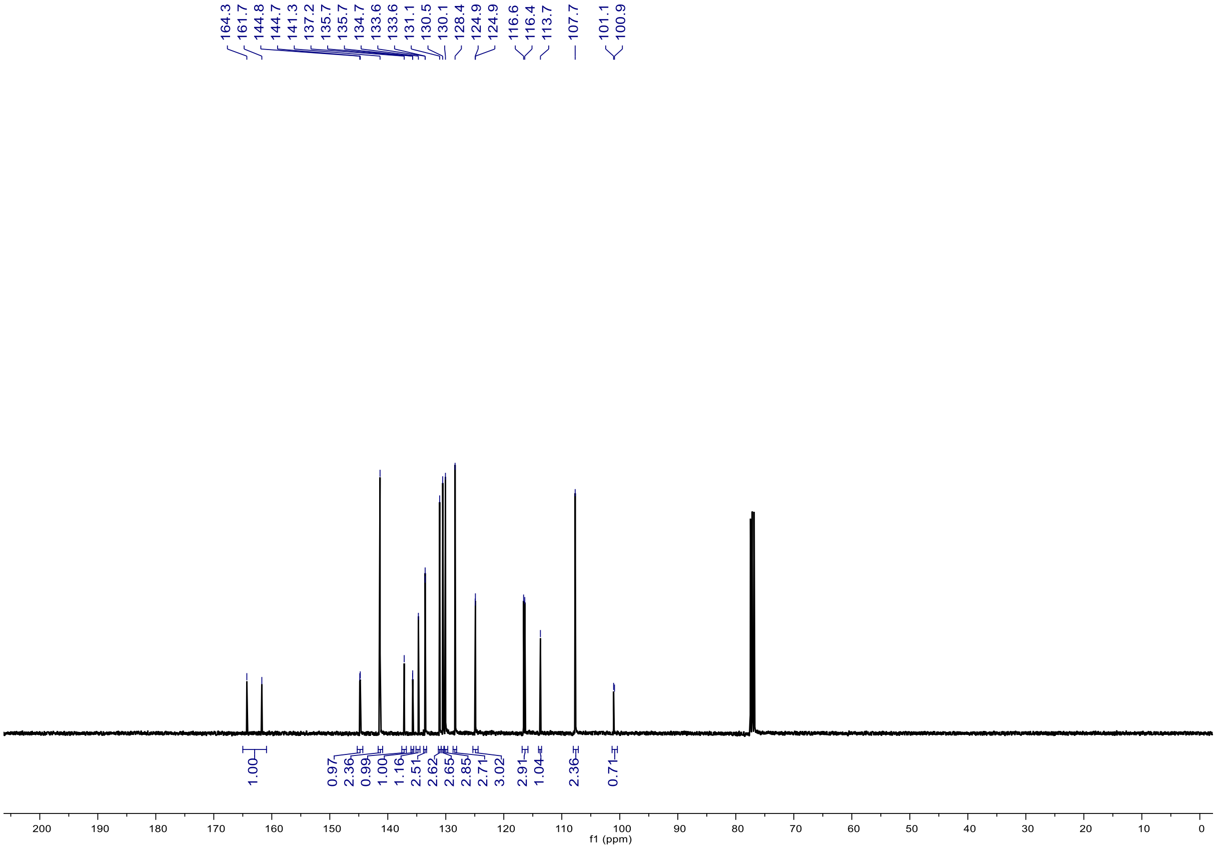

Supplementary Figure 43: ^{1}H-NMR spectrum (top) and ^{13}C-NMR spectrum (bottom) of **6d**.

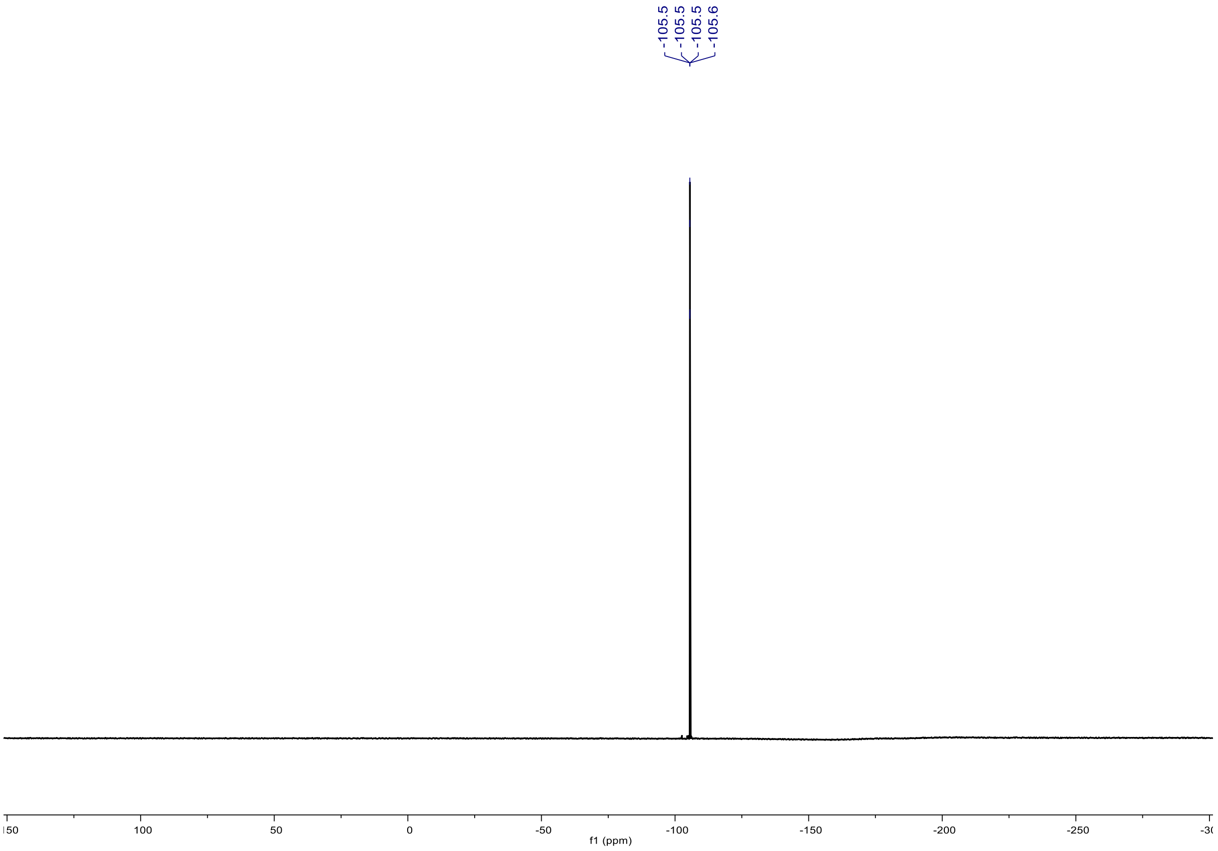

Supplementary Figure 44: ^{19}F-NMR spectrum of **6d**.

2-([1,1'-Biphenyl]-2-yl)-5-phenyl-1,3,4-oxadiazole (6e)

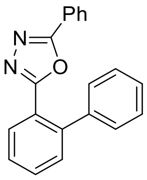

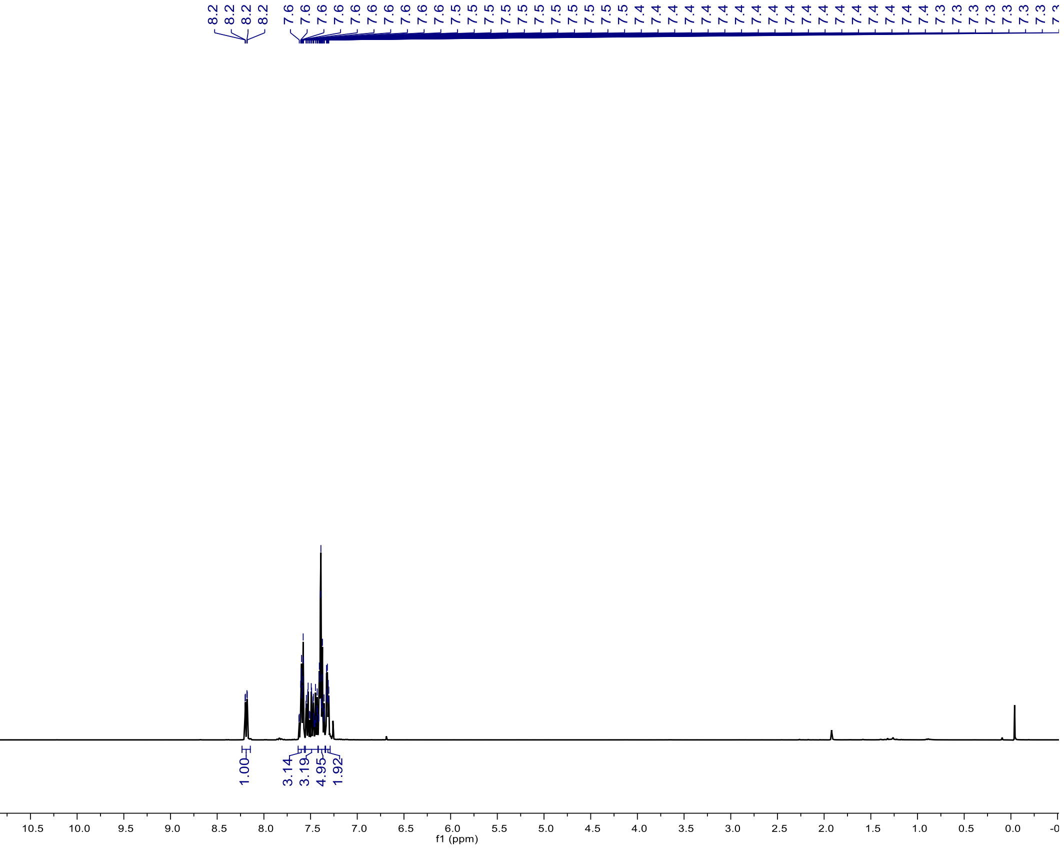

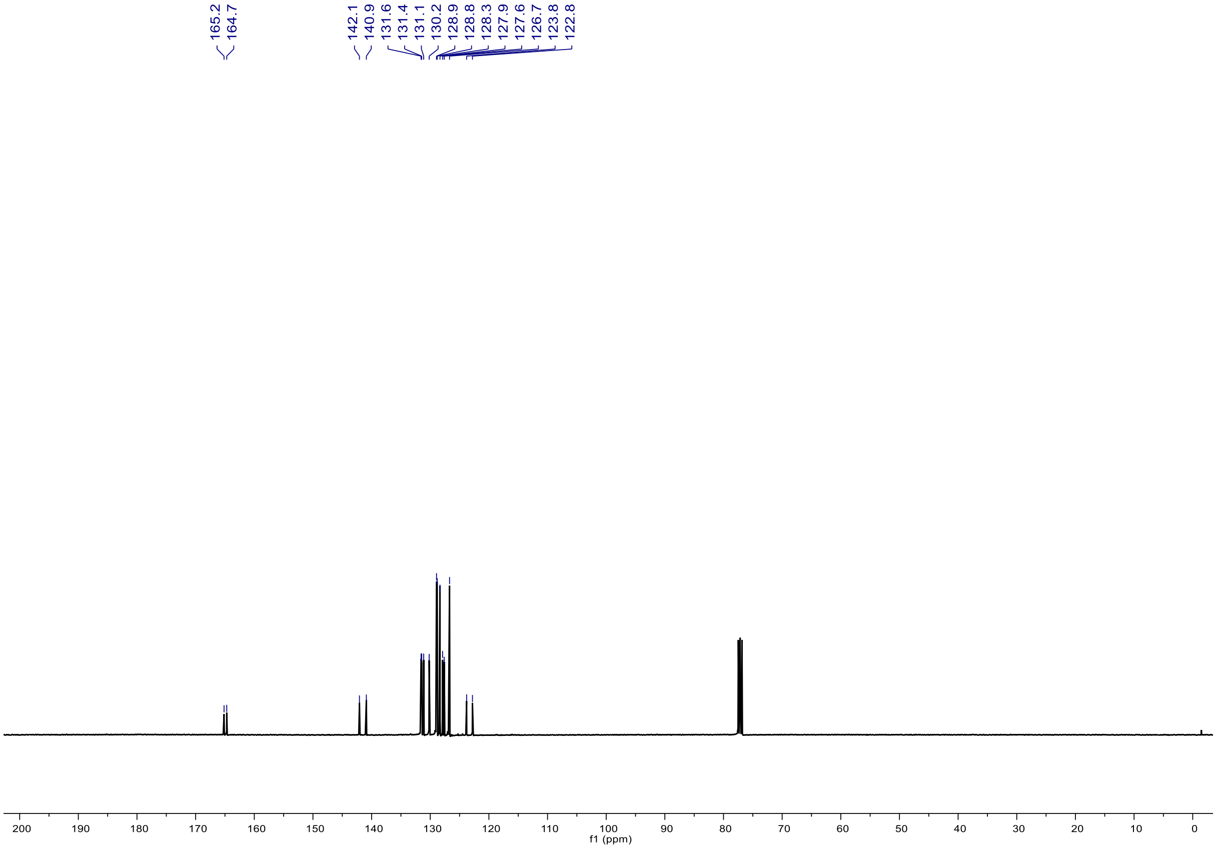

Supplementary Figure 45: ^{1}H-NMR spectrum (top) and ^{13}C-NMR spectrum (bottom) of **6e**.

Ethyl 2'-(5-phenyl-1,3,4-oxadiazol-2-yl)-[1,1'-biphenyl]-3-carboxylate (6f)

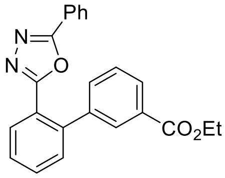

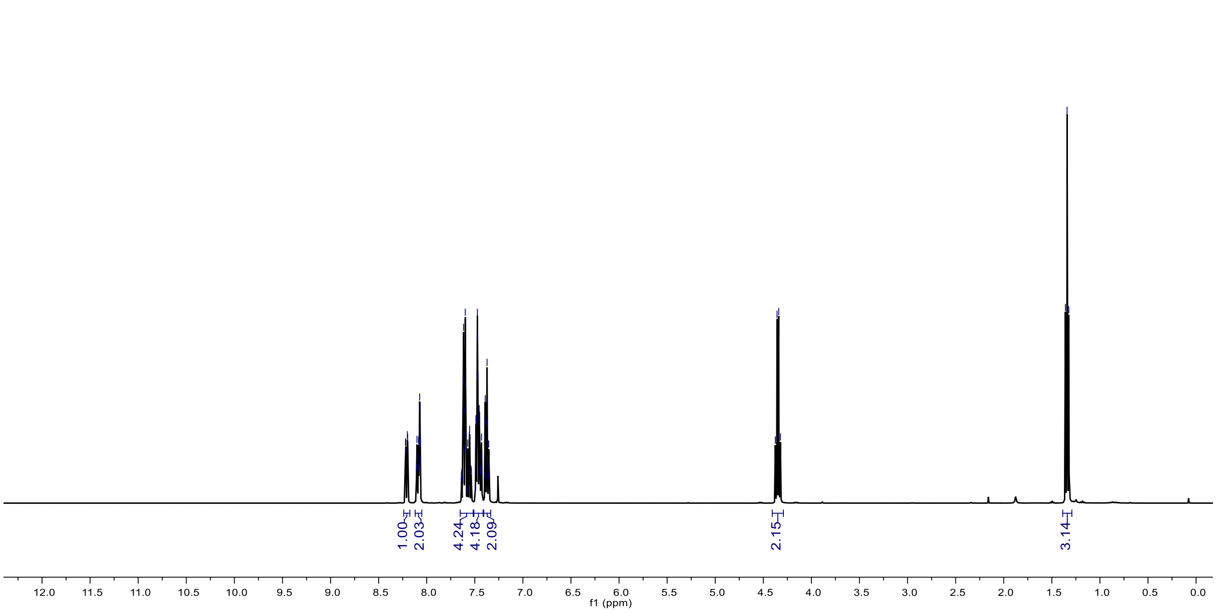

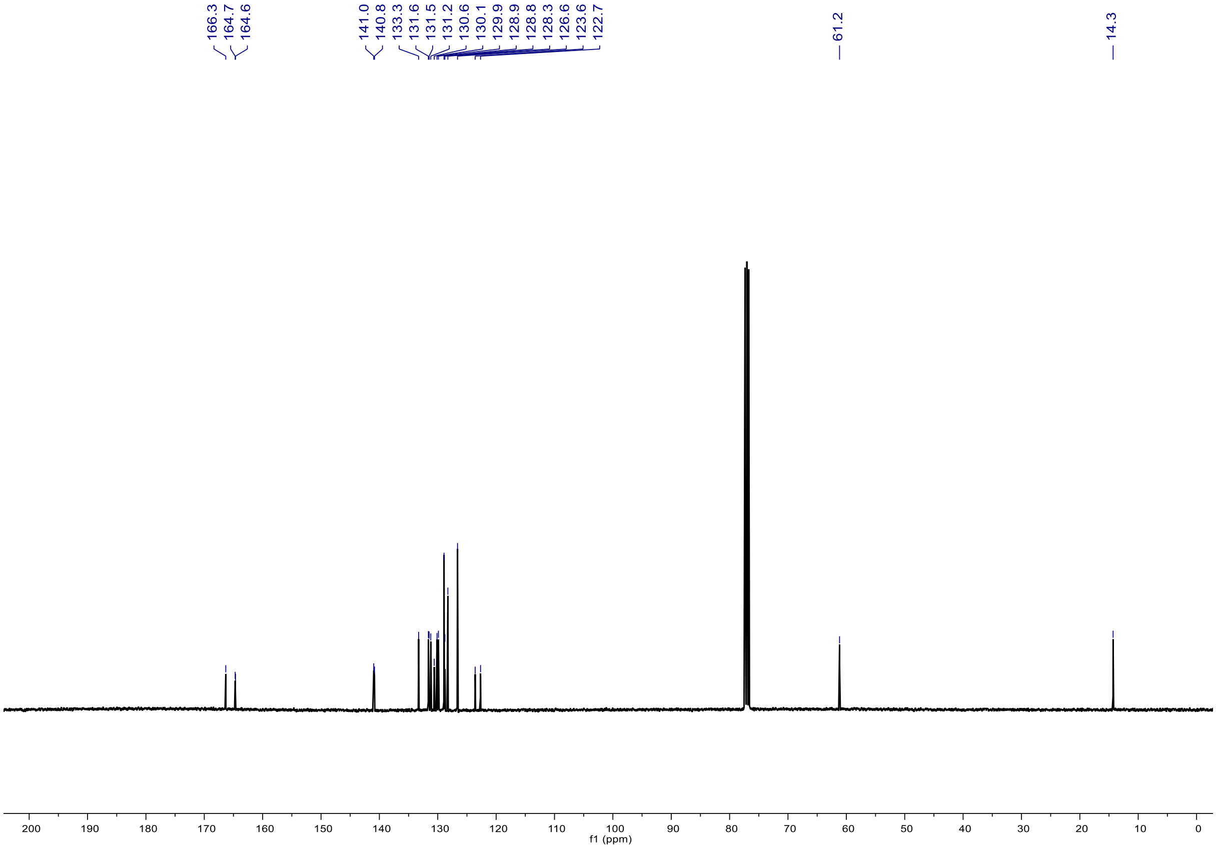

Supplementary Figure 46: ^{1}H-NMR spectrum (top) and ^{13}C-NMR spectrum (bottom) of **6f**.

4,4-Dimethyl-2-(4'-(pentafluoro-λ⁶-sulfaneyl)-[1,1'-biphenyl]-2-yl)-4,5-dihydrooxazole (6g)

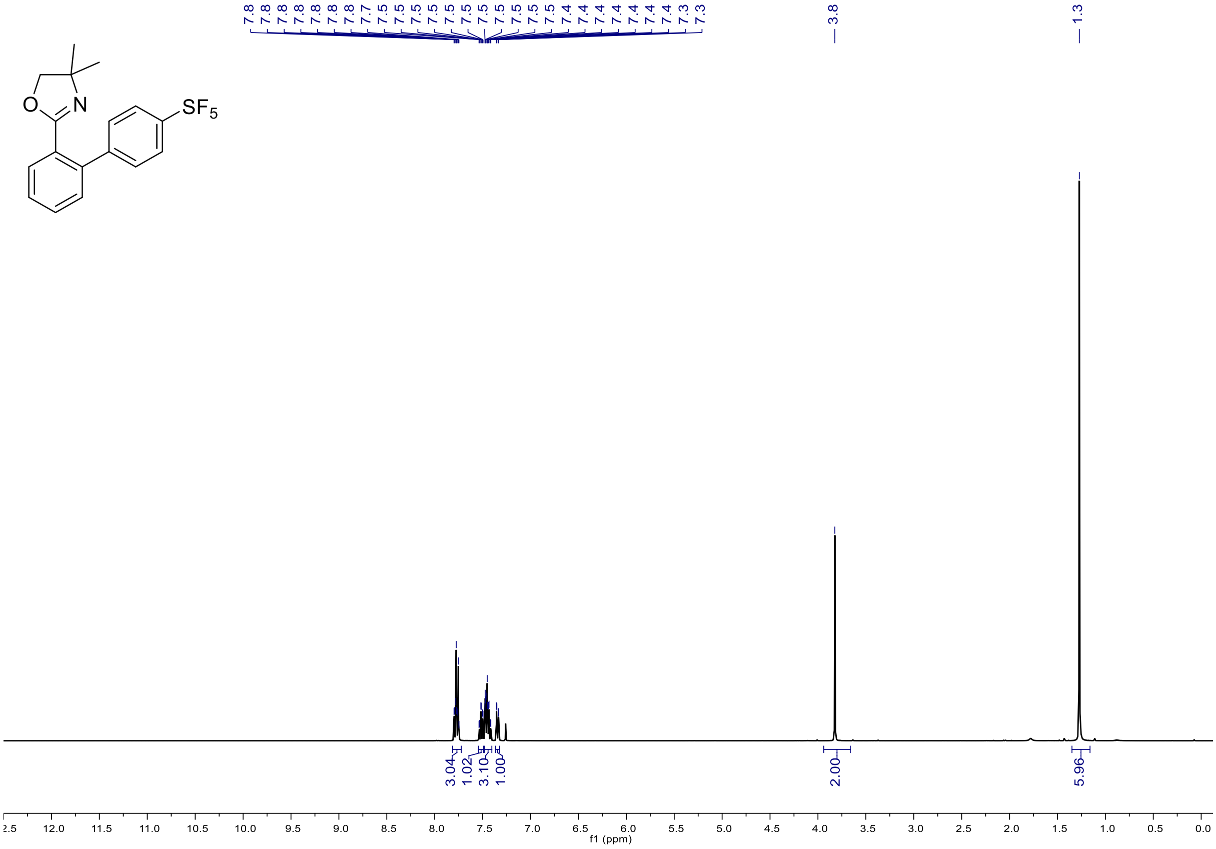

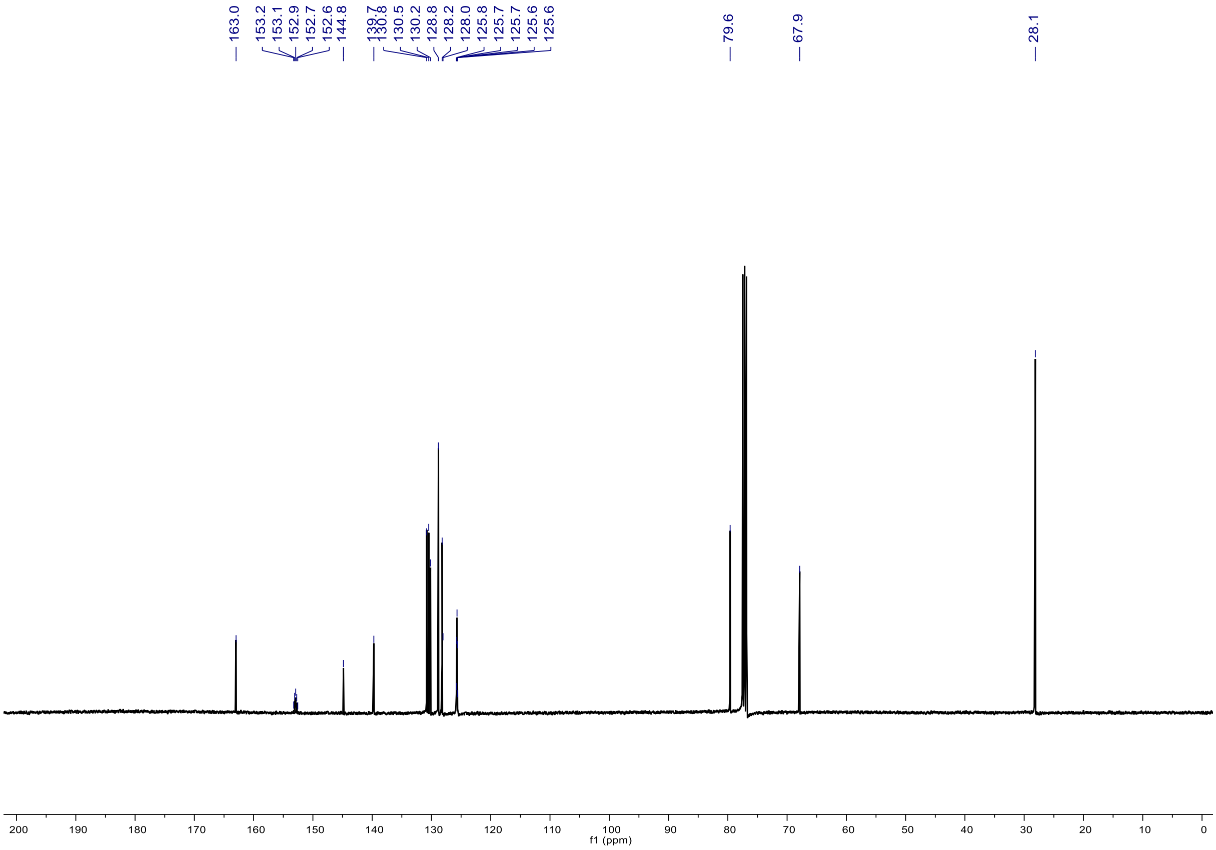

Supplementary Figure 47: ^{1}H-NMR spectrum (top) and ^{13}C-NMR spectrum (bottom) of **6g**.

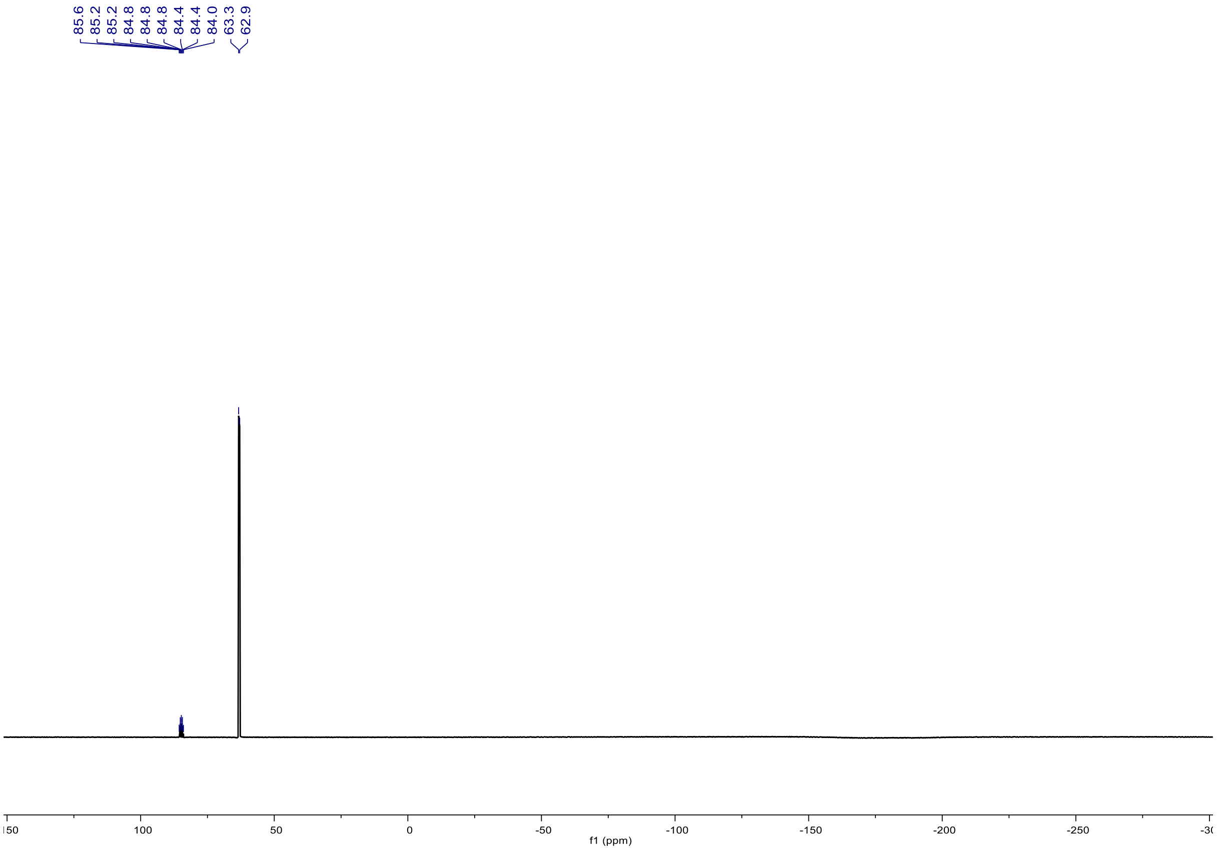

Supplementary Figure 48: ^{19}F-NMR spectrum of **6g**.

4,4-Dimethyl-2-(2-(1-methyl-1*H*-indol-5-yl)phenyl)-4,5-dihydrooxazole (6h)

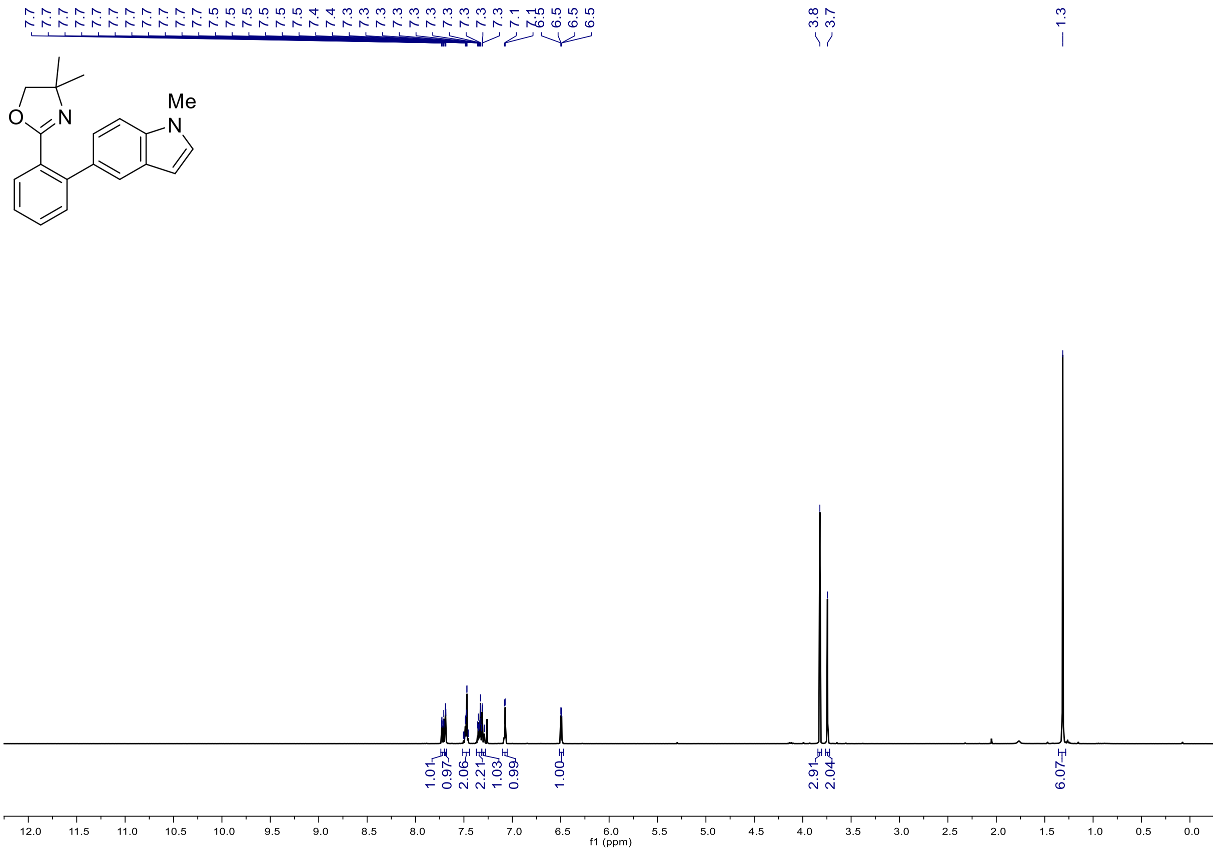

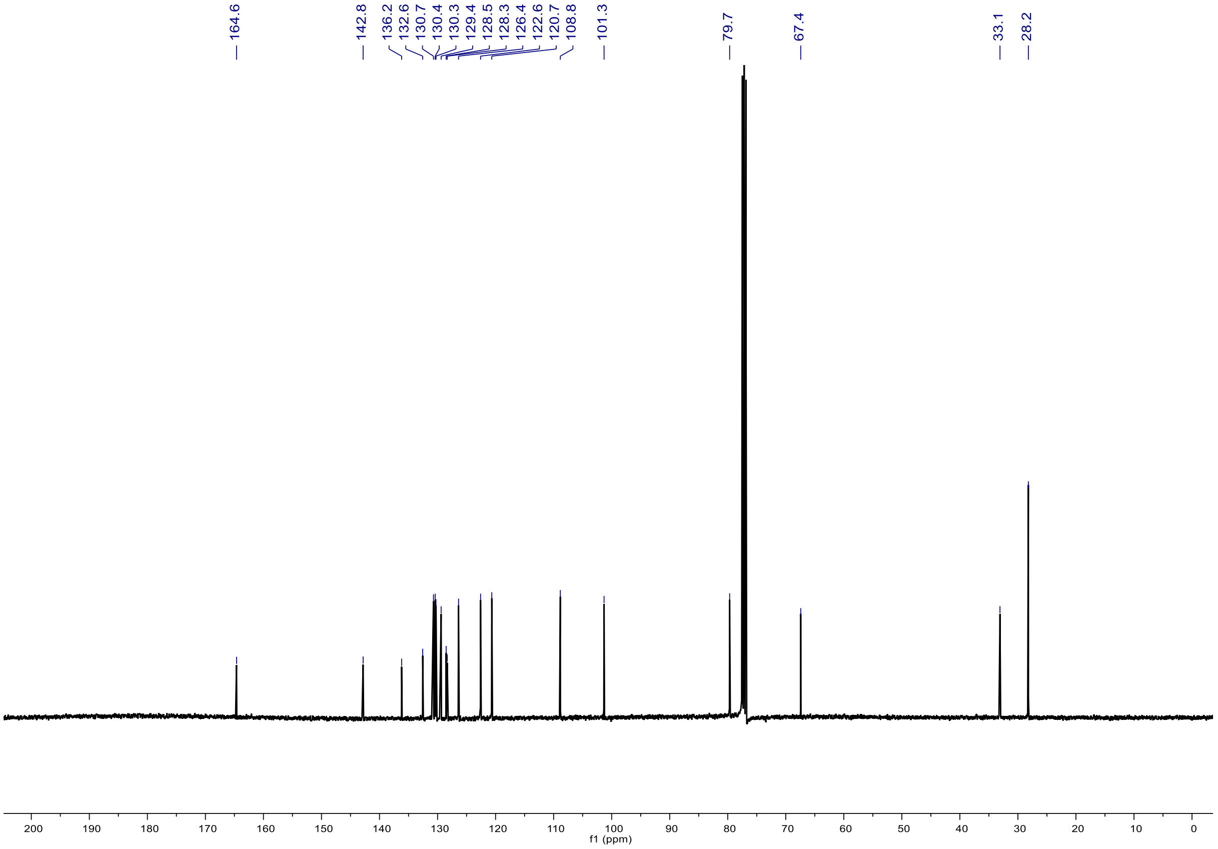

Supplementary Figure 49: ^{1}H-NMR spectrum (top) and ^{13}C-NMR spectrum (bottom) of **6h**.

1-(4-Chloro-2-iodophenyl)-4-(trimethylsilyl)-1*H*-1,2,3-triazole (7a)

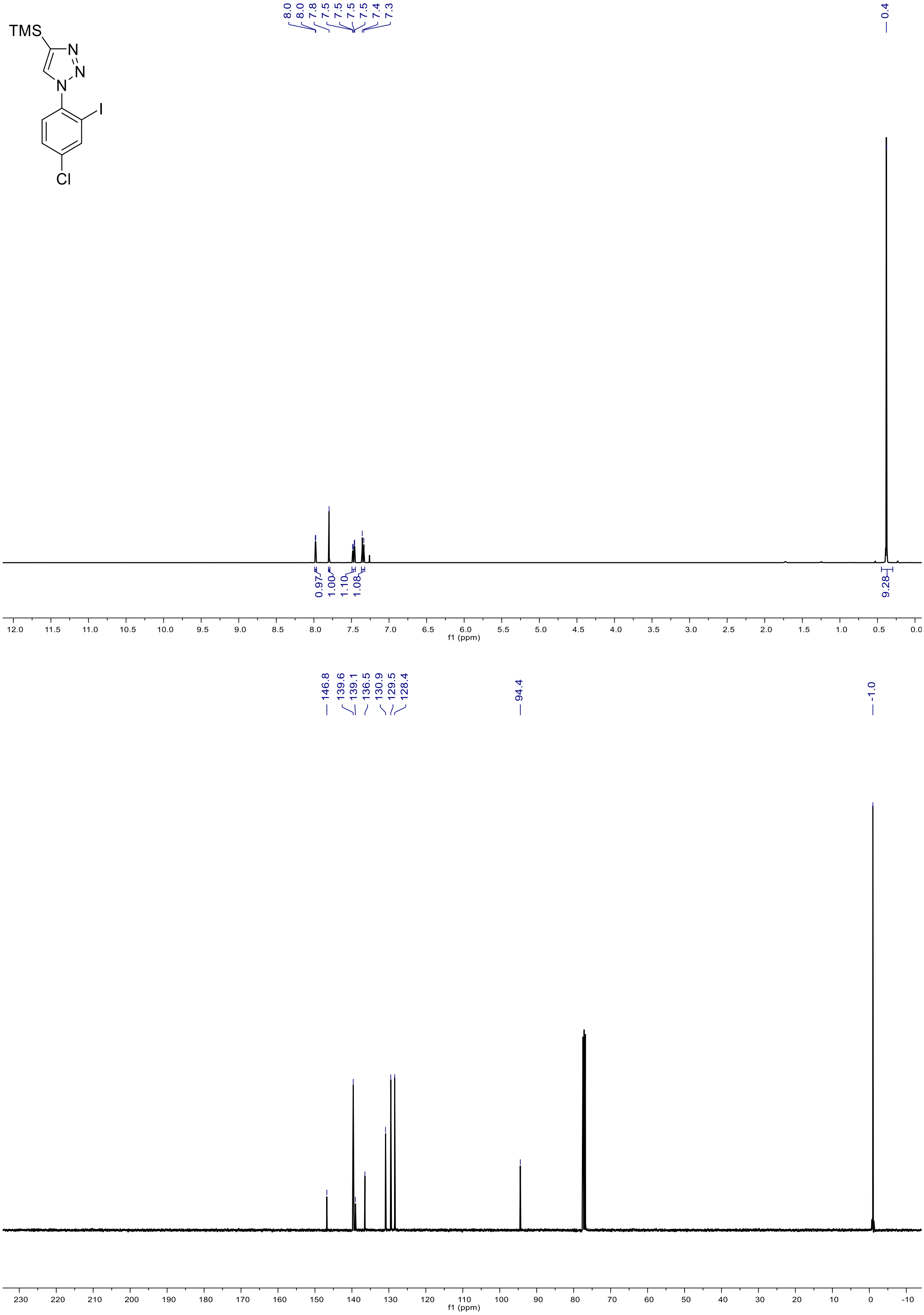

Supplementary Figure 50: ^{1}H-NMR spectrum (top) and ^{13}C-NMR spectrum (bottom) of **7a**.

(5-Chloro-2-(4-(trimethylsilyl)-1*H*-1,2,3-triazol-1-yl)phenyl)(phenyl)methanol (7b)

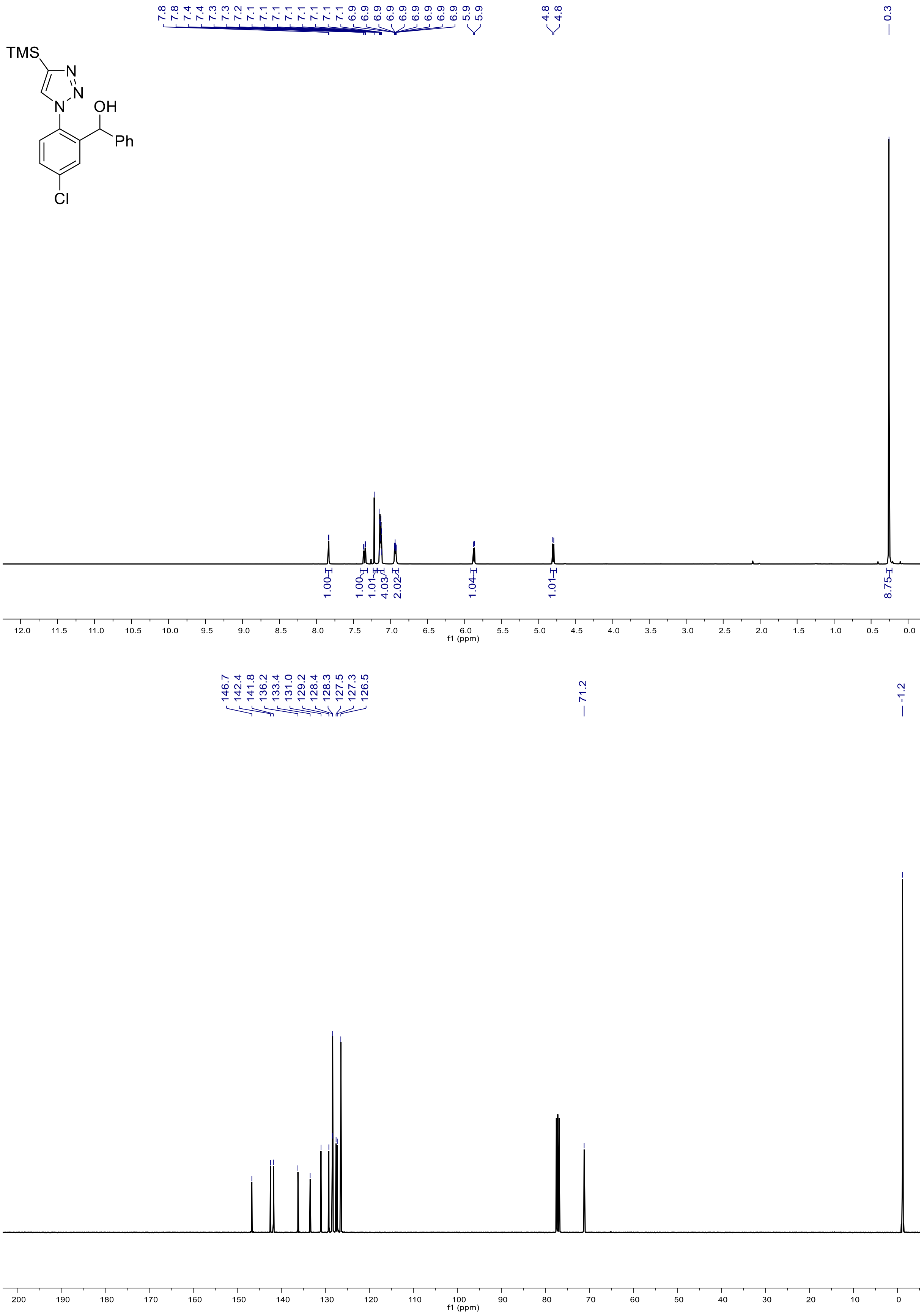

Supplementary Figure 51: ^{1}H-NMR spectrum (top) and ^{13}C-NMR spectrum (bottom) of **7b**.

1-(4-Chloro-2-(methylthio)phenyl)-4-(trimethylsilyl)-1*H*-1,2,3-triazole (7c)

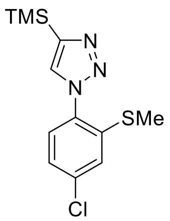

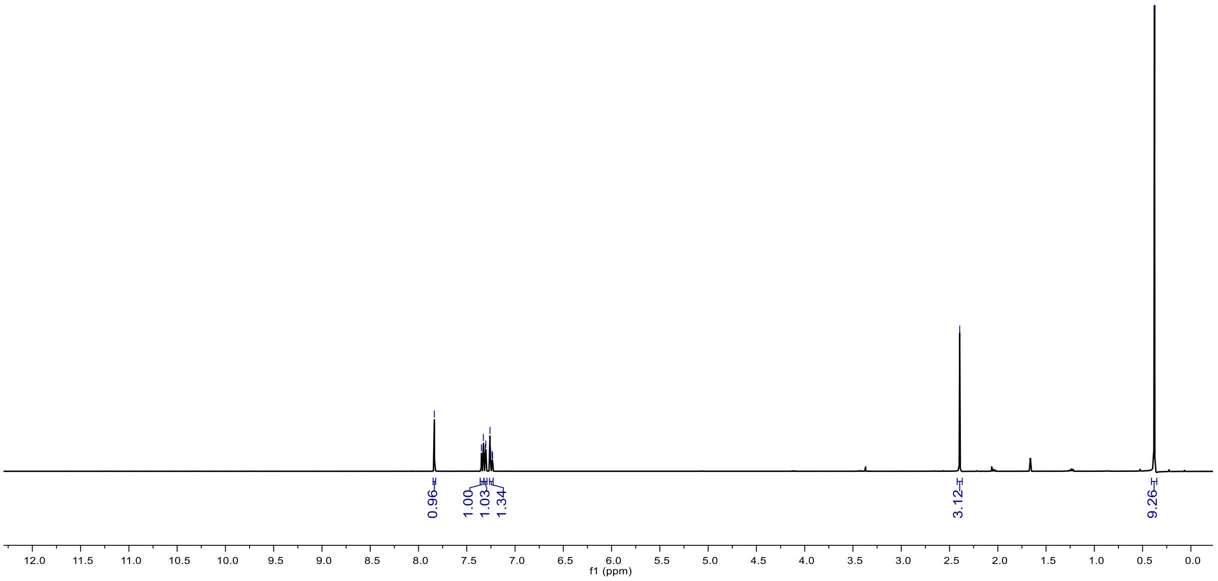

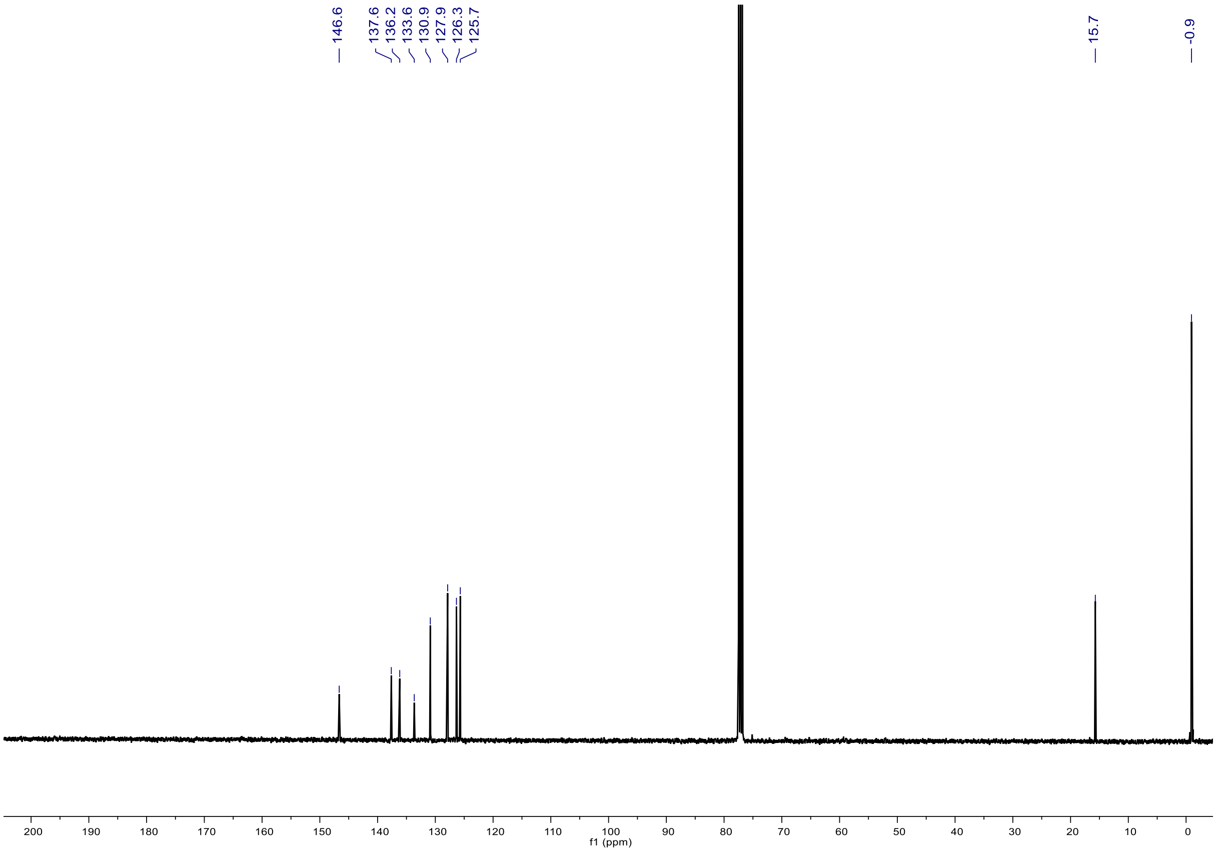

Supplementary Figure 52: ^{1}H-NMR spectrum (top) and ^{13}C-NMR spectrum (bottom) of **7c**.

(5-Chloro-2-(4-(trimethylsilyl)-1*H*-1,2,3-triazol-1-yl)phenyl)(phenyl)methanone (7d)

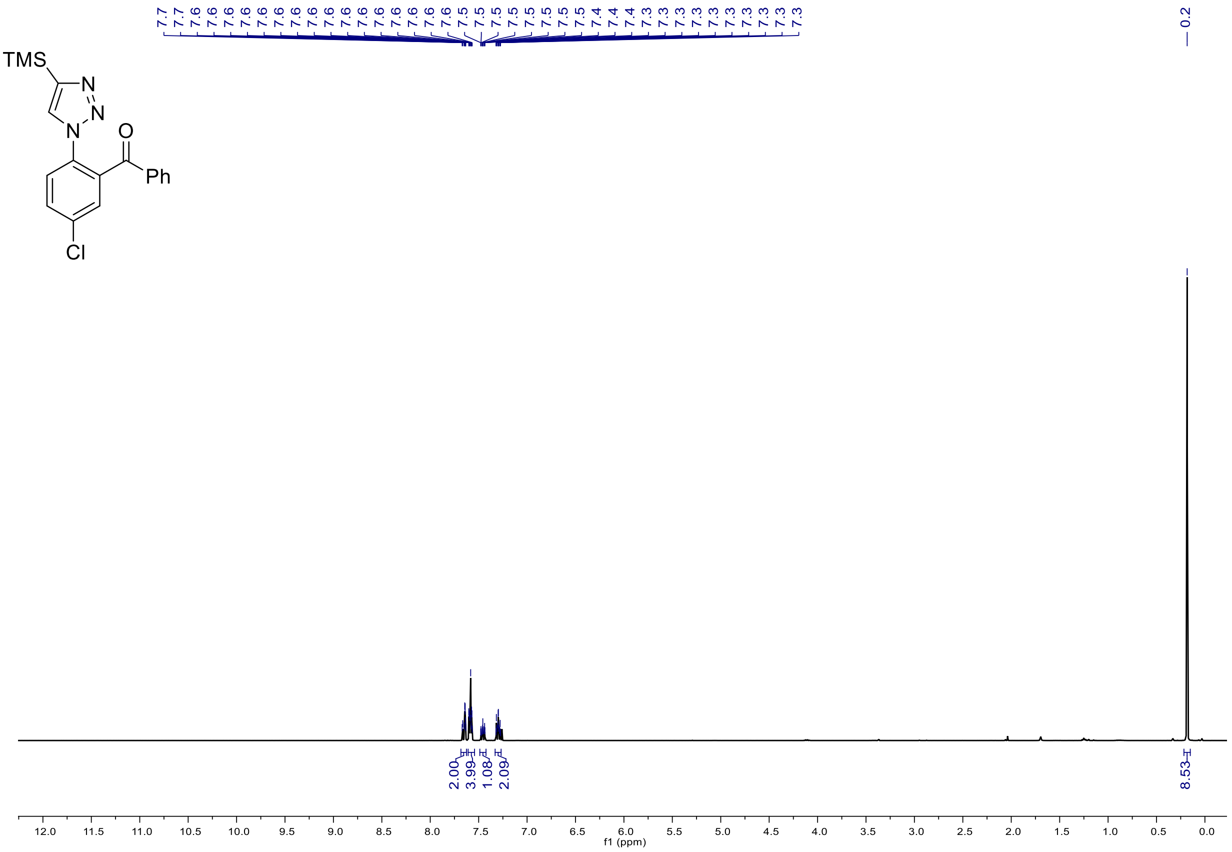

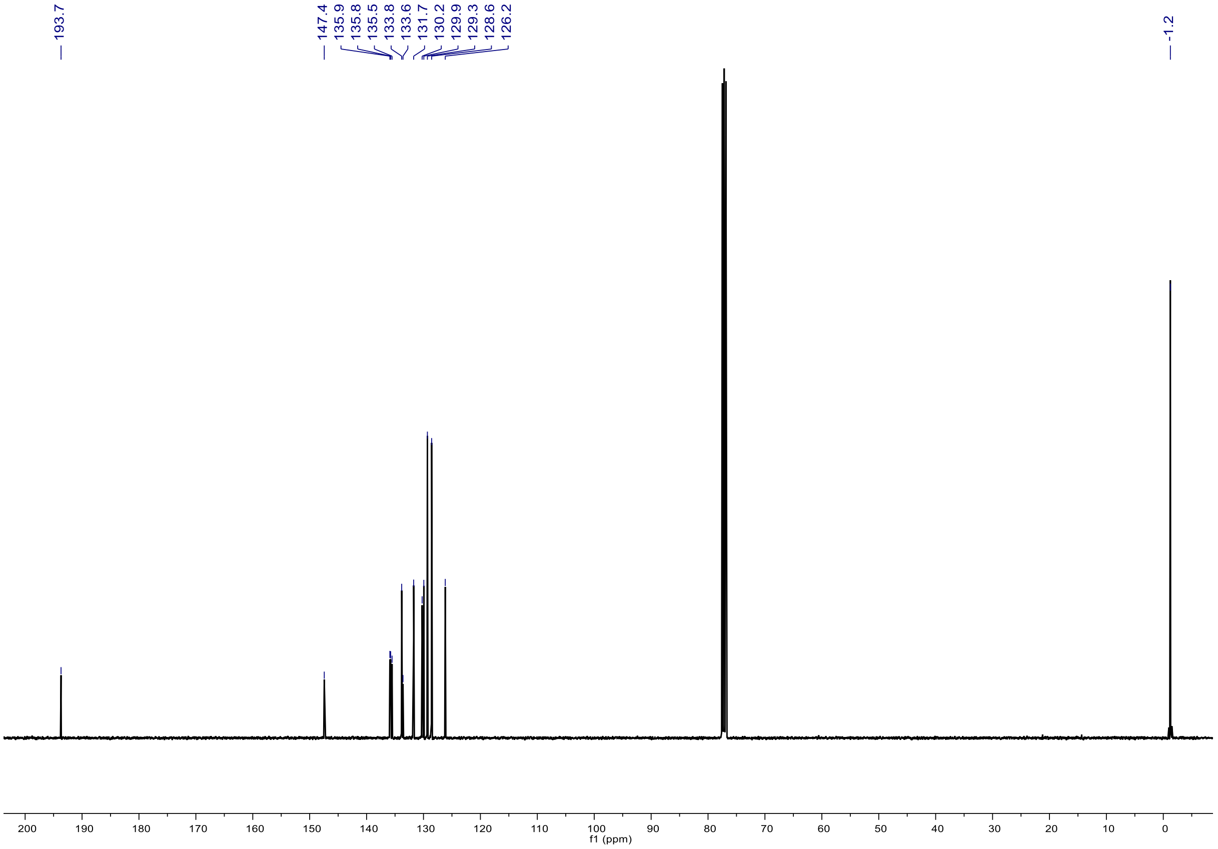

Supplementary Figure 53: ^{1}H-NMR spectrum (top) and ^{13}C-NMR spectrum (bottom) of **7d**.

Ethyl 2-(5-chloro-2-(4-(trimethylsilyl)-1*H*-1,2,3-triazol-1-yl)benzyl)acrylate (7e)

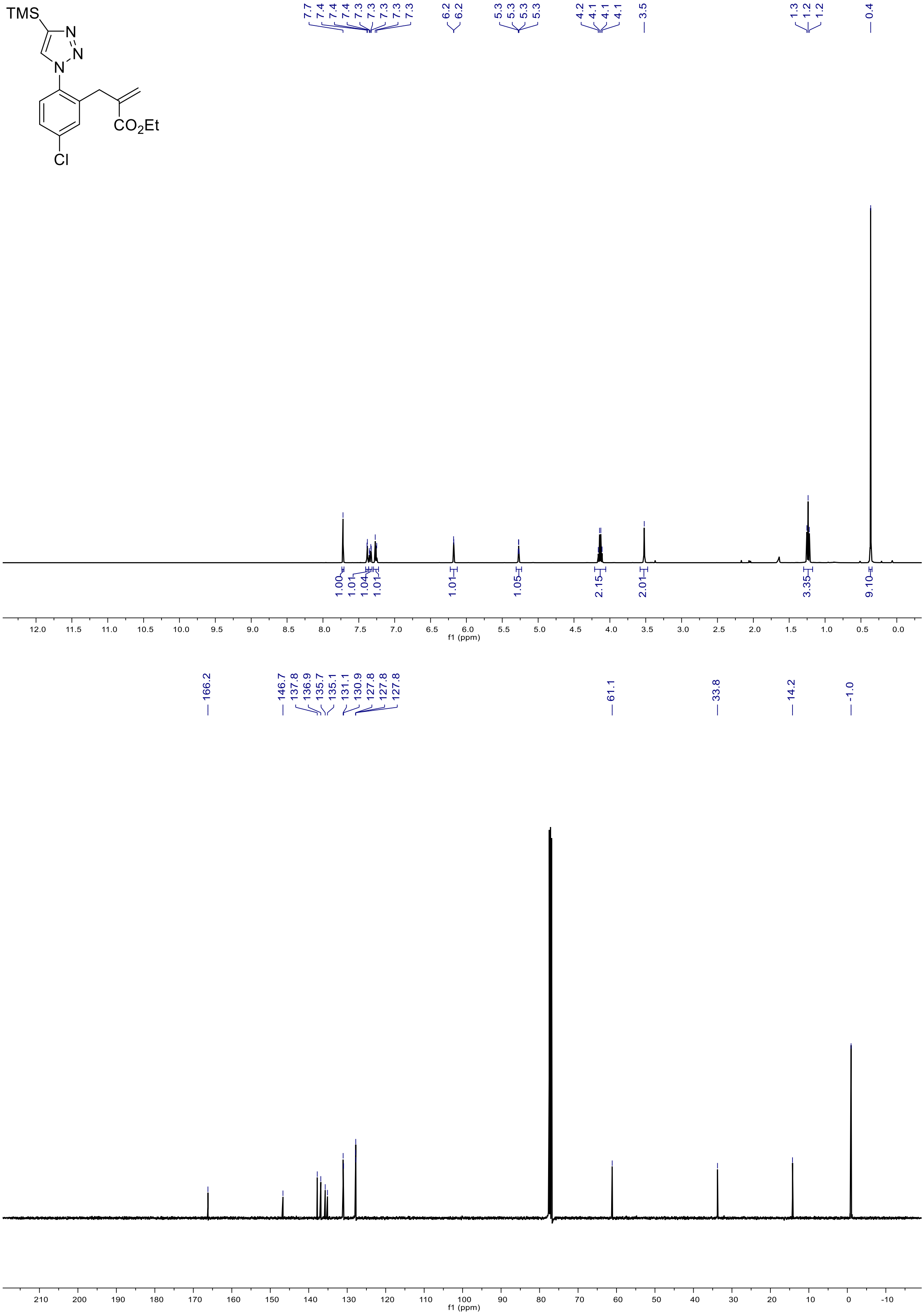

Supplementary Figure 54: ^{1}H-NMR spectrum (top) and ^{13}C-NMR spectrum (bottom) of **7e**.

1-(5-fluoro-4'-(trifluoromethoxy)-[1,1'-biphenyl] -2-yl)-1*H*-1,2,3-triazole (9)

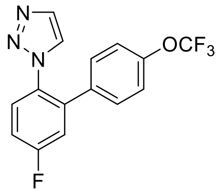

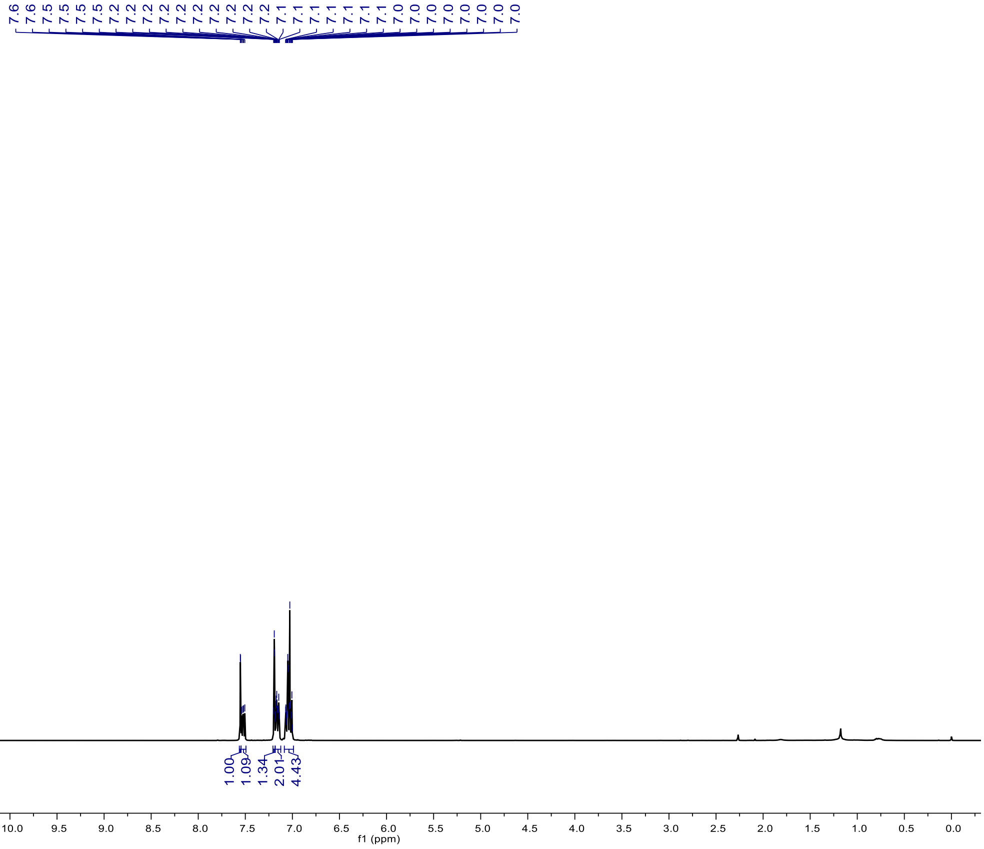

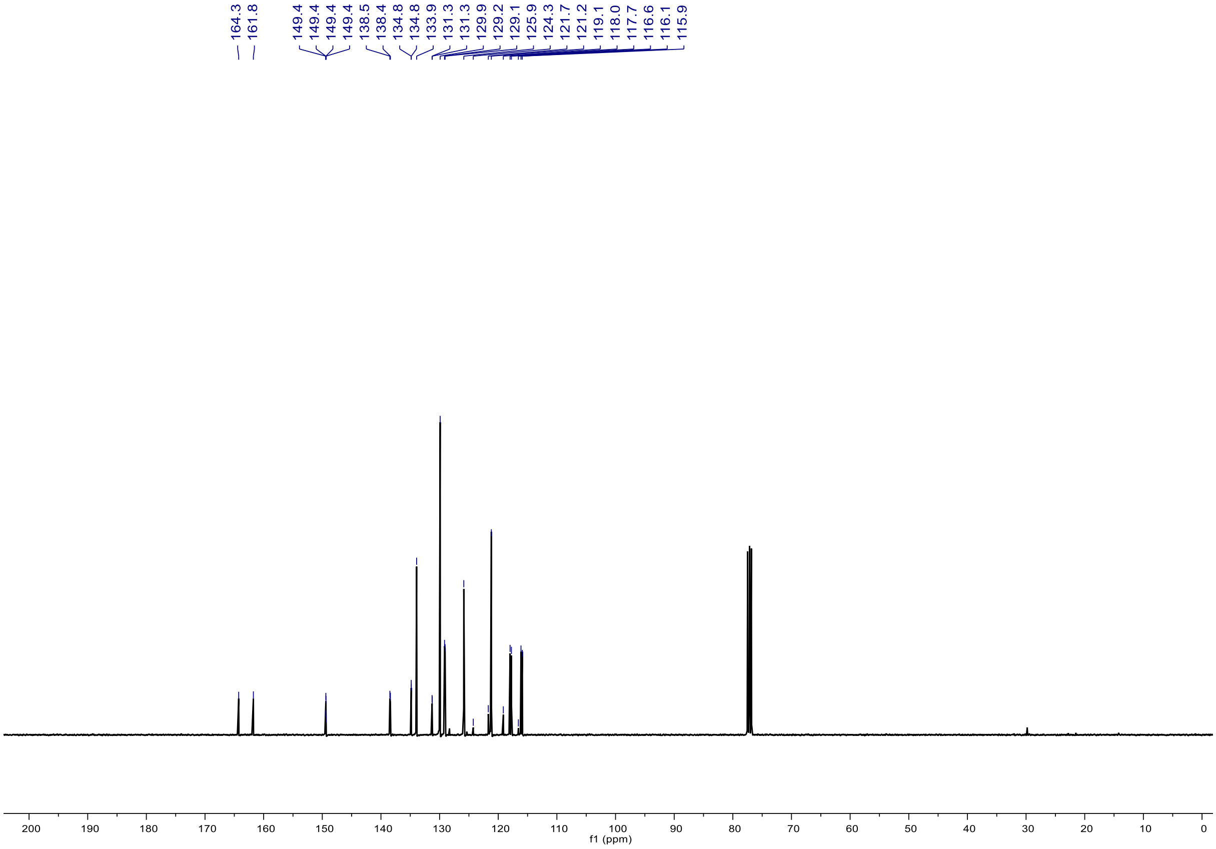

Supplementary Figure 55: ^{1}H-NMR spectrum (top) and ^{13}C-NMR spectrum (bottom) of **9**.

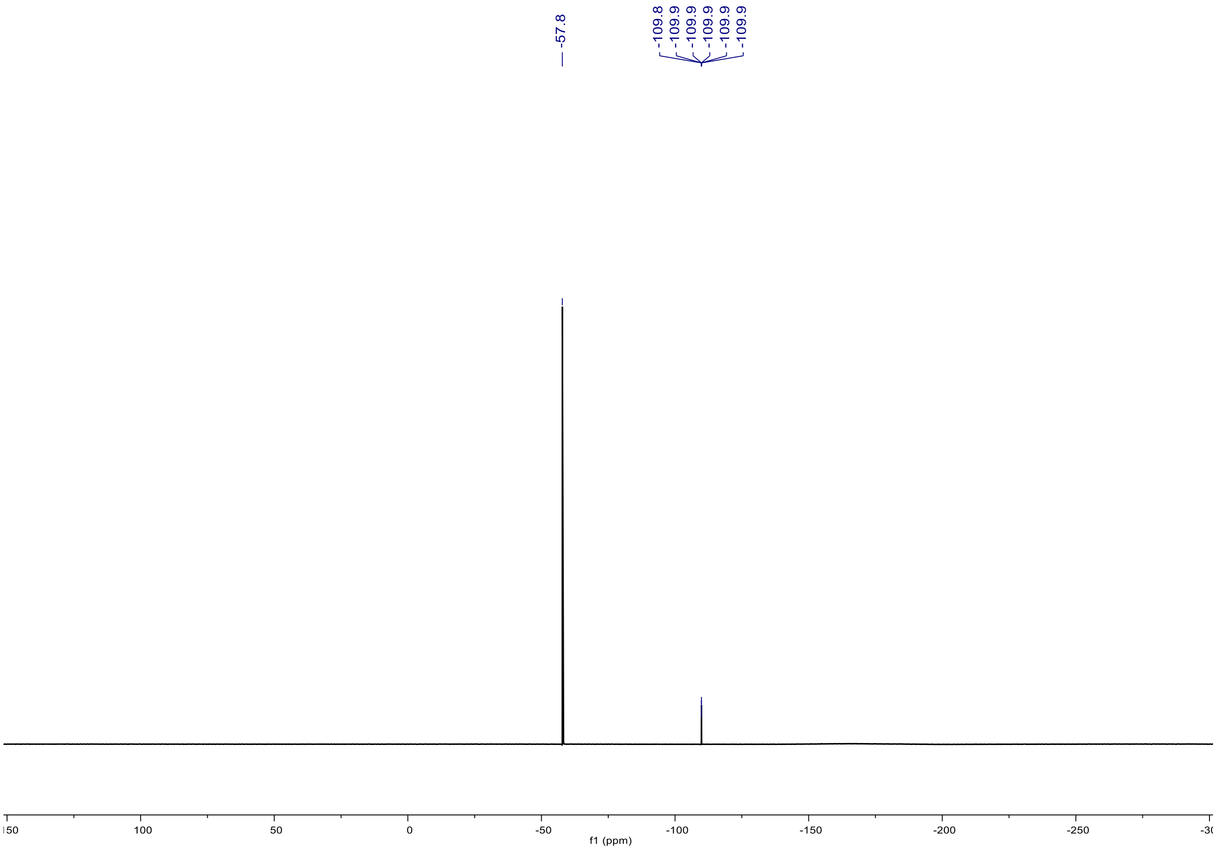

Supplementary Figure 56: ^{19}F-NMR spectrum of **9**.

5-(5-fluoro-4'-(trifluoromethoxy)-2-(4-(trimethylsilyl)-1*H*-1,2,3-triazol-1-yl)-[1,1'-biphenyl]-3-yl)-1-methyl-1*H*-indole (10)

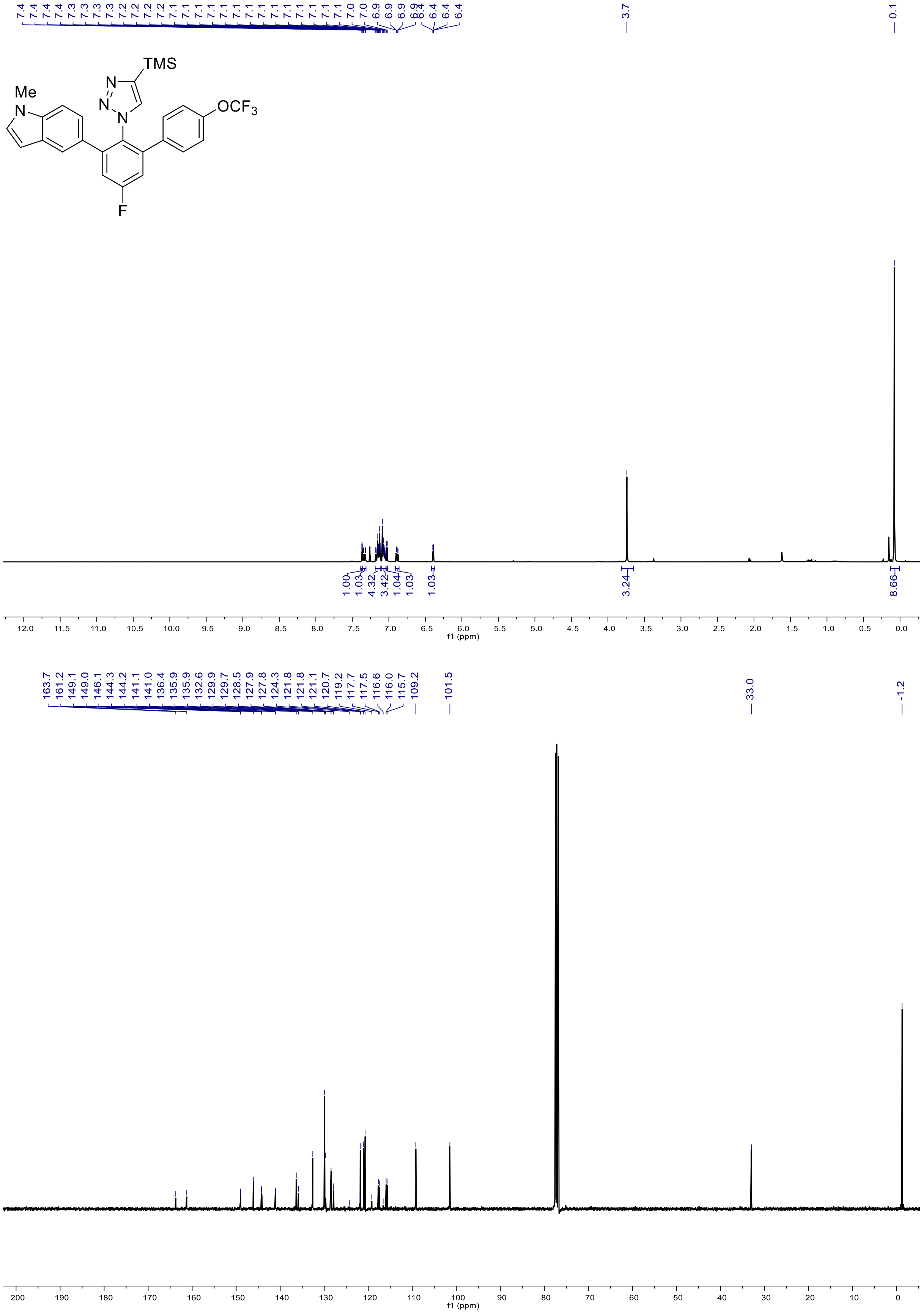

Supplementary Figure 57: ^{1}H-NMR spectrum (top) and ^{13}C-NMR spectrum (bottom) of **10**.

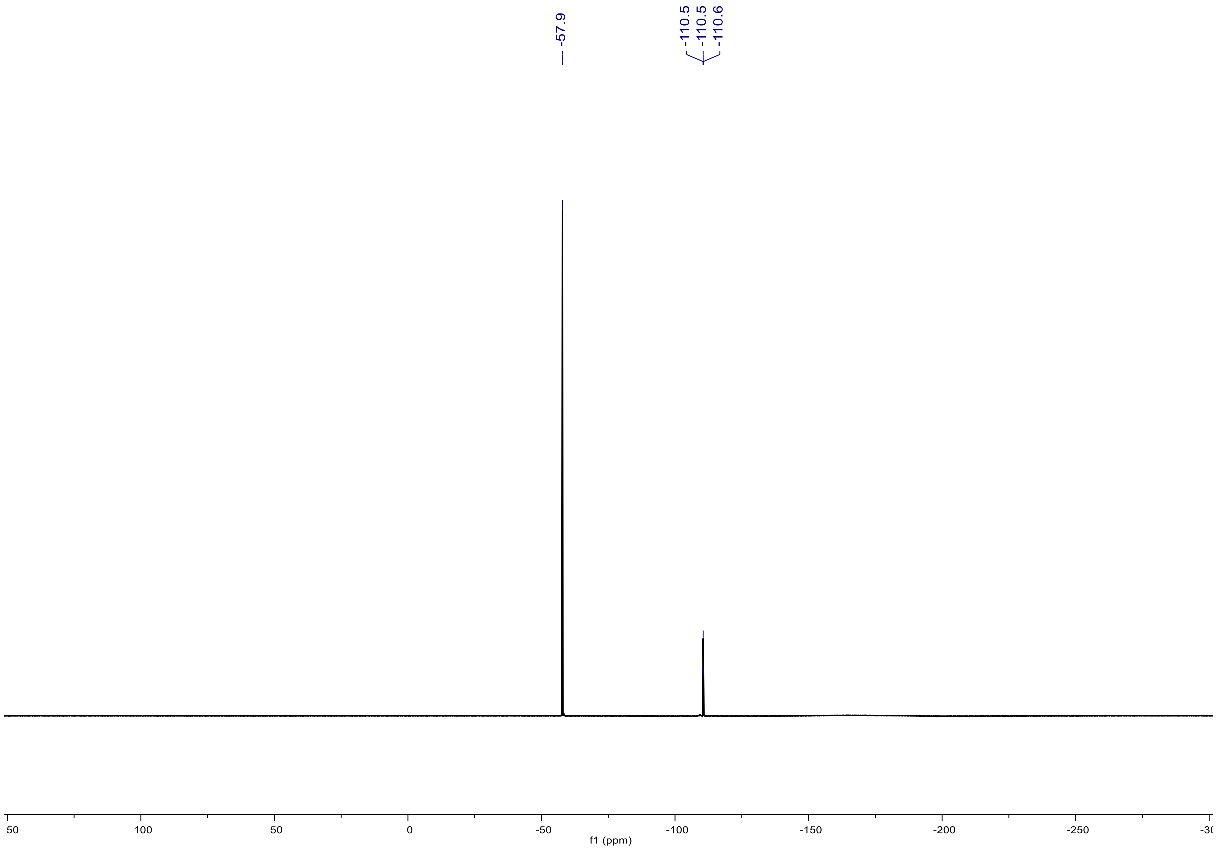

Supplementary Figure 58: ^{19}F-NMR spectrum of **10**.

4-Bromo-1-(5-fluoro-4'-(trifluoromethoxy)-[1,1'-biphenyl]-2-yl)-1*H*-1,2,3-triazole (11)

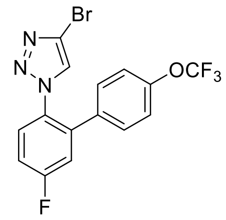

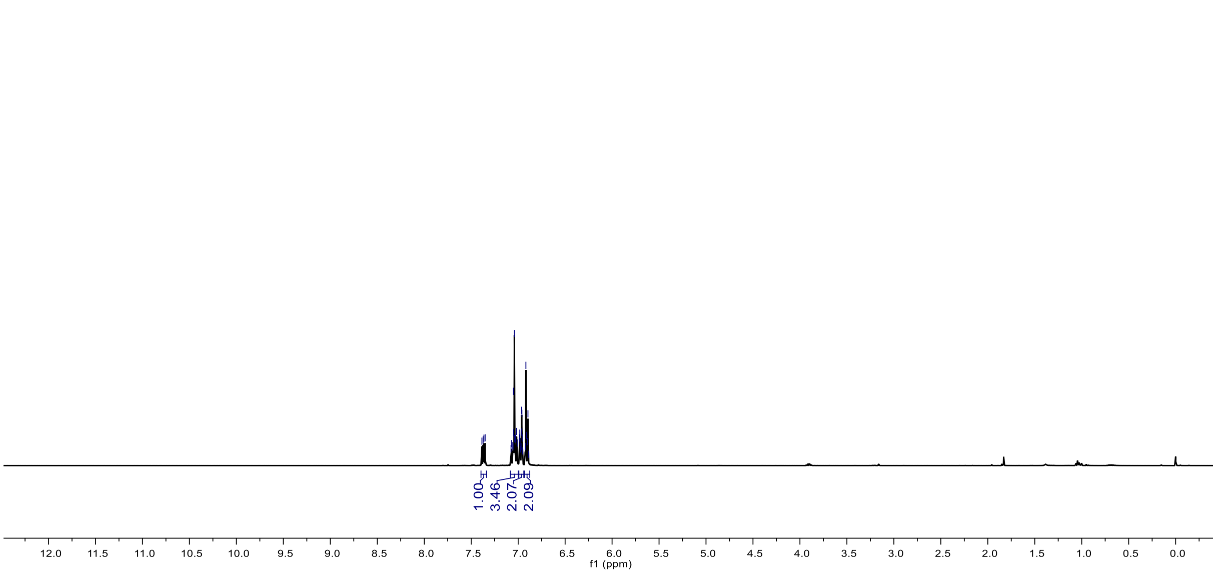

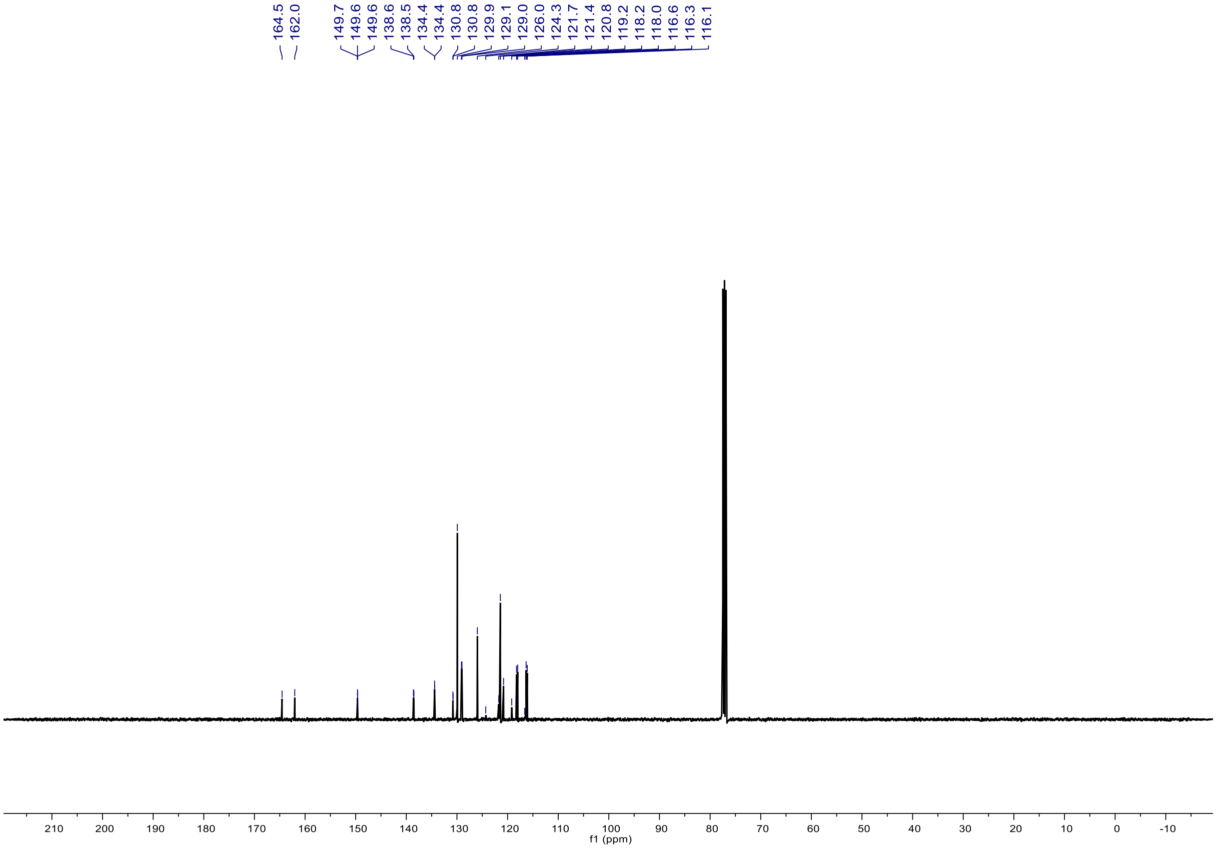

Supplementary Figure 59: ^{1}H-NMR spectrum (top) and ^{13}C-NMR spectrum (bottom) of **11**.

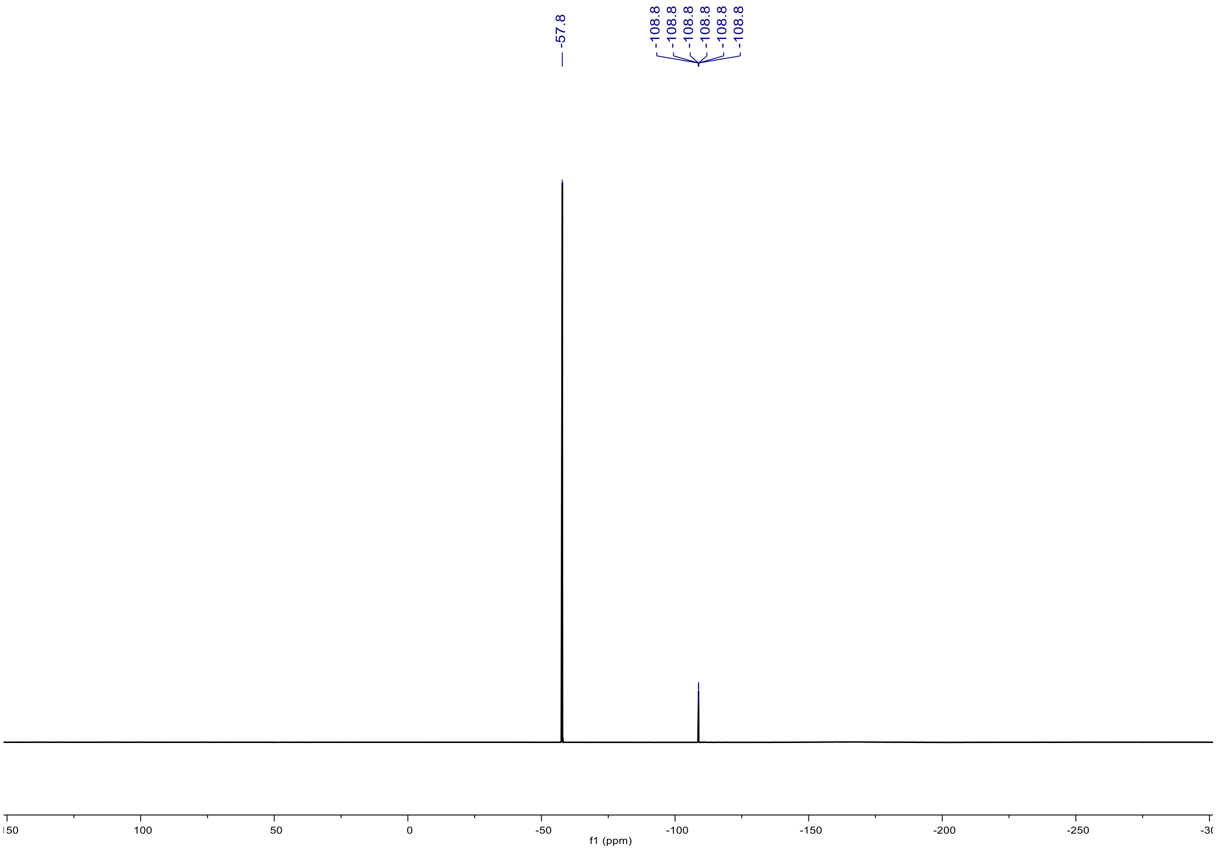

Supplementary Figure 60: ^{19}F-NMR spectrum of **11**.

4-(4-Chlorophenyl)-1-(5-fluoro-4'-(trifluoromethoxy)-[1,1'-biphenyl]-2-yl)-1*H*-1,2,3-triazole (12)

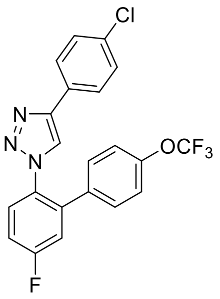

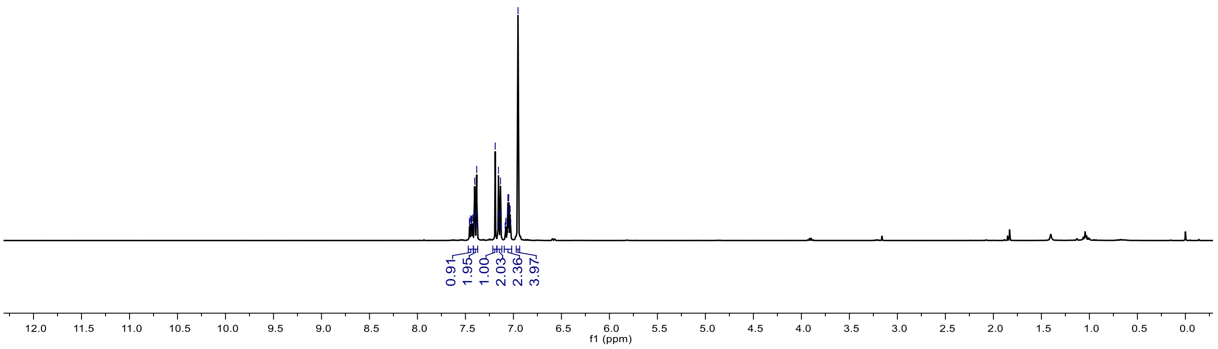

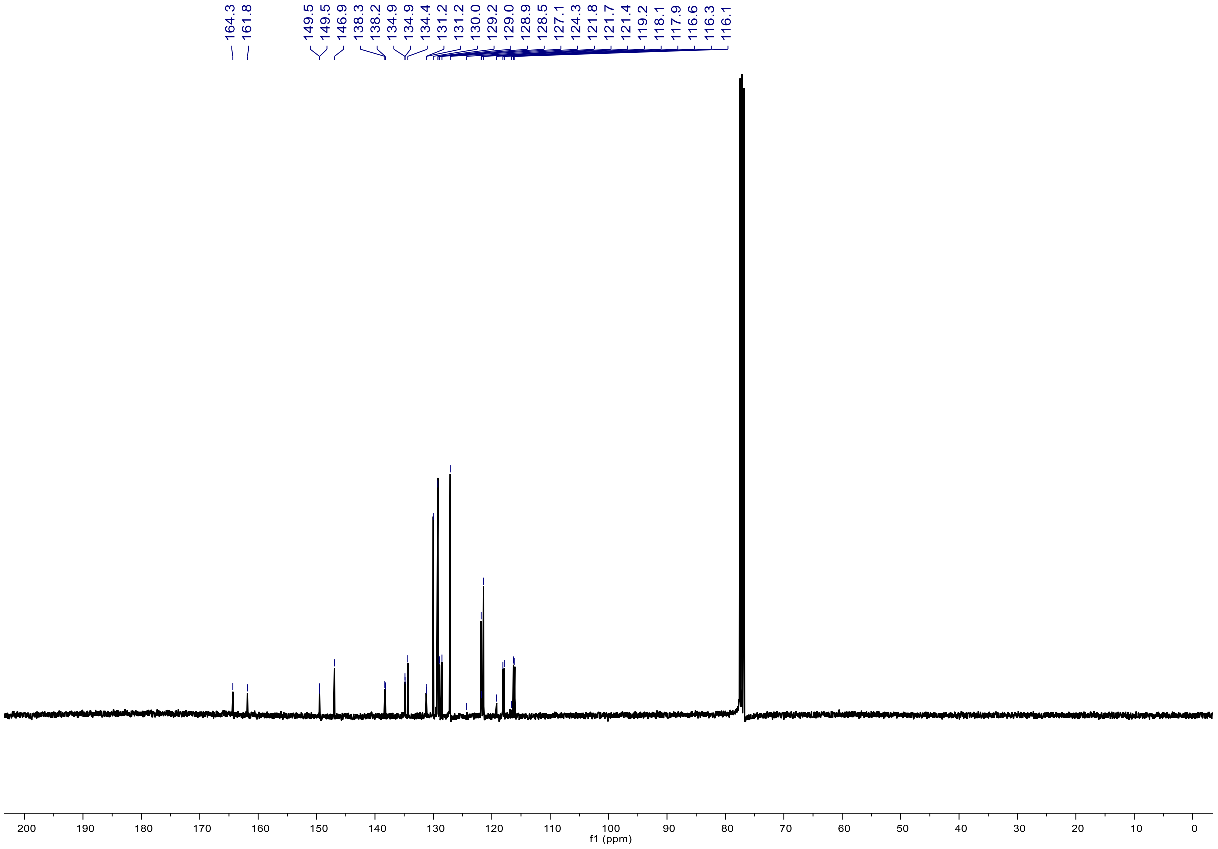

Supplementary Figure 61: ^{1}H-NMR spectrum (top) and ^{13}C-NMR spectrum (bottom) of **12**.

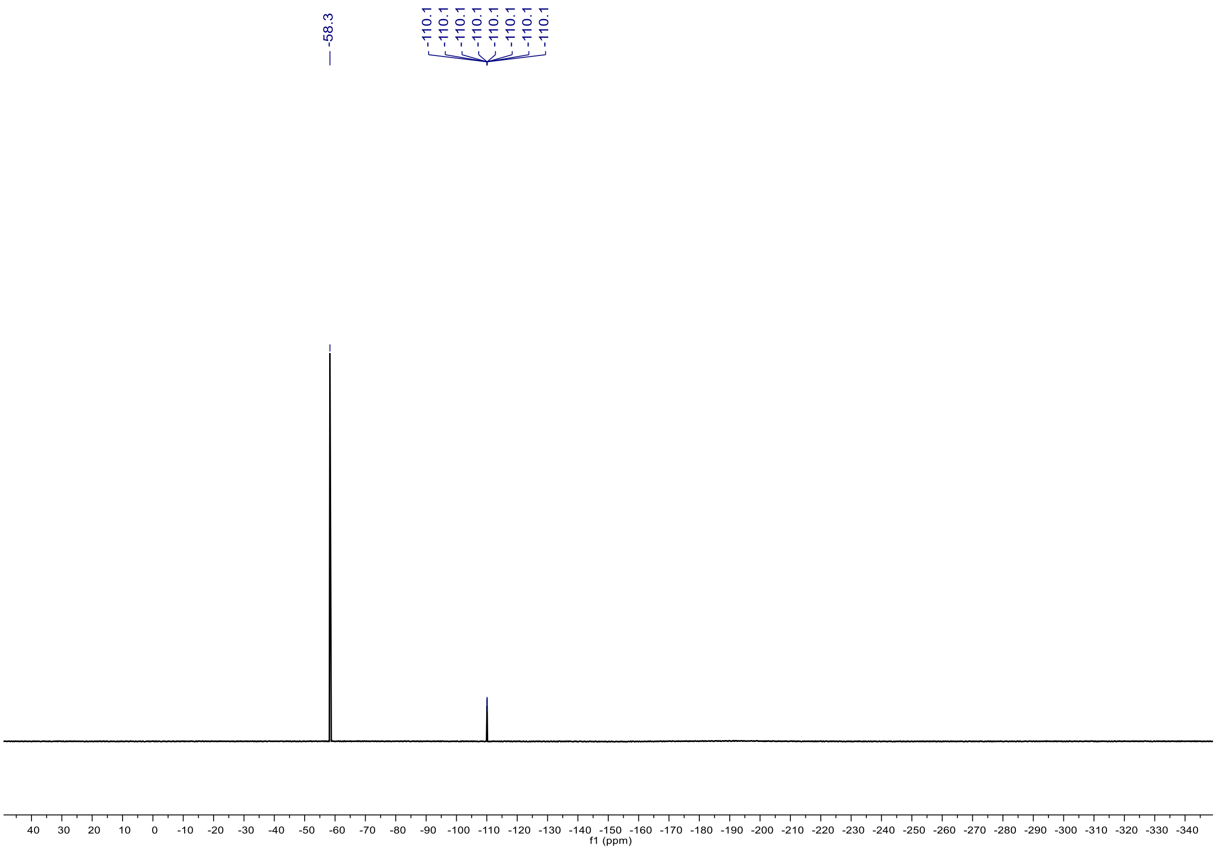

Supplementary Figure 62: ^{19}F-NMR spectrum of **12**.

Supplementary References

[1] Wang, S., Yang, L.-J., Zeng, J.-L., Y. Zheng, Ma, J.-A. Silver-catalyzed [3+2] cycloaddition of isocyanides with diazo compounds: new regioselective access to 1,4-disubstituted-1,2,3-triazoles. *Org. Chem. Front.* **2**, 1468–1474 (2015).

[2] Businelli, S., Di Martino, E., Zanirato, P. New insight on the cycloaddition of aryl and heteroaryl azides with (trimethylsilyl)acetylene. Spectroscopic and x-ray crystallographic data of silylated 1,2,3-triazoles. *ARKIVOC* **1**, 131-143 (2001).

[3] Eisenberger, P., Bestvater, B. P., Keske, E. C., Crudden, C. M. Hydrogenations at Room Temperature and Atmospheric Pressure with Mesoionic Carbene-Stabilized Borenium Catalysts. *Angew. Chem. Int. Ed.*, **54**, 2467–2471 (2015).

[4] Knochel, P., M. Yeh, C. P., Berk, S. C., Talbert, J., Synthesis and reactivity toward acyl chlorides and enones of the new highly functionalized copper reagents RCu(CN)ZnI. *J. Org. Chem.*, **53**, 2390-2392 (1988).

[5] Y. Zhu, A. K. Dilger, W. R. Ewing, M. J. Orwat, D. J. P. Pinto, Factor xia new macrocycle bearing a non-aromatic p2' group, WO 2017019821 A1 (2017).

[6] Jeong, Y., Ryu, J.-S. Synthesis of 1,3-Dialkyl-1,2,3-triazolium Ionic Liquids and Their Applications to the Baylis−Hillman Reaction. *J. Org. Chem.*, **75**, 4183–4191 (2010).

[7] Shan, Z., Peng, M., Fan, H., Lu, Q., Lu, P., Zhao, C., Chen, Y. Discovery of potent dipeptidyl peptidase IV inhibitors derived from β-aminoamides bearing substituted [1,2,3]-triazolopiperidines for the treatment of type 2 diabetes. *Bioorg. Med. Chem. Lett.*, **21**,1731–1735 (2011).
